# Analyses of Genomic tRNA Reveal Presence of Novel tRNAs in *Oryza sativa*

**DOI:** 10.3389/fgene.2017.00090

**Published:** 2017-06-30

**Authors:** Tapan K. Mohanta, Hanhong Bae

**Affiliations:** Department of Biotechnology, Yeungnam UniversityGyeongsan, South Korea

**Keywords:** tRNA, duplication, deletion, *Oryza sativa*, novel tRNA

## Abstract

Transfer rRNAs are important molecules responsible for the translation event during protein synthesis. tRNAs are widespread found in unicellular to multi-cellular organisms. Analysis of tRNA gene family members in *Oryza sativa* revealed the presence of 750 tRNA genes distributed unevenly in different chromosomes. The length of *O. sativa* tRNAs genes were ranged from 66 to 91 nucleotides encoding 52 isoacceptor in total. tRNA^Ser^ found in chromosome 8 of *O. sativa* encoded only 66 nucleotides which is the smallest tRNA of *O. sativa* and to our knowledge, this is the smallest gene of eukaryotic lineage reported so far. Analyses revealed the presence of several novel/pseudo tRNA genes in *O. sativa* which are reported for the first time. Multiple sequence alignment of tRNAs revealed the presence of family specific conserved consensus sequences. Functional study of these novel tRNA and family specific conserved consensus sequences will be crucial to decipher their importance in biological events. The rate of transition of *O. sativa* tRNA was found to be higher than the rate of transversion. Evolutionary study revealed, *O. sativa* tRNAs were evolved from the lineages of multiple common ancestors. Duplication and loss study of tRNAs genes revealed, majority of the *O. sativa* tRNA were duplicated and 17 of them were found to be undergone loss during the evolution. Orthology and paralogy study showed, the majority of *O. sativa* tRNA were paralogous and only a few of tRNA^Ser^ were found to contain orthologous tRNAs.

## Introduction

Transfer RNAs (tRNA) are the most common ancestral short non-coding RNA present in the world and considered one of the single largest gene family in the genome of any species (Mallick et al., 2005; Michaud et al., 2011; Zuo et al., 2013). These molecules can be traced back to the RNA world before the separation of the three major domains of life (Eigen et al., 1989) demonstrating that, all tRNA genes are homologs and derived from an ancestral proto tRNA (Eigen and Winkler-Oswatitsch, 1981). The abundance of tRNA constitutes about 4–10% of all the cellular RNA (Kirchner and Ignatova, 2014). The role of tRNA in life is very important as it forms the interface between the genetic code and amino cids and acts as an adaptor molecule in the protein translation machinery. Additionally, the tRNA is a multifunctional molecule present in the cytoplasm, chloroplasts, and mitochondria (Mallick et al., 2005; Michaud et al., 2011). It has been reported that the tRNA molecules are ~70–100 nucleotides long and form a “clover leaf” like secondary structure and an L-shaped tertiary structure (Torres et al., 2014). The clover like secondary structure is characterized by the presence of an acceptor stem, D-stem, D-loop, anti-codon stem, anti-codon loop, variable loop, TΨC-stem, and TΨC-loop. The important functional part of tRNA is the anti-codon triplet and 3′-CCA nucleotides. The anti-codon triplet reads the messenger RNA (mRNA) codons and an amino acid cognate to tRNA attached to the 3′-CCA nucleotides with the help of aminoacyl tRNA synthetase during the translation events (Goodenbour and Pan, 2006). The aminoacyl tRNA synthetase discriminate its cognate tRNA by the presence of the anti-codon loop and a discriminator base at position 73 (N73) located before the 3′-CCA tail (Kirchner and Ignatova, 2014). tRNA are also involved in the tRNA derived RNA silencing pathway and a major source of short interspersed nuclear elements (SINE) (Bermudez-Santana et al., 2010; Phizicky and Hopper, 2010). During the evolution of tRNA, full and functional tRNA genes were formed as a result of several mutation, duplication, and re-organization events. Although, several basic studies been conducted to investigate the tRNA of lower and higher eukaryotes, only a very little information is available regarding in detailed study of plant tRNAs. The basic details regarding the tRNA biology of plant is very scanty. Therefore, in this study we have investigated the complete genomic details of *Oryza sativa* tRNA and reported here.

## Results and discussion

### *Oryza sativa* tRNAs contain 52 anti-codons

The genome of any given species encodes genes for three major types of RNA, which includes mRNA (messenger RNA), rRNA (ribosomal RNA), and tRNA (transfer RNA). tRNA plays crucial role during the protein synthesis process. Overall, there are 20 different types of essential amino acids present in the genome that forms the backbones of the proteins; hence there are 20 different types of tRNA genes present in the genome to ensure efficient protein synthesis. Genome-wide analysis showed the presence of 750 tRNA in *O. sativa* that encoded 20 standard amino acids (Table 1). In addition to the presence of standard tRNA, the genome of *O. sativa* also encoded 26 pseudo tRNA genes, but no suppressor tRNA was found. The nuclear genome contains multiple tRNA genes encoding an isodecoder tRNA to allow sufficient supply of the tRNA. Earlier, Michaud et al. (2011) reported that, *O. sativa* genome encodes 723 tRNA in total including true tRNA, organellar tRNA and pseudo tRNA (Michaud et al., 2011). The tRNA gene population is very diverse in plants like *O. sativa* relative to the archaea and bacteria. Analyses showed *O. sativa* tRNA contain 52 different anti-codons/isoacceptors in 750 tRNA, with tRNA^Ser^ having the highest abundance of isoacceptors (GCT, TGA, AGA, CGA, and GGA; Table 1). Overall, in total there were 59 tRNA^Arg^ and 60 tRNA^Met^ genes were found in *O. sativa*. The tRNA^Met^ contains only a single isoacceptor CAT, while tRNA^Arg^ contains five different isoacceptors (ACG, TCT, CCT, CCG, and TCG). The abundance of tRNA^Cys^ and tRNA^Trp^ genes in *O. sativa* were found to be the lowest, accounting for only 18 tRNA genes in each. The tRNA^Trp^ contains only one isoacceptor (CCA), while tRNA^Cys^ encodes two isoacceptors (GCA and ACA). The universal genetic code is degenerate where 20 amino acids are encoded by 61 triplet codons (Agris et al., 2007). Therefore, 61 different triplet codes are usually required for 61 different anti-codons/isoacceptors. However, *O. sativa* encodes only 52 isoacceptors and codon degeneracy, codon usage and wobble base pairing might playing significant role in harboring all the codons in the genome of *O. sativa*. *O. sativa* also lacked of a suppressor (CTA and TTA) and selenocysteine (TCA) tRNA. The amino acid selenocysteine is not present universally in all the organisms because of its toxic nature, which might be the reason for its absence in the genome of *O. sativa*. The suppressor tRNAs are mutant form of tRNAs which allow to insert a suitable amino acid at a mutant site in the protein encoding genes and suppresses the phenotypic effect of coding mutation. The suppressor tRNA can affect the production functional cellular protein. Therefore, they are found very rarely in the cells to allow predominance of normal translational event in cell. This might be the reason, why *O. sativa* genome do not have suppressor tRNA. tRNA isoacceptor ACC (glycine), GGG (proline), AAA (phenylalanine), ATT(asparagine), ATG (histidine), ACT (serine), GCG (leucine), GAG (arginine), and GAT (isoleucine were found to be absent from the genome *O. sativa*.

**Table 1:** Detail of *O. sativa* tRNA and anti-codon nucleotide sequences. *O. sativa* encodes maximum of 62 tRNA^Ser^ in its genome with five different anti-codons.

**tRNA**			**Anti-codon**			**Total No. of tRNA**
**POLAR**						
Asparagine	GTT (30)					30
Cysteine	GCA (17)	ACA (1)				18
Glutamine	TTG (21)	CTG (10)				31
Glycine	GCC (28)	TCC (10)	CCC (9)			47
Serine	GCT (20)	TGA (17)	AGA (12)	CGA (9)	GGA (4)	62
Threonine	TGT (16)	AGT (11)	GGT (8)	CGT (5)		40
Tyrosine	GTA (20)	ATA (2)				22
**NON-POLAR**						
Alanine	AGC (20)	CGC (13)	TGC (10)	GGC (1)		44
Isoleucine	AAT (18)	TAT (5)				23
Leucine	CAA (19)	AAG (15)	CAG (10)	TAG (10)	TAA (4)	58
Methionine	CAT (60)					60
Phenylalanine	GAA (20)					20
Proline	AGG (14)	TGG (14)	CGG (9)			37
Tryptophan	CCA (18)					18
Valine	AAC (18)	GAC (17)	CAC (11)	TAC (4)		50
**POSITIVELY CHARGED**						
Arginine	ACG (24)	TCT (12)	CCT (11)	CCG (8)	TCG (4)	59
Histidine	GTG (26)					26
Lysine	CTT (20)	TTT (12)				32
**NEGATIVELY CHARGED**						
Aspartic acid	GTC (31)	ATC (1)				32
Glutamic acid	CTC (25)	TTC (16)				41

*Similarly, it also encodes 60 tRNA^Met^ with only one anti-codon. Only tRNA^Asn^, tRNA^Met^, tRNA^Phe^, and tRNA^Trp^ contain one anti-codon while all other tRNA contains more than one anti-codon*.

### tRNAs are distributed unevenly in different chromosomes

It is important to understand how tRNAs genes are distributed among different chromosome and whether similar iso-acceptors are clustered in the same region of a chromosome. The results of the present study revealed that tRNAs were unevenly distributed in the chromosomes and that a few tRNAs with similar anti-codon were found in a single cluster. Overall, eight tRNA^Gln^ with TTG anti-codon were found in a cluster in the chromosome 2. Additionally, tRNA^Asp^ with GTC anti-codon was found to be in a cluster in the chromosome 5. Other tRNA molecules found in clusters were tRNA^Val^ (AAC), tRNA^Lys^ (CTT), tRNA^Pro^ (AGG), and tRNA^Thr^ (TGT) in chromosome 3, 8, 11, and 12, respectively. The distribution of tRNAs in different chromosome showed that chromosome 1 has encoded maximum number of tRNAs (Table 2) with an average abundance of 4.8 genes for each tRNA. The second highest number of tRNA was encoded by chromosome 4, with an average abundance of 4.76 genes for each tRNA. Similarly, the lowest number of tRNA genes was encoded by chromosome 11, with an average richness of 1.61 genes for each tRNA. The richness of tRNA^Arg^, tRNA^Met^, and tRNA^Ser^ was higher relative to other tRNAs (Table 1). The result showed, the maximum numbers of tRNA^Arg^ genes (11) were encoded in chromosome 3 (Table 2). Similarly, chromosome 12 encoded higher numbers of tRNA^Met^ (11) while chromosome 2 encoded higher numbers of tRNA^Ser^ (11). Although, different tRNAs were encoded by different chromosomes, chromosome 6 did not encode tRNA^Asn^, tRNA^Cys^, tRNA^Tyr^, tRNA^Ile^, and tRNA^Arg^ (Table 2). Similarly, chromosome 9 did not encode tRNA^Gln^, tRNA^Met^, and tRNA^Trp^ tRNA whereas chromosome 11 did not encode tRNA^Cys^, tRNA^Gln^, tRNA^Thr^, tRNA^Tyr^, tRNA^Phe^, and tRNA^Trp^. However, the exact reasons for the absence of a specific tRNA gene from a particular chromosome are difficult to delineate. Particularly, it is not clear why certain chromosomes did not encoded all of the required tRNA and more specifically, why chromosome 11 lacked genes six tRNA genes family. The genes of common evolutionary origin and similar function often clustered together. This might be the reason why tRNAs were found in cluster in chromosome 2, 3, 5, 8, 11, and 12.

**Table 2:** Chromosomal distribution of different tRNA in *O. sativa*.

**tRNA**	**Chr1**	**Chr2**	**Chr3**	**Chr4**	**Chr5**	**Chr6**	**Chr7**	**Chr8**	**Chr9**	**Chr10**	**Chr11**	**Chr12**
**POLAR AMINO ACIDS**												
Asparagine	5	1	5	3	4	0	1	1	1	4	1	3
Cysteine	2	4	1	5	1	0	1	0	1	2	0	1
Glutamine	4	11	3	3	1	3	1	1	0	2	0	2
Glycine	6	5	8	4	4	2	6	4	1	2	1	4
Serine	6	11	4	9	5	4	7	2	1	5	3	4
Threonine	5	4	5	6	1	2	1	1	2	5	0	7
Tyrosine	3	3	2	4	1	0	0	3	2	2	0	1
**NON-POLAR**												
Alanine	4	5	5	5	1	5	2	3	2	3	4	4
Isoleucine	6	1	1	4	2	0	1	2	2	2	1	1
Leucine	9	6	6	6	4	5	3	4	5	4	3	3
Methionine	6	8	6	8	2	3	3	1	0	7	1	11
Phenylalanine	7	2	0	2	1	1	1	1	1	2	0	2
Proline	2	1	2	1	1	4	2	2	2	1	10	9
Tryptophan	1	3	2	3	0	2	1	3	0	2	0	1
Valine	5	2	6	11	5	1	1	5	4	3	2	2
**POSITIVELY CHARGED**												
Arginine	8	0	11	8	8	0	1	6	4	6	2	5
Histidine	1	1	2	6	1	1	4	2	2	4	1	1
Lysine	6	1	1	1	4	2	0	3	3	4	2	5
**NEGATIVELY CHARGED**												
Aspartic acid	5	2	3	3	7	2	3	1	2	2	1	1
Glutamic acid	7	5	5	4	4	1	3	4	2	1	2	3
**PSEUDO tRNA**												
	3	1	3	4	2	2	2	3	2	3	0	1
Average	4.80	3.66	3.85	4.76	2.80	1.95	2.09	2.47	1.87	3.14	1.61	3.88

*Chromosome 1 and chromosome 4 encodes higher numbers of tRNA while chromosome 11 encodes lowest number of tRNA. tRNA^Gln^, tRNA^Ser^, and tRNA^Met^ are abundantly present in chromosome 3 while tRNA^Met^, tRNA^Ser^, tRNA^Val^, and tRNA^Arg^ are abundantly found in chromosome 4. tRNA^Asn^, tRNA^Cys^, tRNA^Tyr^, tRNA^Ile^, and tRNA^Arg^ were absent in chromosome 6. Similarly tRNA^Gln^, tRNA^Met^, and tRNA^Try^ were absent from chromosome 9 while tRNA^Cys^, tRNA^Gln^, tRNA^Thr^, tRNA^Tyr^, tRNA^Phe^, and tRNA^Try^ absent from chromosome 11*.

### The length of *O. sativa* tRNAs varied from 66 to 91 nucleotides

**Figure 1:**
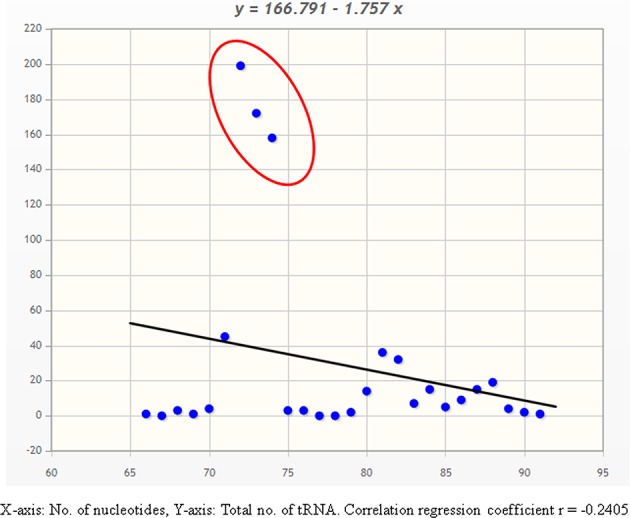
Correlation-regression analysis of number of nucleotides vs. total number of tRNAs in *O. sativa*. Analysis shows, majority of tRNA contains 70–75 nucleotides.

The 20 different types of tRNAs encode for 61 unique codons in the genome. Therefore, it can be speculated that, the length and composition of different tRNAs will also vary significantly. Different researchers have reported varied findings regarding the length of tRNAs. Kirchner and Ignatova (Kirchner and Ignatova, 2014) reported that tRNAs were of 73–90 nucleotides length, while Goodenbour and Pan (2006) reported that tRNAs were of 75–95 nucleotides length. Furthermore, Schimmel and Tamura (2003) reported that, tRNAs were of 76 nucleotides length (Schimmel and Tamura, 2003), similar to Mallick et al. (2005). However, majority of these studies were based on tRNA of animal/bacterial species, while little information is available regarding the plant tRNA. In this study, we found that the *O. sativa* tRNAs varied from 66 to 91 nucleotides (Table 3). One gene of tRNA^Ser^ (chr8.tRNA1) encoded only 66 nucleotides whereas tRNA^Met^ (chr3.tRNA35) encoded 91 nucleotides (Table 3). Moreover, three tRNA^Met^ genes encoded 68 nucleotides and one tRNA^Ser^ gene encoded 69 nucleotides. To the best of our knowledge, tRNA^Ser^ of chromosome 8 having 66 nucleotides is the smallest gene in eukaryotic kingdom reported so far. With the exception of a few tRNA, the majority tRNA of *O. sativa* reside within 72–74 nucleotides length (Figure 1, Table 3). Additionally, all of the tRNA^Gln^ were found to be 72 nucleotides, whereas all the tRNA^Ile^ were found to be 74 nucleotides. Unlike tRNA^Gln^ and tRNA^Ile^, all of the tRNA^Ala^ and tRNA^Phe^ were found to be 73 nucleotides. Moreover, all the tRNA^Tyr^ and tRNA^Leu^ were found to be 80 nucleotides or more. None of a single tRNA^Tyr^ and tRNA^Ile^ was found to be below 80 nucleotides in length. The presences of varied number of nucleotides found in tRNA are most probably necessary for their interaction with the cognate aminoacyl-tRNA synthetase for proper function. This suggests that amino-acyl tRNA synthetases of the respective tRNA are structurally different and hence it requires different nucleotide composition for each of them for their proper interactions.

**Table 3:** Nucleotide length of different tRNAs in *O. sativa*.

**tRNA**	**No. of nucleotides**																						
	**66**	**68**	**69**	**70**	**71**	**72**	**73**	**74**	**75**	**76**	**79**	**80**	**81**	**82**	**83**	**84**	**85**	**86**	**87**	**88**	**89**	**90**	**91**
**POLAR**																							
Asn						15		15															
Cys					6	9		1															
Gln						31																	
Gly				1	36	10																	
Ser	1		1							2		1		32					6	18	1		
Thr						18	9	11	1			1											
Tyr															3	6	3	8	2				
**NON-POLAR**																							
Ala					1		42												2				
Ile								23															
Leu												10	36		4	8							
Met		3				12	10	20								1	2	1	4	1	3	2	1
Phe							19					1											
Pro						29		7	1														
Trp						12	1	5															
Val						17	4	28											1				
**POSITIVELY CHARGED**																							
Arg					2	4	37	15	1														
His						11		15															
Lys				2		6	22			1		1											
**NEGATIVELY CHARGED**																							
Asp				1		23		8			1												
Glu						2	28	10			1												

*Most of the tRNAs are 72, 73, or 74 nucleotides length. All of the tRNA^Tyr^ are more than 83 nucleotide length whereas, tRNA^Met^ are distributed evenly*.

### *O. sativa* tRNA contain introns

**Figure 2:**
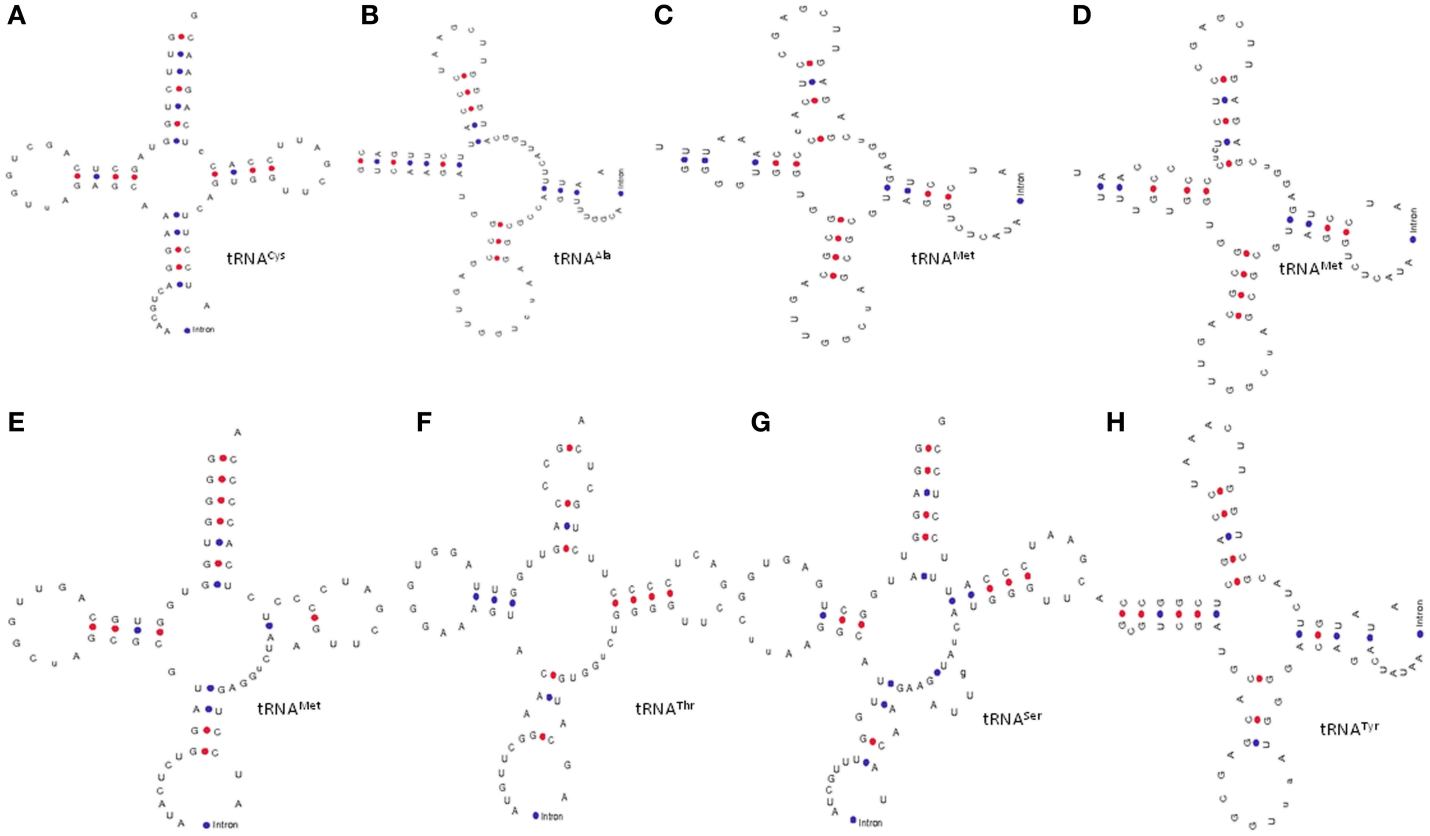
General structures of intron containing tRNAs. Earlier it was reported that only tRNA^Met^ and tRNA^Tyr^ contains introns in higher plants. However, we found that **(H)** tRNA^Tyr^; **(G)** tRNA^Ser^, **(A)** tRNA^Cys^, **(F)** tRNA^Thr^, and **(B)** tRNA^Ala^ contain introns in additional to **(C–E)** tRNA^Met^. The introns in tRNA are usually found in the anti-codon loop. Adenine nucleotide is the predominant nucleotide before the start site of introns in all tRNA.

**Figure 3:**
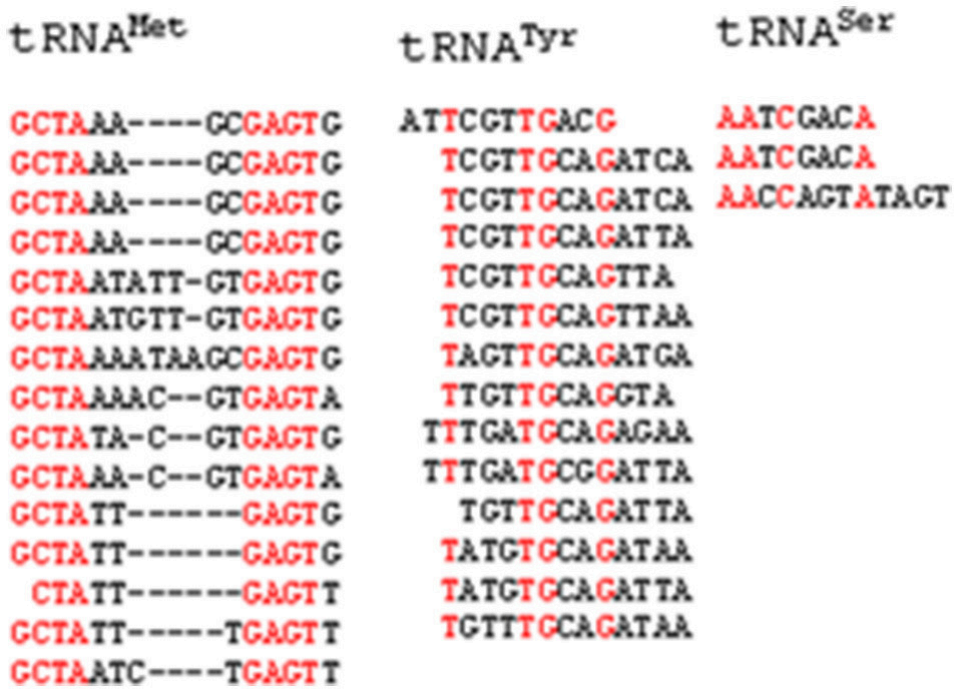
Conserved consensus sequence of intron of *O. sativa* tRNA. tRNA^Met^ contains G-C-T-A and G-A-G-T, tRNA^Tyr^ contains x-T-x_3_-T-G-x_2_-G and tRNA^Ser^ contains A-A-x-C-x_3_-A conserved consensus sequence.

Introns are well-known entities of the genome that interrupts the continuity of many eukaryotic genes. Introns are reportedly present in the tRNA of all three kingdoms of life (Abelson et al., 1998; Yoshihisa, 2014). More specifically, the eukaryotic and the archaeal genomes are reported to be good sources of tRNA introns. We also found that, *O. sativa* tRNAs contain introns. In total, 34 (4.53%) tRNAs of *O. sativa* were found to contain introns (Table 4). Michaud et al. (2011) reported that the *O. sativa* genome possessed only 27 introns containing tRNA (Michaud et al., 2011). However, in our study we found that 34 tRNA genes of *O. sativa* contained introns. From a total of 60 tRNA^Met^, 15 (25%) tRNA genes contained introns, which was the highest number of intron possessed by a tRNA family in *O. sativa*. Unlike tRNA^Met^, 14 (63.63%) of the 22 tRNA^Tyr^ genes were found to contain introns. In addition to tRNA^Met^ and tRNA^Tyr^, two tRNA^Cys^, one tRNA^Ser^, and one tRNA^Thr^ were found to contain intron. Early study reported that only two tRNA family members tRNA^Met^ and tRNA^Tyr^ contained introns in higher plants (Michaud et al., 2011). However, in our study we found that RNA^Ala^, tRNA^Cys^, tRNA^Ser^, and tRNA^Thr^ also contain intron. It was previously believed that most of the intron containing tRNAs in archaea and eukaryotes possessed intron at a canonical position, one nucleotide 3′ to the anti-codon loop. Our study also shows the presence of introns at a canonical position 3′ to the anti-codon loop. However, a few intron containing tRNA genes were shown to have very unusual structures (Figure 2). Several of the intron containing tRNAs showed hairpin like structures in either the acceptor arm, D-arm, anti-codon arm, or T-arm. However, intron was present at the anti-codon loop in the majority of cases (Figure 2). Whiles a few tRNA contained one hairpin loop, other contained more than one hairpin loop. Tocchini-Valentini et al. (2009) reported that ~75% of all tRNA introns were found at the canonical position (37/38 position) and about 25% of tRNA introns were located at noncannonical positions including the anti-codon loop, anti-codon stem, variable loop, D-and T-arms, and acceptor stem (Tocchini-Valentini et al., 2009). The presence of unusual and hairpin like bulged nucleotide sequences in different parts of tRNA represents the characteristics structure of split tRNA (Sugahara et al., 2006). The presence of several bulge like helix-loop tRNA structure reflect the presence of split tRNA in *O. sativa*. The tRNA^Ala^ (chr1.tRNA34) contained only one intron; however, it was the largest intron containing tRNA. Moreover, it possessed 125 nucleotides, making it larger than the tRNA itself, while tRNA^Ser^ (chr12.tRNA22 and chr9.tRNA4) was the smallest intron containing tRNA that contained only eight nucleotides. The intron of tRNA^Met^ was around 17 nucleotides length and contained a conserved G-C-T-A and G-A-G-T nucleotide consensus sequences (Figure 3). The G-C-T-A conserved consensus sequence was present at the 5′ end of the intron and the G-A-G-T consensus sequence was found to present one nucleotide up-stream to the 3′ end of the intron (Figure 3). The G-A-G-T nucleotides are responsible for base pairing that needed to maintain the proper structure of pre-tRNA^Met^ which is pre-requisite for intron splicing (Akama et al., 2000). The presence of intron 3′ to the cannonical anti-codon position is usually found in plastidal tRNA which is self-splicing group I introns (Tocchini-Valentini et al., 2009). These introns are very long and sometimes longer than the sequence of tRNA itself (Sugahara et al., 2006; Tocchini-Valentini et al., 2009). The intron of tRNA^Tyr^ was around 15 nucleotides length and contained a conserved 5′-x-T-x_3_-T-G-x_2_-G-3′ nucleotide sequence (Figure 3). The tRNA^Ser^ was found to contain conserved 5′-A-A-x-C-x_3_-A-3′ sequences in its intron. These conserved structural features of introns are important to *in-vitro* intron splicing (Stange et al., 1992; Akama et al., 2000). Mutation in the cytosine residue at either the 5′ or 3′ end of the intron border leads to deleterious effects on maturation of tRNA and involvement of the D-stem and variable loop (Stange et al., 1992; Akama et al., 1997, 2000). The intron sequence of pre-tRNA^Met^ was conserved more than the intron sequence of pre-tRNA^Tyr^. The introns of pre-tRNA^Ser^ genes were found to contain similar sequences. In tRNA^Tyr^, only T was found to be conserved at the first or second position, T-G at the fifth and sixth position and G nucleotide at 0–4 nucleotides upstream of the 3′ end of the intron (Figure 3). The T residue of the intron at the first or second position is most important for formation of pseudouridine at the second position of the anti-codon loop (Akama et al., 1997). The intron in tRNA^Ala^ was found in between position 39 and 40 while the position of the intron in tRNA^Cys^, tRNA^Met^, and tRNA^Tyr^ was present in between nucleotides 38 and 39. The intron of tRNA^Thr^ was found between nucleotides 36 and 37, while the intron of tRNA^Ser^ was found in between 37 and 38 nucleotides. It was previously believed that only tRNA^Met^ and tRNA^Tyr^ contained introns (Michaud et al., 2011). However, the results of the present study demonstrated that tRNA^Thr^, tRNA^Ala^, tRNA^Cys^, and tRNA^Ser^ also contain introns (Table 4). This is a quite novel finding and further studies of other plant species may lead to identification of additional intron containing tRNA in the plant kingdom.

**Table 4:** Intron containing tRNAs of *O. sativa*.

**tRNA**	**No. of intron containing tRNA**
Asparagine	0
Cysteine	2
Glutamine	0
Glycine	0
Serine	1
Threonine	1
Tyrosine	14
Alanine	1
Isoleucine	0
Leucine	0
Methionine	15
Phenylalanine	0
Proline	0
Tryptophan	0
Valine	0
Arginine	0
Histidine	0
Lysine	0
Aspartic acid	0
Glutamic acid	0

*In total 34 (2.02%) tRNAs contain intron from a total of 750 tRNA. tRNA^Met^ and tRNA^Tyr^ contain higher numbers of intron*.

### *O. sativa* tRNA are varied in their structure

It was recently reported that, the nuclear encoded eukaryotic tRNA and bacterial tRNA that play important roles in translation have a length of 73–90 nucleotides (Kirchner and Ignatova, 2014). The acceptor arm is seven bp long, the D-stem ranges from 3 to 4 bp, and the anti-codon stem is of 5 bp (Kirchner and Ignatova, 2014). The D-loop occasionally varied from 4 to 12 nucleotides. The anti-codon loop is always numbered from 34 to 36, whereas the variable region always starts at residue 44 and is 4–23 nucleotides long (Kirchner and Ignatova, 2014). The Ψ-loop possesses seven nucleotides and the Ψ-stem possesses five nucleotides. The CCA tail is always numbered 74–76 (Kirchner and Ignatova, 2014). However, in our study we found several variations in the tRNA of *O. sativa*. The majority of the acceptor arm was found to contain seven nucleotides whereas; a few were found to contain less than seven nucleotides. tRNA^Met^22 (Chr10), tRNA^Met^57 (Chr10), and tRNA^Met^96 (Chr4) were found to contain three nucleotides whereas tRNA^Arg^92 (Chr1), and tRNA^Ser^20 (Chr2) were found to contain four nucleotides and tRNA^Met^21 (Chr12), tRNA^Asn^43 (Chr8), and tRNA^Ser^27 (Chr3) were found to contain six nucleotides in the acceptor arm (Supplementary Table 1). The D-stem usually contains 3–4 nucleotides. In our study we found that, a total of 501 tRNA genes were contained four nucleotides (66.97%) and 234 contained three nucleotides (31.28%) in the D-stem. All of the tRNA^Lys^, tRNA^Asp^, and tRNA^Trp^ were found to contain four nucleotides in the D-stem whereas all other tRNAs were found to contain either three or four nucleotides. Only one tRNA^Leu^ gene was found to contain four nucleotides in the D-stem. Additionally, one gene from each of the tRNA^Ala^, tRNA^Asn^, tRNA^Glu^, tRNA^Ile^, tRNA^Phe^, and tRNA^Thr^ were found to contain three nucleotides in the D-stem, whereas all of the tRNA^Leu^ were found to contain three nucleotides in the D-stem. Few tRNAs were found to contain less than three nucleotides in the D-stem. tRNA^Leu^34 (Chr9), tRNA^Leu^21 (Chr11), tRNA^Leu^38 (Chr9), tRNA^Pro^71 (Chr12), tRNA^Tyr^49 (Chr8), tRNA^Ser^27 (Chr3), tRNA^Ser^1 (Chr8), tRNA^Tyr^34 (Chr1), and tRNA^Arg^31 (Chr5) were found to contain only two nucleotides in the D-stem. The D-stem was found to be absent in tRNA^Ser^27 (Chr3) and tRNA^Ser^1 (Chr8) (Supplementary Table 1). The D-stem helps to stabilize the tertiary structure of the tRNA and the presence of more nucleotides make the tRNA more stable compared to those which contained less nucleotide. Therefore, the majorities of tRNAs were found to possess four nucleotides in the D-stem.

Previously it was reported that the D-loop contain 4–12 nucleotides (Kirchner and Ignatova, 2014); however, in the present study the D-loop was found to contain 0–15 nucleotides (Supplementary Table 1). tRNA^Glu^11 (Chr7) was found to contain15 nucleotides, whereas tRNA^Lys^7 (chr12), tRNA^Tyr^34 (chr1), and tRNA^Tyr^49 (chr8) were found to contain 13 nucleotides each in the D-loop (Supplementary Table 1). The majority of tRNA were found to contain seven (113), eight (227), or nine (212) nucleotides in the D-loop, whereas only tRNA^Ser^43 (chr7) was found to contain four nucleotides. The D-loop was found to be absent from tRNA^Ser^27 (chr3) and tRNA^Ser^1 (chr8). When the tRNA specific nucleotide composition in the D-loop was considered, tRNA^Leu^ was found to possess 10 (14), 11 (18), or 12 (23) nucleotides in the D-loop. Several tRNA^His^ were found to contain either 8 or 11 nucleotides; tRNA^Met^ contained 7, 8, 9, 10, or 11 nucleotides; tRNA^Pro^ contained 7, 9, 10, or 11 nucleotides; tRNA^Val^ contained 7, 10, or 11 nucleotides; tRNA^Ser^ contained 8, 9, 10, or 11; tRNA^Arg^ contained 8, 9, or 10 nucleotides, tRNA^Glu^ contained 8, 9, or 10 nucleotides, tRNA^Asn^ contained 7 or 9 nucleotides, tRNA^Asp^ contained 8 or 9 nucleotides, tRNA^Cys^ contained 7 or 9 nucleotides, tRNA^Gln^ contained 8 or 9 nucleotides, tRNA^Gly^ contained 7, 8, or 9 nucleotides, tRNA^Thr^ contained 7, 8, or 9 nucleotides, tRNA^Trp^ contained 7 or 9 nucleotides, tRNA^Tyr^ contained 8 or 9 nucleotides, tRNA^Ala^ and tRNA^Lys^ contained 7 or 8 nucleotides, tRNA^Ile^ contained 9 nucleotides and tRNA^Phe^ contained nucleotides in the D-loop. The absence of the D-arm and the D-loop tRNA reveal that, it is a novel form of tRNA and laboratory based experimental study of these tRNA will reveal their functional significance.

It is well-known that the anti-codon arm possesses five nucleotides; however we found a few variations in this region. Specifically, tRNA^Ala^43 (chr1) and tRNA^Tyr^49 (chr8) were found to contain only three nucleotides in their anti-codon arm (Supplementary Table 2), while at least 22 genes of tRNA^Met^ were found to contain four nucleotides in this region. Other tRNA those were found to contain four nucleotides in the anti-codon arm were tRNA^Phe^83 (chr1), tRNA^Glu^11 (chr7), tRNA^Ser^27 (chr3), tRNA^Ser^68 (chr1), and tRNA^Ser^1 (chr4). The anti-codon loop possess seven nucleotides as do the majority of *O. sativa* tRNA. However, tRNA^Arg^17 (chr7) was found to contains eight nucleotides, tRNA^Gln^30 (chr6) contained nine, tRNA^Tyr^49 (chr8) contained 12, and tRNA^Ser^68 (chr1) contained 13 nucleotides. These variations in the nucleotide composition in the anti-codon loop might be responsible for the coding of novel protein translation machinery that has yet to be elucidated.

The variable region of tRNA is very dynamic and possess 4–23 nucleotides (Kirchner and Ignatova, 2014). The variable region of the tRNA of *O. sativa* was found to contain 2–20 nucleotides. The majority of tRNA were found to contain five (457; 60.93%) nucleotides in the variable region. Moreover, approximately 138 tRNA were found to contain four nucleotides in the variable region that constitutes 18.44% of the total tRNA. Only 10 tRNA were found to contain six or seven nucleotides in the variable region (Supplementary Table 2). All tRNA^Leu^ were found to contains either 11 (33), 13 (13), or 15 (12) nucleotides whereas tRNA^Ser^ was found to contain either 11 (2), 14 (33), 17 (1), 18 (6), 19 (14), or 20 (1) nucleotides in the variable region. The presence of variable number of nucleotides in the variable loop region of tRNA^Leu^ and tRNA^Ser^ reflect, these tRNA are much diverse then the other tRNAs and addition of nucleotides might happened in the variable loop region. This also suggests that tRNA^Leu^ and tRNA^Ser^ are also highly prone to the genetic mutation to incorporate the nucleotides in the variable region. Gaus et al. (1979) reported that type I tRNA contain four or five nucleotides in the variable loop, whereas type II tRNA contain more than five nucleotides (Gaus et al., 1979; Tocchini-Valentini et al., 2000). These results suggest that all tRNA^Leu^ and tRNA^Ser^ of *O. sativa* are belonged to the type II tRNA. Only type II tRNA forms the stem-loop structure and these gross structural differences of tRNAs might playing a significant role in recognition of tRNA by minoacyl-tRNA synthateses. tRNA is characterized by its clover leaf like structure that contains a small stem-loop structure in the variable region. However, the majority of *O. sativa* tRNA do not contain the loop in the variable region. Additionally, the variable loop was found to be absent from all tRNAs those were found to contain <11 nucleotides. In our study we found that, only tRNA^Leu^ and tRNA^Ser^ contained a loop in the variable region. There was only a few tRNA^Tyr^ (seven) and tRNA^Val^ (one) those were found to contain loop in the variable region (Supplementary Table 2).

It is well known fact that the Ψ-arm possess only five nucleotides; however, *O. sativa* tRNA were found to contain three to five nucleotides in the Ψ-arm. Although, the majority of tRNA were found to contain five nucleotides in the Ψ-arm, a few tRNA were found to contain either three or four nucleotides. Specifically, tRNA^Gly^ (7), tRNA^Cys^ (2), tRNA^Leu^ (1), tRNA^Lys^ (1), tRNA^Met^ (1), tRNA^Ala^ (1), tRNA^Thr^ (1), and tRNA^Val^ (1) were found to contain only four nucleotides in the Ψ-arm, whereas a tRNA^Met^ was found to encode only three nucleotides. The Ψ-loop of tRNA reportedly contains seven nucleotides. Although, the Ψ-loop of the majority of *O. sativa* tRNA was found to possess seven nucleotides (Supplementary Table 2), a few of them were found to contain more than seven nucleotides. Specifically, tRNA^Gly^36 (chr12), tRNA^Leu^7 (chr2), and tRNA^Val^59 (chr5) were found to contain nine nucleotides, whereas tRNA^Val^45 (chr5) contained 11 nucleotides. The presence of such novel form of tRNA might have some novel function as well, and laboratory based experimental study can reveal the exact function of the presence of additional number of nucleotides in the Ψ-loop.

### *O. sativa* tRNAs are conserved

**Figure 4:**
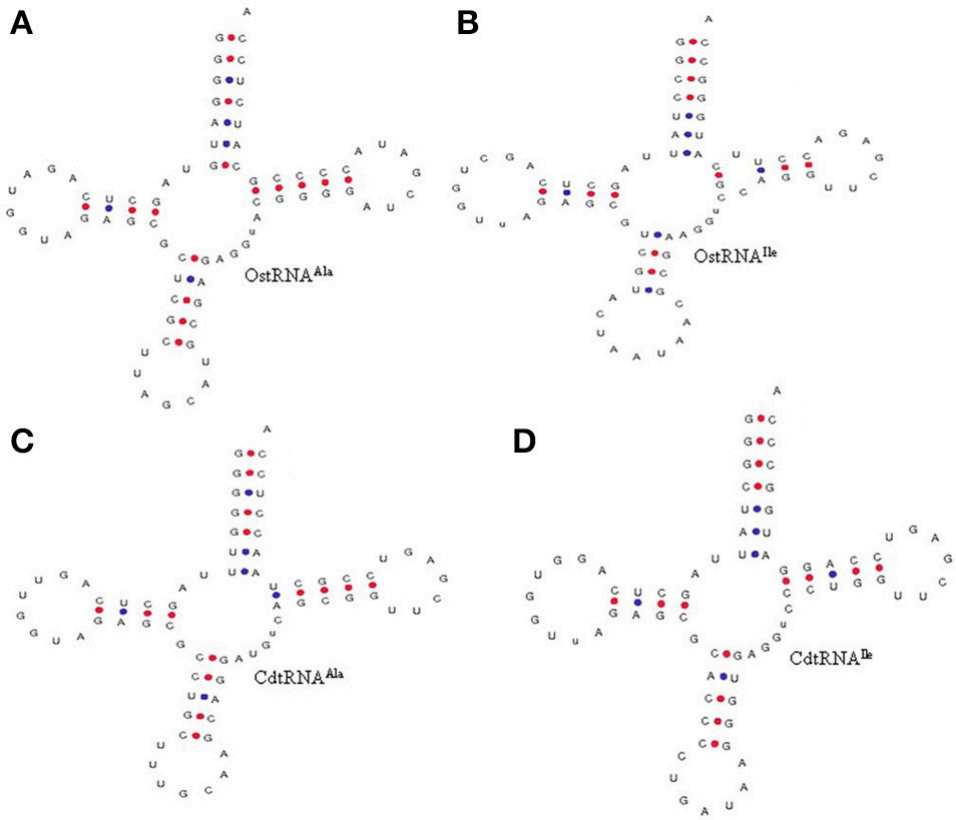
Similarity in the 3′CCA tail of *O. sativa* **(A)** tRNA^Ala^ and **(B)** tRNA^Ile^ with prokaryotic (*Calothrix desertica*) **(C)** tRNA^Ala^ and **(D)** tRNA^Ile^. The tRNA gene sequence in both the organisms contains the 3′CCA tail sequence. Prokaryotic organisms usually lack of nucleotidyl transferase enzyme, which adds the 3′CCA into the tRNA, while eukaryotic organisms contain this enzyme. However, the presence of 3′CCA sequence in the *O. sativa* tRNA^Ala^ and tRNA^Ile^ gene shows that these tRNA might not require undergoing processing for addition of the 3′CCA tail. The presence of a 3′CCA sequence in *O. sativa* tRNA^Ala^ and tRNA^Ile^ indicates that *O. sativa* tRNAs have evolved from common prokaryotic ancestors. CdtRNA; *Calothrix desertica* tRNA, OstRNA; *Oryza sativa* tRNA.

The nucleotide residue at a specific position in the tRNA accounted for the specific tertiary folds and aminoacylation. Beside this, they also dictate to transit different sites in the ribosome. Protein synthesis cannot occur until and unless tRNA gets properly engaged with the ribosome. Therefore, understanding the specific nucleotide position in tRNA is very important and hence we analyzed the tRNA sequences to find their conserved regions. Analyses revealed that the acceptor arm (AM), D-arm (DA), D-loop (DL), anti-codon arm, anti-codon loop, variable region (VR), Ψ-arm, and Ψ-loop were found to contain conserved nucleotides at the specific position (Table 5). Specifically, tRNA^Arg^, tRNA^Asp^, tRNA^Cys^, tRNA^Gly^, tRNA^His^, tRNA^Ile^, tRNA^Leu^, tRNA^Lys^, tRNA^Phe^, tRNA^Ser^, tRNA^Thr^, tRNA^Trp^, and tRNA^Ala^ were found to contain conserved 5′ G nucleotide at the first position in the acceptor arm (Supplementary Figure 1, Table 5). The tRNAs those were found to contain conserved U nucleotide at the first position of the acceptor arm were tRNA^Asn^, tRNA^Glu^, and tRNA^Gln^. A few tRNA^Met^, tRNA^Pro^, and tRNA^Val^ were found to contain A nucleotide at the first position. The D-arm was found to contain a conserved 5′ G nucleotide at the first position, except for tRNA^Tyr^ and tRNA^Pro^. Few of the tRNAs those were found to contain conserved G-C nucleotides at the first and second position were tRNA^Ala^, tRNA^Ile^, tRNA^Cys^, tRNA^Asn^, tRNA^Lys^, and tRNA^Glu^, whereas tRNA^Leu^, tRNA^Met^, and tRNA^Thr^ did not contain any conserved residues in the D-arm. The D-loop was found to be highly conserved and was found to contain an A-G/x-U/x consensus sequence. In the D-loop, nucleotide A was found to be conserved at the first position and it can be called as the identity element of the D-loop (Supplementary Figure 1, Table 5); however, tRNA^Pro^ and tRNA^Val^ were found to contain conserved U instead of A nucleotide at the first position. tRNA^Met^ and tRNA^Ser^ were devoid of any conserved nucleotide in the D-loop. In the majority of tRNAs, the anti-codon arm was found to contain a G nucleotide at the first position. However, tRNA^Val^ was found to contain a U nucleotide at the same position whereas tRNA^Ala^, tRNA^Asp^, and tRNA^Leu^ were found to contain C nucleotide. In the anti-codon loop, a U nucleotide was found to be conserved at the first position in the majority of tRNAs. However, tRNA^Lys^, tRNA^Cys^, tRNA^Tyr^, and tRNA^Glu^ were found to contain a C nucleotide in the first position in the anti-codon loop. The C-A-U-A nucleotides of tRNA^Met^ were found to be conserved from 3rd to 6th position in the anti-codon loop. No conserved site was found for tRNA^Ser^ in the anti-codon loop. The variable loop is considered very dynamic because of its non conserved and variable nucleotide composition. However, the variable loop was also found to be conserved in different tRNA when tRNA was studied as individual gene family (Supplementary Figure 1, Table 5). All tRNA were found to contain conserved nucleotide sequences at different position in the variable region except for tRNA^Gly^, tRNA^Phe^, tRNA^Gln^, and tRNA^Asp^ (Supplementary Figure 1, Table 5). In the majority of cases, the G nucleotide was found to be conserved at different positions in different tRNA (Supplementary Figure 1, Table 5). No conserved nucleotides were found in the Ψ-arm of tRNA^Pro^. The Ψ-loop was found to contain conserved U-U-C-x-A nucleotides in most of the tRNA. These nucleotides were conserved at the first, second, third and fifth position of the Ψ-loop. However, tRNA^Ala^, tRNA^Met^, and tRNA^His^ were found to contain a conserved U-C-x-A consensus sequence and the pseudouridine residue was found to be conserved at the second position of the Ψ-loop (Table 5). Except for tRNA^Ser^, the opposite side of the Ψ-arm that present immediately after the Ψ-loop was found to contain a conserved C nucleotide at the first position. The nucleotide sequences of the acceptor arm were conserved at different positions. These conserved residues were resulted when the tRNA sequences were grouped according to their individual gene family. When all tRNA sequences were aligned, we did not find significant conserved residues, which was concordant with previously reported results. This might have been due to the engagement of different tRNA with different cognate enzymes during the event of protein synthesis. It was also a common concept that all tRNA contain conserved G nucleotides at the first position in the acceptor arm. However, the majority of tRNAs, but not all, were found to contain a G nucleotide at the first position (Supplementary Figure 1). The tRNAs those lacked conserved G nucleotide in the 5′ position were tRNA^Asn^, tRNA^Gln^, tRNA^Glu^, tRNA^Met^, tRNA^Pro^, tRNA^Tyr^, and tRNA^Val^ (Supplementary Figure 1). The 3′CCA sequence from 74 to 76 nucleotides is added to all tRNA during the processing of tRNA. The 3′CCA sequence plays an important role in amino acid attachment sites for tRNA to participate to the protein synthesis. It is also play important role in transport of tRNA from the nucleus to the cytoplasm and acts as a checkpoint for tRNA maturity. The 3′CCA sequence is synthesized by a CCA-adding enzyme popularly known as tRNA nucleotidyl transferase (Shi et al., 1998; Vortler and Morl, 2010; Betat and Mörl, 2015). The attachment of CCA does not occur in few prokaryotes because they already contain the CCA tail in their genetic architecture and a few of them lack CCA tail adding enzyme. However, for species that does not have CCA tail, it adds the tail by CCA tail adding enzyme (Hou, 2010). A few species of archaea contain class I whereas bacteria and eukaryote have class II nucleotidyl transferase CCA tail adding enzyme (Yue et al., 1996). In *Bacillus subtilis*, the CCA tail adding enzyme adds the tail under certain kinetic conditions similar to *in-vivo*. However, this CCA tail adding happens under certain stress and DNA damage conditions and cannot imply direct implementation of the concepts to cells (Hou, 2010). In *O. sativa* tRNA^Ala^ and tRNA^Ile^ already contain the 3′CCA sequence in their tRNA gene (Supplementary Figure 1). When we compared a tRNA^Ala^ and tRNA^Ile^ of *O. sativa* with prokaryotic tRNA^Ala^ and tRNA^Ile^ in the tRNA scan server, we found a similar structure in both the cases (Figure 4). In prokaryotes, the enzyme that adds the 3′ CCA tail to the tRNA is absent; hence, they directly encode the 3′CCA sequence in the tRNA gene. However, unlike prokaryotes, plants encode enzymes that add a 3′CCA tail and still contain a CCA sequence in tRNA genes. These findings indicate that, although tRNA undergoes processing to add the 3′CCA tail, a few tRNA already possess it. However, most of the tRNA does not have nucleotides for 3′CCA tail. tRNA^Ala^ and tRNA^Ile^ are classic examples of this novel phenomenon and contain embedded 3′CCA nucleotide sequence. It is also possible that, *O. sativa* tRNA^Ala^ and tRNA^Ile^ are the primitive forms of tRNA that evolved from their common ancestor of prokaryotic lineages and later diversified.

**Table 5:** Conserved nucleotides of *O. sativa* tRNAs.

**tRNA**	**AC arm**	**D-arm**	**D-loop**	**ANC- arm**	**ANC loop**	**Variable region**	**Ψ-arm**	**Ψ-loop**
Glycine (Gly)	G	G	A-G-x-G-G-U-x-A	G	U-x-U-G	^****^	C-x_3−4_-G	U-U-C-x-A-A-U
Alanine (Ala)	G	G-C	A-x_3_-G	C-x_2_-G	G-C-A	G-G	G-G-x-G	U-C-G-A
Proline (Pro)	G-G-G-x_3_-U	C	U-G-G-U-A	G-x-U	U-x-G-G-G-U	A-x-G-U-C	^****^	U-U-C-x-A-x-U
Valine (Val)	G	G	U-x-G-x_2_-U-x-U	U	U-x-A-C	G-U-C	C-x-G	U-U-C-G-A
Leucine (Leu)	G	^****^	A-x-U-x-G-G-U	C	U-x-A	U-x-C-x_4−8_-G	G	U-U-C-x-A-x-U
Isoleucine (Ile)	G-G-x-C	G-C-U-C	A-G-x-U-G-G-U-U-A	G	U-x-A-U-x-A	A-G-G-U-C	G-C-x-G-G	U-U-C-G-A-G
Methionine (Met)	^****^	^****^	^****^	^****^	C-A-U-A	U	G	U-C-x-A
Phenylalanine (Phe)	G-x_4_-G-A	G	A-x-U-x-G	G-A	C-U-G-A-A-x-A	^****^	C-x_2_-G	U-U-C-x-A
Tyrosine (Tyr)	G	C	A-G-x_2_-G-x_2_-A	G-A	C-U-x-U-A-x-A	G-x_2−12_-C	G-C-x-G-G	U-U-C-x-A-A-U
Tryptophan (Trp)	G-x_4_-C	G-x_2_-C	A-x-U-x_0−2_-G-G-U-A	G-x-C	U-C-C-A-x-A	A-x-G-U	G	U-U-C-x-A-x-U
Serine (Ser)	U	G	^****^*	U	^****^	C	G	U-U-x-G-A-x-U-C
Threonine (Thr)	G-C	^****^	G-x_2_-G-x_3_-A	^****^	U-x-G-U-A-A	G-U-C	G	U-U-C-x-A
Cysteine (Cys)	G	G-C	A-G-x_2_-G-x_1−2_-U-A	G	C-U-x-C-A-x-A	U-C	G	U-U-C-x-A
Asparagine (Asn)	C	G-C-U	A-G-U-x-G-U	G	U-G-U-U	G-G-U	G-x_2_-G-G	U-U-C-x-A
Glutamine (Gln)	G	G	A-G-x-G-G-U-x-A	G	U-x-U-G	^****^	C-x_3−4_-G	U-U-C-x-A-A-U
Lysine (Lys)	G	G-C-x-C	A-G-x_2_-G-G-x-A	G-G	C-U-x-U-U-A	U-C	G-x_2_-G-G	U-U-x_2_-A
Histidine (His)	G-G	G	A-G-U-x-G	G	U-U-G-U-G	A-x_2_-C	G-G-G	U-C-x-A-x-U
Arginine (Arg)	G	G	A-x_2_-G-G-A-U-x-A	G	U-x-C-x_2−3_-A	U	x-x-G-G	U-U-C-x-A
Aspartate (Asp)	G	G	A-x-U-x-G-G-U-x-A	C	U-x-U-C-A	^****^	C-G-G-G	U-U-C-G-A-x-C
Glutamate (Glu)	C-C-x_2_-U	U-C-U	A-x_3−8_-G-x-U	^****^	C-U-x-U-C-A	C	G-G	U-U-C-x-A-x-U

*Multiple sequence alignment of individual tRNA family shows highly conserved region in different parts of the tRNA. The majority of tRNAs contain consensus nucleotide sequence in D-loop and Ψ-loop. The nucleotide sequences of tRNA^Met^ and tRNA^Ser^ are highly diverse and only conserved in few positions. Analyses shows, the variable region of tRNA also conserved in individual group of tRNA. However, there are lots of isodecoders in the O. sativa tRNA. Multiple sequence alignment was conducted using Multalin server. AC, Acceptor, ANC, anti-codon*.

### *O. sativa* encodes novel/pseudo tRNA

**Figure 5:**
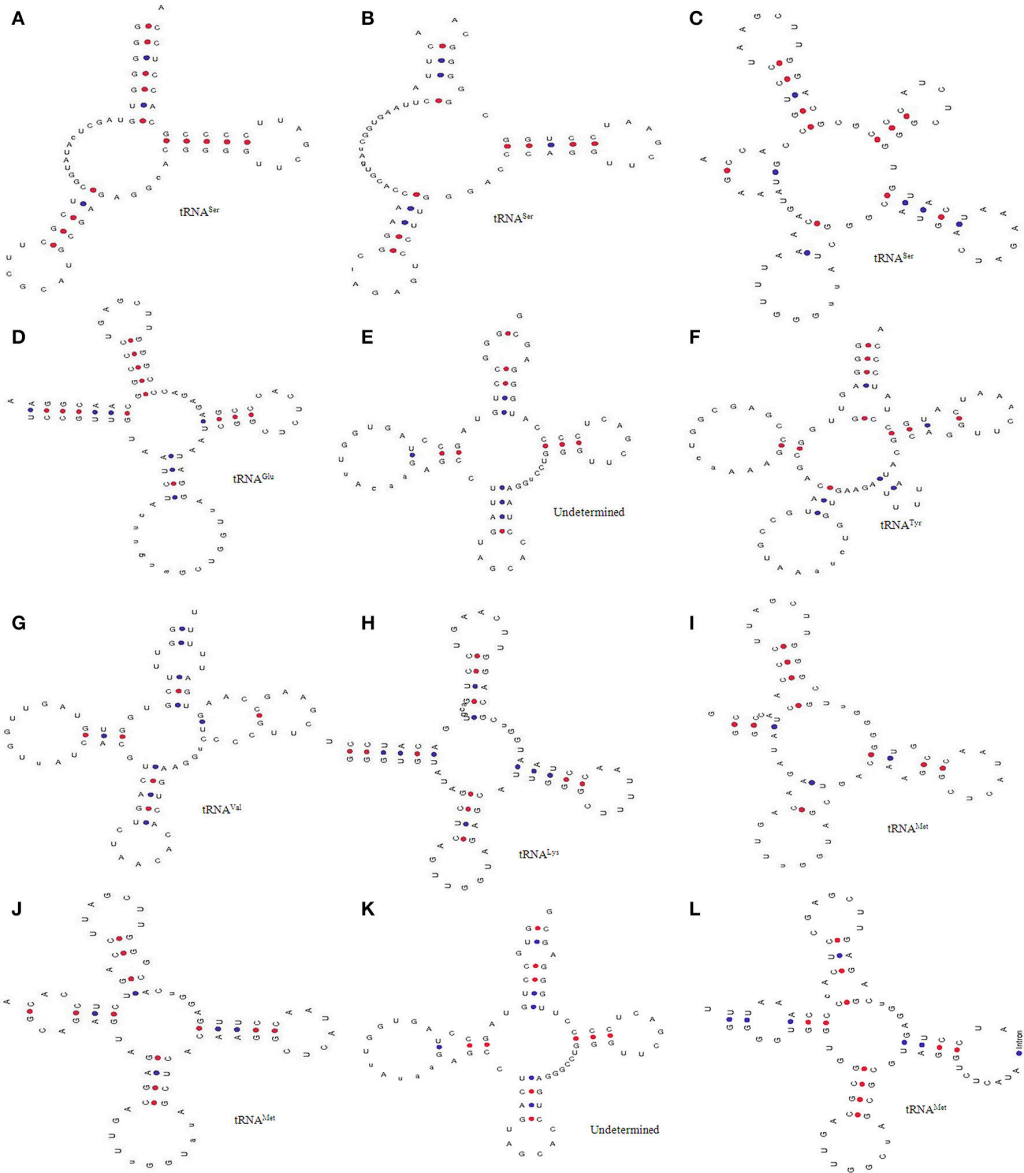
Novel tRNAs of *O. sativa*. Analysis through tRNA scan revealed the presence of 14 novel forms of tRNA (denoted type A to L). **(A)** D arm and D-loop was found to be absent, **(B)** D arm was found to be absent and it contained 17 nucleotides in between the acceptor arm and the anti-codon arm, **(C)** presence of loop like acceptor arm, **(D)** D-loop contained 15 nucleotides with modified bases, **(E)** bulged acceptor and D-arm, **(F)** bulged acceptor arm and the D-arm containd only two nucleotides **(G)** bulged acceptor arm and only three nucleotides in the D-arm, **(H)** one side of Ψ-arm contained five nucleotides whereas other side contianed eight, **(I)** shorter acceptor arm having three nucleotides in one side whereas six in other side, **(J)** bulged acceptor and Ψ-arm **(K)** bulged acceptor arm and D-arm, and **(L)** bulged acceptor arm and only four nucleotide in the anti-codon arm. These novel tRNA are might be responsible for novel functional roles in *O. sativa*.

Modification of nucleotide bases in tRNA are seen as an integral part of its novelty. We found several modifications in the tRNA of *O. sativa* and named them accordingly. Type I tRNA was found to contain seven nucleotides in the acceptor arm (Figure 5A). Additionally, the D-arm and the D-loop were found to be absent from type I tRNA (tRNA^Ser^1, chr.8) and was found to contain 12 nucleotides in between the acceptor arm and the anti-codon arm. The anti-codon arm and the anti-codon loop were found to contain five and seven nucleotides, respectively while the variable region was found to contain five nucleotides. The Ψ-arm and the Ψ-loop were found to contain five and seven nucleotides, respectively. Type II tRNA was found to possess six nucleotides in the acceptor arm (Figure 5B). The D-arm and the D-loop were found to be absent from type II tRNA, whereas it was found to contain 17 nucleotides in between the acceptor arm and the anti-codon arm (tRNA^Ser^27, chr.3) (Figure 5B). The anti-codon loop of type II tRNA was found to contain a modified inosine base at the second position. In type III tRNA, the acceptor arm was almost absent and instead it was found to contain a hairpin loop-like bulge structure (tRNA^Ser^20, chr.2) of having four nucleotides (Figure 5C). There was presence of only four nucleotides from the acceptor arm to the D-arm. The D-arm was found to contain a loop-like bulge having only three nucleotides. The D-loop was found to contain 10 nucleotides with the presence of pseudouridine bases at 8th and 9th position. The anti-codon arm, the anti-codon loop, the Ψ-arm, and the Ψ-loop of type III tRNA were found to be similar to that of the canonical tRNA. Type IV tRNA (tRNA^Glu^11, chr.7) was found to contain the canonical tRNA form, except for the D-loop (Figure 5D). The D-loop of type IV tRNA was found to contain 15 nucleotides with most probably modified bases. The type V tRNA (tRNA^Undet^16, chr.2) was found to contain a hairpin loop-like bulge in the acceptor arm, and the D-arm where the D-loop was found to contain 11 nucleotides (Figure 5E). The anti-codon arm was found to contain only four nucleotides whereas the anti-codon loop was found to contain only six nucleotides which was one nucleotide lesser than the usual canonical tRNA. The type VI (tRNA^Tyr^49, chr.8) tRNA was found to contain a hairpin loop-like bulge structure in the acceptor arm and was found to contain only two nucleotides in the D-arm (Figure 5F). The D-loop was found to contain 13 nucleotides whereas the anti-codon arm was found to contain only three nucleotides. The anti-codon loop was found to contain 12 nucleotides which was much higher than the canonical tRNA. Type VII (tRNA^Val^30, chr.9) tRNA was found to contain a hairpin loop-like bulge in the acceptor arm and three nucleotides in the D-arm (Figure 5G). The D-loop of type VII tRNA was found to contain 11 nucleotides, whereas the anti-codon arm and the anti-codon loop were found to contain five and seven nucleotides, respectively. The Ψ-arm was found to contain two hairpin loop-like structures attached to the Ψ-loop. Type VIII (tRNA^Lys^18, chr.10) tRNA showed similarities with the usual canonical tRNA, except for the Ψ-arm (Figure 5H). Here, one side of the Ψ-arm was found to contain five nucleotides, whereas the other side contained eight nucleotides. Type IX (tRNA^Met^22, chr.10) tRNA was found to contain a shorter acceptor arm having three nucleotides on one side and six on the other side (Figure 5I). With the exception of the acceptor arm, the other three arms of tRNA were found to contain a hairpin loop-like bulge (Figure 5I). The shorter acceptor arm contained only three nucleotides on one side and six nucleotides on the other side. The D-arm was found to contain only three nucleotides and forms a hairpin loop-like structure. Similarly, the anti-codon arm and Ψ-arm was found to contain a hairpin loop-like structure. Type X (tRNA^Met^21, chr.12) tRNA was found to contain a hairpin loop-like bulge in the acceptor arm and the Ψ-arm, whereas other parts of the tRNA was found to be similar to the canonical tRNA (Figure 5J). Type XI (tRNA^Undet^46, chr.10) tRNA was found to contain a hairpin loop-like bulge in the acceptor arm and D-arm (Figure 5K). The anti-codon arm contained only four nucleotides and the anti-codon loop contained only six nucleotides. The Ψ-arm was found to contain only three nucleotides instead of five nucleotides. Type XII (tRNA^Met^55, chr.1) tRNA was found to contain a hairpin loop-like bulge in the acceptor arm and only four nucleotides in the anti-codon arm (Figure 5L). The anti-codon loop contains introns and the Ψ- arm contained a hairpin loop-like bulged structure.

Different organisms have evolved different method for processing and maturation of tRNA at every step; therefore, some are might be utilizing novel mechanisms. This study revealed the presence of multiple novel forms of tRNA in *O. sativa*. These novel types of tRNA might have some novel function and processing pathways which are yet to be elucidated. Indeed, pseudo tRNA was found to mediate antibiotic resistance in *Bacillus cereus* (Rogers et al., 2012); therefore, the role of annotated pseudo tRNA cannot be underestimated and additional study is required to elucidate their exact function and mechanisms.

### *O. sativa* tRNA were evolved from their multiple common ancestors

**Figure 6:**
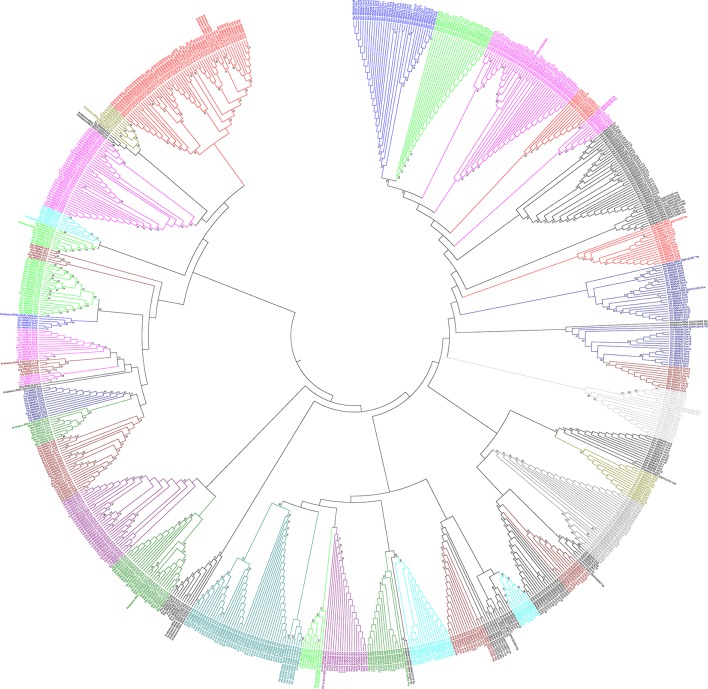
Phylogenetic tree of *O. sativa* tRNAs was constructed using the tRNA genes of *O. sativa*. tRNA of *A. cylindrica* was used as probable prokaryotic common ancestor. Phylogenetic tree was constructed used using MEGA6 software.

**Figure 7:**
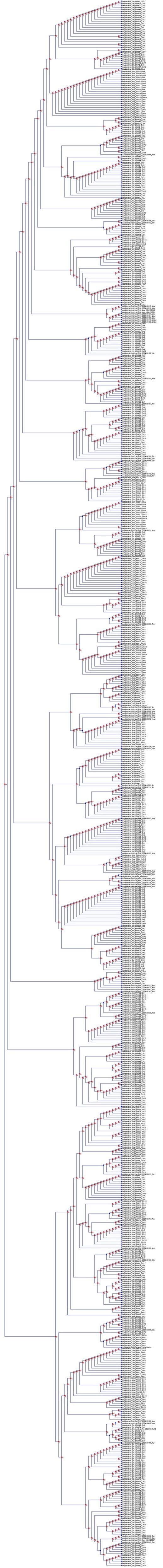
Duplication of *O. sativa* tRNAs study revealed the presence of 778 duplicated nodes. Duplication study was conducted using Notung 2.6 software.

**Figure 8:**
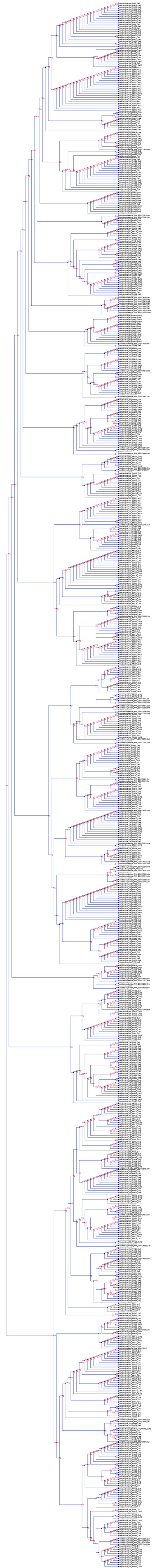
Loss of *O. sativa* tRNAs study revealed 17 *O. sativa* tRNA were lost the during evolution. tRNA loss was conducted using Notung 2.6 software.

A phylogenetic tree was constructed using the maximum likelihood method to infer the evolutionary relationship of *O. sativa* tRNA (Figure 6). The tRNAs of the prokaryotic organism *Anabaena cylindrica* was used as a probable common ancestor of *O. sativa* tRNA. The phylogenetic tree showed the presence of at least 44 different groups from 20 different types of tRNA (Figure 6). Some tRNA were found to form one group, while a few were found to form more than one group in the phylogenetic tree at different phylogenetic distances. The tRNAs those were found to form only one group in the phylogenetic tree were tRNA^Phe^, tRNA^Ala^, and tRNA^Ser^ whereas those clustered into two groups were tRNA^Glu^, tRNA^Asp^, tRNA^Gly^, tRNA^His^, tRNA^Pro^, tRNA^Thr^, tRNA^Asn^, tRNA^Tyr^, tRNA^Lys^, tRNA^Trp^, tRNA^Cys^, tRNA^Ile^, tRNA^Val^, and tRNA^Gln^ (Figure 6). Moreover, tRNA^Leu^ and tRNA^Met^ were clustered into four groups whereas tRNA^Arg^ was clustered into five groups. Multiple grouping of different tRNA in the phylogenetic tree reflected their evolution from multiple common ancestors. At least 16 tRNA families of *O. sativa* evolved were from their common ancestors of two or more lineages. tRNA^Phe^, tRNA^Ala^, and tRNA^Ser^ of *O. sativa* were most probably descended from a single lineage, whereas tRNA^Met^ might have descended from at least four and tRNA^Arg^might have descended from five lineages. To support the evolution of *O. sativa* tRNA from multi lineages, we studied the orthology and paralogy relationship. We found that all of the *O. sativa* tRNA are paralogous to each other. *O. sativa* was found to contain only twelve orthologous tRNA which were restricted to tRNA^Ser^ only. These orthologous tRNAs were tRNA^Ser^15 (chr2), tRNA^Ser^26 (chr2), tRNA^Ser^31 (chr2), tRNA^Ser^6 (chr4), tRNA^Ser^5 (chr4), tRNA^Ser^83 (chr4), tRNA^Ser^89 (chr4), tRNA^Ser^10 (chr10), tRNA^Ser^28 (chr10), tRNA^Ser^36 (chr10), tRNA^Ser^62 (chr10), and tRNA^Ser^25 (ch11). The absence of orthologous genes further reflected their evolution from multiple common ancestors. Similarly, an orthology study with tRNA of *A. cylindrica* showed orthologous evolution of 15 *O. sativa* tRNA which included tRNA^Phe^, tRNA^Asn^, tRNA^Trp^, tRNA^Lys^, tRNA^Arg^, tRNA^Ile^, tRNA^Asp^, tRNA^Pro^, tRNA^Met^, tRNA^Glu^, tRNA^Gln^, tRNA^Tyr^, tRNA^Ser^, tRNA^His^, and tRNA^Gly^. tRNA tRNA^Ala^, tRNA^Thr^, tRNA^Val^, tRNA^Arg^, and tRNA^Leu^ of *O. sativa* had no orthologous ancestor tRNA in common with *A. cylindrica*. However, a few modified orthologous tRNA of *A. cylindrica* were present in the *O. sativa*. The orthologous tRNA^Phe^, tRNA^Thr^, tRNA^Arg^ÃĆÂý tRNA^Asp^, tRNA^Thr^, tRNA^Trp^, tRNA^Tyr^, and tRNA^Met^ of *A. cylindrica* were found to be modified and present as substitute where they were substituted as follows: tRNA^Phe^ > tRNA^Thr^, tRNA^Thr^ > tRNA^Met^, tRNA^Arg^ > tRNA^Met^, tRNA^Asp^ > tRNA^Val^, tRNA^Tyr^ > tRNA^Leu^, tRNA^Met^> tRNA^Gly^. These finding indicated that several ancestral orthologous tRNA of one form evolved and have modified to another form, which might have occurred in response to several instances of evolutionary pressure. We find these novel modification of *O. sativa* tRNA and this might be due to fact that, as tRNAs are conserved in different regions, but they are significantly varied in the anti-codon region. This modification makes them as distinct tRNA. However, modification of one form of orthologous tRNA (e.g., modification of *A. cylindrica* tRNA^Phe^ to tRNA^Thr^ or tRNA^Thr^ to tRNA^Met^, etc.) of one species to another form in different species is of particular interest. Therefore, we studied gene duplication and loss event of *O. sativa* and *A. cylindrica* tRNA. After reconciliation of *O. sativa* and *A. cylindrica* species trees we found that 778 genes were duplicated (Figure 7) whereas only 50 genes were found to be lost (Figure 8). There was no conditional duplication in *O. sativa* tRNA. Only a few tRNAs of *O.sativa* were found to be lost during the evolution process, suggesting the duplication event has favored the evolution of *O. sativa* tRNA. Overall, we found that at least 17 *O. sativa* and 34 *A. cylindrica* tRNA were lost during the evolution.

### Transition/transversion of *O. sativa* tRNA

In 1962 Zuckerkandl and Pauling proposed molecular clock hypothesis which indicated the rate of molecular evolution is approximately constant over time for all proteins in all lineages (Zuckerkandl and Pauling, 1962). Based on this theory, any time of divergence between proteins, genes or lineages can be dated by measuring the number of changes between the sequences. Later, Holmquist, Jukes, and Cantor proposed a stochastic model for substitution of DNA in which the nucleotide substitution occurs at an equal rate, and whenever a nucleotide is substituted, any one of the other nucleotides replaces the substitution (Holmquist et al., 1972). Substitutions that change a purine nucleotide to another purine (A to G or G to A) or pyrimidine to another pyrimidine (C to U/T or U/T to C) are called transitions whereas those changes a purine nucleotide to a pyrimidine (A or G to U/T or C) or vice versa (U/T or C to A or G) are called transversions. The rate of mutation/substitution varies among and within the genome and affected by several factors including nearest neighbor bases, chromosomal position, and efficiency of repair system between the leading and lagging DNA strands. During the repair process, the replacement of a base is facilitated by a similar one or a derivative of a similar one. As a result, transition occurs more persistently (sometimes twice as frequently) than transversion. The differences in substitution rates tend to decrease TA and CG dimers and produce excess CT and TG dimers. Substitution analysis of *O. sativa* tRNA revealed, the rate of transition from A to G was found to be highest for tRNA^Asp^ (62.18) and lowest for tRNA^Asn^ (0.31) (Table 6). Similarly, the transition from G to A was found to be highest in tRNA^Asp^ (27.52) and lowest in tRNA^Asn^ (0.18). The rate of transition from U to C was found to be highest in tRNA^Gln^ (36.44) and lowest in tRNA^Thr^ (6.25). Similarly, the transition from C to U was found to be highest for tRNA^Gln^ (40.1) and lowest for tRNA^Thr^ (7.2). Based on these findings, it was clear that the substitution rate of A to G was highest and that of G to A was lowest (Table 6). Unlike transition, the transversion rate from A to C/U or vice versa was found to be maximum for tRNA^Tyr^ (6.02) and minimum for tRNA^Asp^ (0.62). The transversion rate from U to A/G or vice versa was maximum for tRNA^Tyr^ (7.24) and minimum for tRNA^Asp^ (0.93). The transversion rate from C to A/G or vice versa was maximum for tRNA^*Gly*^ and minimum for tRNA^Asp^. The transversion from G to U/C or vice versa was found to be maximum for tRNA^Ile^ (8.35) and minimum for tRNA^Asp^ (1.39). Overall, the result clearly indicated that the highest or lowest transition/transversion rate was concentrated to only few tRNA. The maximum and minimum transition/transversion rate in either of the nucleotide was mostly concentrated in tRNA^Asp^. The tRNA those have the highest and lowest transition/transversion rates were tRNA^Asp^, tRNA^Thr^, tRNA^Gly^, tRNA^His^, tRNA^Asn^, tRNA^Ile^, and tRNA^Gln^. All of them belonged to two evolutionary groups in the phylogenetic tree. These finding indicated that tRNA are more prone to substitution for evolution of novel tRNA, which might explain why several orthologous tRNA of *A. cylindrica* have modified orthologous tRNA in *O. sativa*. When we studied the transition/transversion bias, we found it was highest in tRNA^Asp^ (*R* = 9.473) and lowest in tRNA^Tyr^ (*R* = 0.684) (Table 7). When *R* = 0, there is no bias toward either transition/transversion where bias *R* = [A^*^G^*^k1 + T^*^C^*^k2]/[(A+G)^*^(T+C)] and *k1* and *k2* indicating the purine and pyrimidine, respectively. The transition/transversion bias is highly correlated with the highest and lowest substitution of tRNA^Asp^. As all pairwise distances in a distance matrix are correlated because of phylogenetic relationships among the sequences, the sum of their log-likelihoods is a composite likelihood. The likelihood ratio observed on the present study suggests that the transition/transversion ratio might be variable among evolutionary lineages; however, an earlier report suggests that the rate of transition in DNA is higher than the rate of transversion. Our study in *O. sativa* tRNA also showed that the rate of transition is higher than the rate of transversion.

**Table 6:** Nucleotide substitution matrix of *O. sativa* tRNAs.

	**A**	**U**	**C**	**G**
**GLYCINE**				
A	–	*5.77*	*7.49*	**11.99**
U	*5.52*	–	**14.98**	*7.61*
C	*5.52*	**11.54**	–	*7.61*
G	**8.7**	*5.77*	*7.49*	–
**SERINE**				
A	–	*5.44*	*4.88*	**7.35**
U	*4.09*	–	**21.9**	*6.39*
C	*4.09*	**24.44**	–	*6.39*
G	**4.7**	*5.44*	*4.88*	–
**THREONINE**				
A	–	*2.02*	*1.76*	**39.1**
U	*1.79*	–	**6.25**	*2.2*
C	*1.79*	**7.2**	–	*2.2*
G	**31.91**	2.02	*1.76*	–
**CYSTEINE**				
A	–	*5.05*	*6.3*	**20.35**
U	*5.01*	–	**10.2**	*7.35*
C	*5.01*	**8.17**	–	*7.35*
G	**13.87**	*5.05*	*6.3*	–
**TYROSINE**				
A	–	*7.24*	*6.81*	**14.09**
U	*6.02*	–	**8.71**	*9.37*
C	*6.02*	**9.27**	–	*9.37*
G	**9.05**	*7.24*	*6.81*	–
**ASPARAGINE**				
A	–	*3.74*	*3.07*	**0.31**
U	*2.71*	–	**32.27**	*4.49*
C	*2.71*	**39.22**	–	*4.49*
G	**0.18**	*3.74*	*3.07*	–
**GLUTAMINE**				
A	–	*2.65*	*2.41*	**2.24**
U	*1.9*	–	**36.44**	*2.92*
C	*1.9*	**40.1**	–	*2.92*
G	**1.46**	*2.65*	*2.41*	–
**ALANINE**				
A	–	*3.28*	*4.37*	**22.42**
U	*3.19*	–	**18.24**	*5.58*
C	*3.19*	**13.66**	–	*5.58*
G	**12.85**	*3.28*	*4.37*	–
**VALINE**				
A	–	*4.92*	*4.62*	**9.87**
U	*4.11*	–	**21.51**	5.57
C	*4.11*	**22.91**	–	5.57
G	**7.27**	*4.92*	*4.62*	–
**ASPARTATE**				
A	–	*0.93*	*1.14*	**62.18**
U	*0.62*	–	**1.18**	*1.39*
C	*0.62*	**0.97**	–	*1.39*
G	**27.52**	*0.93*	*1.14*	–
**LEUCINE**				
A	–	*4.83*	*4.8*	**18.44**
U	*5*	–	**12.54**	*6.65*
C	*5*	**12.61**	–	*6.65*
G	**13.86**	*4.83*	*4.8*	–
**ISOLEUCINE**				
A	–	*5.48*	*6.28*	**9.86**
U	*5.09*	–	**18**	*8.35*
C	*5.09*	**15.71**	–	*8.35*
G	**6.02**	*5.48*	*6.28*	–
**PROLINE**				
A	–	*4.73*	*3.51*	**24.99**
U	*2.79*	–	**12.4**	*5.11*
C	*2.79*	**16.67**	–	*5.11*
G	**13.66**	*4.73*	*3.51*	–
**PHENYLALANINE**				
A	–	*3.35*	*3.81*	**0.8**
U	*3.73*	–	**35.43**	*5.13*
C	*3.73*	**31.14**	–	*5.13*
G	**0.58**	*3.35*	*3.81*	–
**TRYPTOPHAN**				
A	–	*3.7*	*3.54*	**23.19**
U	*2.91*	–	**16.02**	*4.69*
C	*2.91*	**16.75**	–	*4.69*
G	**14.37**	*3.7*	*3.54*	–
**METHIONINE**				
A	–	*6.34*	*6.96*	**6.4**
U	*6.27*	–	**17.51**	*8.01*
C	*6.27*	**15.95**	–	*8.01*
G	**5.01**	*6.34*	*6.96*	–
**LYSINE**				
A	–	*2.43*	*3.19*	**35.85**
U	*1.66*	–	**15.14**	*3.94*
C	*1.66*	**11.53**	–	*3.94*
G	**15.06**	*2.43*	*3.19*	–
**ARGININE**				
A	–	*2.82*	*3.39*	**16.71**
U	*2.6*	–	**25.62**	*4.22*
C	*2.6*	**21.29**	–	*4.22*
G	**10.32**	*2.82*	*3.39*	–
**HISTIDINE**				
A	–	*4.76*	*4.95*	**13.37**
U	*3.59*	–	**20.41**	*5.96*
C	*3.59*	**19.64**	–	*5.96*
G	**8.05**	*4.76*	*4.95*	–
**GLUTAMATE**				
A	–	*3.12*	*3.88*	**21.9**
U	*2.6*	–	**20.24**	*3.9*
C	*2.6*	**16.26**	–	*3.9*
G	**14.6**	*3.12*	*3.88*	–

*Transition/transversion rate shows, the transition is more prevalent than the transversion. Higher transition rate was observed in the transition from A to G in and from C to U tRNA^Asp^. Rates of different transitional substitutions are shown in bold and those of transversional substitutions are shown in italics. All positions with <95% site coverage were eliminated. That is, fewer than 5% alignment gaps, missing data, and ambiguous bases were allowed at any position. Evolutionary analyses were conducted in MEGA6*.

**Table 7:** Transition/transversion bias of *O. sativa* tRNAs.

**tRNA**	**k1 (Purines)**	**k2 (Pyrimidines)**	**R (Transition/transversion bias)**	**No. of sequences studied**
Glycine	1.575	2.001	0.877	47
Serine	1.15	4.492	1.38	62
Threonine	17.783	3.556	5.483	39
Cysteine	2.77	1.618	1.094	16
Tyrosine	1.503	1.28	0.684	22
Asparagine	0.068	10.498	2.476	30
Glutamine	0.769	15.118	4.139	31
Alanine	4.021	4.171	1.958	43
Valine	1.771	4.653	1.585	49
Leucine	2.773	2.614	1.362	58
Isoleucine	1.181	2.867	0.942	23
Proline	4.892	3.527	1.972	37
Phenylalanine	0.155	9.291	1.917	19
Tryptophan	4.94	4.53	2.303	18
Methionine	0.799	2.517	0.796	60
Lysine	9.069	4.751	3.059	31
Arginine	3.964	7.551	2.732	59
Histidine	2.242	4.126	4.565	26
Aspartate	44.743	1.034	9.473	32
Glutamate	5.613	5.218	2.63	40

The probability of substitution (r) was analyzed from one base (row) to another base (column). The overall transition/transversion bias R was calculated as follows: R = [A^*^G^*^k_1_ + T^*^C^*^k_2_]/[(A+G)^*^(T+C)]. All positions with <95% site coverage were eliminated. That is, fewer than 5% alignment gaps, missing data, and ambiguous bases were allowed at any position. Evolutionary analyses were conducted in MEGA6 using Tamura–Nei model.

## Materials and methods

*O. sativa* tRNA were downloaded from the genomic tRNA database GtRNAdb (http://gtrnadb.ucsc.edu/). This species was selected for study because it is one of the most studied model crop plant and has a well annotated genome and tRNA. Downloaded tRNA sequences were scanned in stable version tRNAscan-SE 1.23 and 1.3 to check for the presence of a clover leaf structure (Lowe and Eddy, 1997). Default parameters were used to run the sequences in tRNAscan-SE 1.23 and 1.3.

### Multiple sequence alignment

We conducted multiple sequence alignment of all tRNA family individually and collectively as well to identify the shared and conserved sequence homology. Multalin server was used to conduct the multiple sequence alignment (http://multalin.toulouse.inra.fr/multalin/). The statistical parameters used to run the multiple sequence alignments were described previously (Mohanta et al., 2014, 2015b; Mohanta and Bae, 2015), with minor modification. In short they are as follows: sequence input format, fasta; nucleotide weight matrix. Blosum62-12-2; gap penalty at opening, default; gap penalty at extension, default; gap penalties at extremities, none; one iteration only, no; high consensus value, 90% and low consensus value, 50%.

### Construction of phylogenetic tree

A phylogenetic tree infers the evolutionary relationship between different biological species or sequences. Therefore, we constructed a phylogenetic tree of *O. sativa* tRNA by considering the tRNA of *A. cylindrica* as a probable common ancestor. Therefore, the tRNA of *O. sativa* and *A. cylindrica* was subjected to clustal omega server to generate clustal files for all individual tRNA, as well separately to understand their evolution independently. The generated clustal files were later converted to MEGA file format using the MEGA6 software (Tamura et al., 2013). The phylogenetic tree was constructed with minor modification as described previously (Mohanta et al., 2015a, 2017; Mohanta T. et al., 2015; Mohanta and Bae, 2017) with the following statistical parameters: analysis, phylogenetic reconstruction; statistical method, maximum likelihood; test of phylogeny, bootstrap method; number of bootstrap replications, 1,000; substitutions type, nucleotides; model/method, Jukes-Cantor model; rates among sites, uniform rates; gaps/missing data treatment, partial deletion; site coverage cutoff (%), 95; ML heuristic methods, nearest-neighbor-interchange (NNI); initial tree for ML, default-NJ/BioNJ; and branch swap filter, very strong. The phylogenetic tree that contained all of the tRNA of *O. sativa* and *A. cylindrica* was saved as the “gene tree.”

### tRNA duplication/loss

Gene duplication or loss, which is one of the most important events that occurs in a genome leads to evolution of the genome and new genomic content. Duplication/loss events of tRNA were studied using the Notung 2.6 software. A species tree of *O. sativa* and *A. cylindrica* was generated using the NCBI taxonomy browser (http://www.ncbi.nlm.nih.gov/Taxonomy/CommonTree/wwwcmt.cgi). The generated “gene tree” and species tree was analyzed by Notung 2.6 software. During the analysis, the gene tree was reconciled with the species tree to get the duplication/loss events of *O. sativa* and *A. cylindrica* tRNA.

### Analysis of transition/transversion

Transition/transversion explains the substitution rates of nucleotides; therefore, we studied the transition/transversion of all 20 tRNA. The generated MEGA files of all of the tRNA genes were analyzed with the MEGA6 software to find the transition/transversion rate. The statistical parameters used to analyze the transition/transversion rate were as follows: analysis, substitution pattern estimation (MCL); scope, all selected taxa; statistical method, maximum likelihood; substitution type, nucleotides; model/method, Tamura-Nei (automatic selection); gaps/missing data treatment, partial deletion, and site coverage cut off (%), 95.

### Analysis of transition/transversion bias

Some mutations occur more frequently than others; accordingly it is important to understand these biases to interpret the differences that have occurred between different tRNA of the same species. Because the tRNAs are very important molecules involved in protein translation machinery, understanding their transition/transversion is of particular importance. Therefore, we conducted analysis of transition/transversion bias with all tRNA using the MEGA6 software. The generated MEGA file of the tRNA was analyzed for transition/transversion bias using the MEGA6 software based on the following statistical parameters: analysis, estimated transition/transversion bias (MCL); scope, all selected taxa; statistical method, maximum composite likelihood (MCL); substitution type, nucleotides; model/method, Tamura-Nei model; gaps/missing data treatment, partial deletion, and site coverage cutoff, 95%.

## Author contributions

TM: Conceived the idea, performed the experiments and statistical analysis, drafted and revised the manuscript; HB: revised the manuscript.

### Conflict of interest statement

The authors declare that the research was conducted in the absence of any commercial or financial relationships that could be construed as a potential conflict of interest.

## References

[B1] Abelson J. Trotta C. R. Li H. (1998). tRNA splicing. J. Biol. Chem. 273, 12685–12688. 10.1074/jbc.273.21.12685 9582290

[B2] Agris P. F. Vendeix F. A. P. Graham W. D. (2007). tRNA's wobble decoding of the genome: 40 years of modification. J. Mol. Biol. 366, 1–13. 10.1016/j.jmb.2006.11.046 17187822

[B3] Akama K. Junker V. Beier H. (2000). Identification of two catalytic subunits of tRNA splicing endonuclease from *Arabidopsis thaliana*. Gene 257, 177–185. 10.1016/S0378-1119(00)00408-X 11080584

[B4] Akama K. Naß A. Junker V. Beier H. (1997). Characterization of nuclear tRNATyr introns: their evolution from red algae to higher plants. FEBS Lett. 417, 213–218. 10.1016/S0014-5793(97)01288-X 9395298

[B5] Bermudez-Santana C. Attolini C. S.-O. Kirsten T. Engelhardt J. Prohaska S. J. Steigele S.. (2010). Genomic organization of eukaryotic tRNAs. BMC Genomics 11:270. 10.1186/1471-2164-11-270 20426822 PMC2888827

[B6] Betat H. Mörl M. (2015). The CCA-adding enzyme: a central scrutinizer in tRNA quality control. Bioessays 37, 975–982. 10.1002/bies.201500043 26172425

[B7] Eigen M. Lindemann B. F. Tietze M. Winkler-oswatitsch R. Dress A. Haeseler A. V. O. N. (1989). How old is the genetic code? Statistical geometry of tRNA provides an answer. Science 244, 673–679. 10.1126/science.2497522 2497522

[B8] Eigen M. Winkler-Oswatitsch R. (1981). Transfer-RNA, an early gene? Naturwissenschaften 68, 282–292. 10.1007/BF01047470 7266675

[B9] Gaus D. Gruter F. Sprinzl M. (1979). Proposed numbering system of nucleotides in tRNAs based on yeast tRNA, in Transfer RNA: Structure, Properties, and Recognition, eds Schimmel P. R. Söll D. Abelson J. N. (New York, NY; Cold Spring Harbor, NY: Cold Spring Harbor Laboratory Press), 818–519.

[B10] Goodenbour J. M. Pan T. (2006). Diversity of tRNA genes in eukaryotes. Nucleic Acids Res. 34, 6137–6146. 10.1093/nar/gkl725 17088292 PMC1693877

[B11] Holmquist R. Cantor C. Jukes T. (1972). Improved procedures for comparing homologous sequences in molecules of proteins and nucleic acids. J. Mol. Biol. 64, 145–161. 10.1016/0022-2836(72)90326-9 5015396

[B12] Hou Y.-M. (2010). CCA addition to tRNA: implications for tRNA quality control. IUBMB Life 62, 251–260. 10.1002/iub.301 20101632 PMC2848691

[B13] Kirchner S. Ignatova Z. (2014). Emerging roles of tRNA in adaptive translation, signalling dynamics and disease. Nat. Rev. Genet. 16, 98–112. 10.1038/nrg3861 25534324

[B14] Lowe T. M. Eddy S. R. (1997). tRNAscan-SE: a program for improved detection of transfer RNA genes in genomic sequence. Nucleic Acids Res. 25, 955–964. 10.1093/nar/25.5.0955 9023104 PMC146525

[B15] Mallick B. Chakrabarti J. Sahoo S. Ghosh Z. Das S. (2005). Identity elements of Archaeal tRNA. DNA Res. 12, 235–246. 10.1093/dnares/dsi008 16769686

[B16] Michaud M. Cognat V. Duchêne A. M. Maréchal-Drouard L. (2011). A global picture of tRNA genes in plant genomes. Plant J. 66, 80–93. 10.1111/j.1365-313X.2011.04490.x 21443625

[B17] Mohanta T. Bae H. (2015). The diversity of fungal genome. Biol. Proced. Online 17, 8. 10.1186/s12575-015-0020-z 25866485 PMC4392786

[B18] Mohanta T. Malnoy M. Mohanta N. Kanchiswamy C. (2014). In-silico identification and phylogenetic analysis of auxin efflux carrier gene family in *Setaria italica* L. African J. Biotechnol. 13, 211–225. 10.5897/AJB2014.13617

[B19] Mohanta T. Mohanta N. Bae H. (2015). Identification and expression analysis of PIN-Like (PILS) gene family of rice treated with auxin and cytokinin. Genes 6, 622–640. 10.3390/genes6030622 26193322 PMC4584321

[B20] Mohanta T. K. Arora P. K. Mohanta N. Parida P. Bae H. (2015a). Identification of new members of the MAPK gene family in plants shows diverse conserved domains and novel activation loop variants. BMC Genomics 16:58. 10.1186/s12864-015-1244-7 25888265 PMC4363184

[B21] Mohanta T. K. Bae H. (2017). Cloning and characterization of auxin efflux carrier genes EcPIN1a and EcPIN1b from finger millet *Eleusine coracana* L. 3 Biotech 7, 51. 10.1007/s13205-017-0689-6 28444595 PMC5428087

[B22] Mohanta T. K. Mohanta N. Mohanta Y. K. Parida P. Bae H. (2015b). Genome-wide identification of Calcineurin B-Like (CBL) gene family of plants reveals novel conserved motifs and evolutionary aspects in calcium signaling events. BMC Plant Biol. 15:189. 10.1186/s12870-015-0543-0 26245459 PMC4527274

[B23] Mohanta T. K. Pudake R. N. Bae H. (2017). Genome-wide identification of major protein families of cyanobacteria and genomic insight into the circadian rhythm. Eur. J. Phycol. 52, 149–165. 10.1080/09670262.2016.1251619

[B24] Phizicky E. M. Hopper A. K. (2010). tRNA biology charges to the front. Genes Dev. 24, 1832–1860. 10.1101/gad.1956510 20810645 PMC2932967

[B25] Rogers T. E. Ataide S. F. Dare K. Katz A. Seveau S. Roy H.. (2012). A pseudo-tRNA modulates antibiotic resistance in Bacillus cereus. PLoS ONE 7:e41248. 10.1371/journal.pone.0041248 22815980 PMC3399842

[B26] Schimmel P. Tamura K. (2003). tRNA structure goes from L to lambda. Cell 113, 276–278. 10.1016/S0092-8674(03)00313-1 12732135

[B27] Shi P. Y. Maizels N. Weiner A. M. (1998). CCA addition by tRNA nucleotidyltransferase: polymerization without translocation? EMBO J. 17, 3197–3206. 10.1093/emboj/17.11.3197 9606201 PMC1170658

[B28] Stange N. Beier D. Beier H. (1992). Intron excision from tRNA precursors by plant splicing endonuclease requires unique features of the mature tRNA domain. Eur. J. Biochem. 210, 193–203. 10.1111/j.1432-1033.1992.tb17408.x 1332859

[B29] Sugahara J. Yachie N. Sekine Y. Soma A. Matsui M. Tomita M.. (2006). SPLITS: a new program for predicting split and intron-containing tRNA genes at the genome level. In Silico Biol. 6, 411–418. 17274770

[B30] Tamura K. Stecher G. Peterson D. Filipski A. Kumar S. (2013). MEGA6: molecular evolutionary genetics analysis version 6.0. Mol. Biol. Evol. 30, 2725–2729. 10.1093/molbev/mst197 24132122 PMC3840312

[B31] Tocchini-Valentini G. Saks M. E. Abelson J. (2000). tRNA leucine identity and recognition sets. J. Mol. Biol. 298, 779–793. 10.1006/jmbi.2000.3694 10801348

[B32] Tocchini-Valentini G. D. Fruscoloni P. Tocchini-Valentini G. P. (2009). Processing of multiple-intron-containing pretRNA. Proc. Natl. Acad. Sci. U.S.A. 106, 20246–20251. 10.1073/pnas.0911658106 19910528 PMC2787110

[B33] Torres A. G. Batlle E. Ribas de Pouplana L. (2014). Role of tRNA modifications in human diseases. Trends Mol. Med. 20, 306–314. 10.1016/j.molmed.2014.01.008 24581449

[B34] Vortler S. Morl M. (2010). tRNA-nucleotidyltransferases: highly unusual RNA polymerases with vital functions. FEBS Lett. 584, 297–302. 10.1016/j.febslet.2009.10.078 19883645

[B35] Yoshihisa T. (2014). Handling tRNA introns, archaeal way and eukaryotic way. Front. Genet. 5:213. 10.3389/fgene.2014.00213 25071838 PMC4090602

[B36] Yue D. Maizels N. Weiner A. M. (1996). CCA-adding enzymes and poly(A) polymerases are all members of the same nucleotidyltransferase superfamily: characterization of the CCA-adding enzyme from the archaeal hyperthermophile *Sulfolobus shibatae*. RNA 2, 895–908. 8809016 PMC1369424

[B37] Zuckerkandl E. Pauling I. (1962). Molecular disease, evolution and genetic heterogeneity, in Horizons in Biochemistry, eds Kasha M. Pullman B. (New York, NY: Academic Press), 189–225.

[B38] Zuo Z. Peng D. Yin X. Zhou X. Cheng H. Zhou R. (2013). Genome-wide analysis reveals origin of transfer RNA genes from tRNA halves. Mol. Biol. Evol. 30, 2087–2098. 10.1093/molbev/mst107 23744908

