# Supplementary material for: Analyses of Genomic tRNA Reveal Presence of Novel tRNAs in Oryza sativa
Source: Front Genet. 2017 Jun 30;8:90. doi: 10.3389/fgene.2017.00090 (PMC5492330; doi:10.3389/fgene.2017.00090)
Supplement: Supplementary file 1 [file Table1.DOCX]

**Supplementary Table 1.**

Nucleotide composition of acceptor arm, D and D-loop of *O. sativa* tRNA.

| tRNA No. | tRNA Codes for | Acceptor Arm | |  | D stem | | D loop |
| --- | --- | --- | --- | --- | --- | --- | --- |
|  |  | No. of Nucleotides | |  | No. of Nucleotides | | No. of Nucleotides |
| chr6.trna18 | Ala | 7 |  |  | 4 |  | 8 |
| chr3.trna12 | Ala | 7 |  |  | 4 |  | 8 |
| chr11.trna32 | Ala | 7 |  |  | 4 |  | 8 |
| chr1.trna62 | Ala | 7 |  |  | 4 |  | 8 |
| chr10.trna26 | Ala | 7 |  |  | 4 |  | 8 |
| chr10.trna39 | Ala | 7 |  |  | 4 |  | 8 |
| chr11.trna14 | Ala | 7 |  |  | 4 |  | 8 |
| chr12.trna69 | Ala | 7 |  |  | 4 |  | 8 |
| chr3.trna50 | Ala | 7 |  |  | 4 |  | 8 |
| chr3.trna63 | Ala | 7 |  |  | 4 |  | 8 |
| chr3.trna68 | Ala | 7 |  |  | 4 |  | 8 |
| chr4.trna31 | Ala | 7 |  |  | 4 |  | 8 |
| chr5.trna21 | Ala | 7 |  |  | 4 |  | 8 |
| chr6.trna16 | Ala | 7 |  |  | 4 |  | 8 |
| chr6.trna35 | Ala | 7 |  |  | 4 |  | 8 |
| chr6.trna37 | Ala | 7 |  |  | 4 |  | 8 |
| chr7.trna24 | Ala | 7 |  |  | 4 |  | 8 |
| chr8.trna19 | Ala | 7 |  |  | 4 |  | 8 |
| chr8.trna20 | Ala | 7 |  |  | 4 |  | 8 |
| chr9.trna22 | Ala | 7 |  |  | 4 |  | 8 |
| chr6.trna1 | Ala | 7 |  |  | 4 |  | 8 |
| chr4.trna53 | Ala | 7 |  |  | 4 |  | 8 |
| chr2.trna3 | Ala | 7 |  |  | 4 |  | 8 |
| chr3.trna9 | Ala | 7 |  |  | 4 |  | 8 |
| chr4.trna60 | Ala | 7 |  |  | 4 |  | 8 |
| chr7.trna29 | Ala | 7 |  |  | 4 |  | 8 |
| chr1.trna16 | Ala | 7 |  |  | 4 |  | 8 |
| chr2.trna19 | Ala | 7 |  |  | 4 |  | 8 |
| chr2.trna36 | Ala | 7 |  |  | 4 |  | 8 |
| chr4.trna37 | Ala | 7 |  |  | 4 |  | 8 |
| chr8.trna26 | Ala | 7 |  |  | 4 |  | 8 |
| chr9.trna14 | Ala | 7 |  |  | 4 |  | 8 |
| chr1.trna43 | Ala | 7 |  |  | 3 |  | 12 |
| chr1.trna48 | Ala | 7 |  |  | 4 |  | 8 |
| chr10.trna29 | Ala | 7 |  |  | 4 |  | 8 |
| chr11.trna30 | Ala | 7 |  |  | 4 |  | 8 |
| chr11.trna33 | Ala | 7 |  |  | 4 |  | 8 |
| chr12.trna39 | Ala | 7 |  |  | 4 |  | 8 |
| chr12.trna70 | Ala | 7 |  |  | 4 |  | 8 |
| chr12.trna9 | Ala | 7 |  |  | 4 |  | 8 |
| chr2.trna23 | Ala | 7 |  |  | 4 |  | 8 |
| chr2.trna48 | Ala | 7 |  |  | 4 |  | 8 |

| chr4.trna45 | Ala | 7 |  |  | 4 |  | 8 |
| --- | --- | --- | --- | --- | --- | --- | --- |
| chr7.trna17 | Arg | 7 |  |  | 4 |  | 9 |
| chr12.trna64 | Arg | 7 |  |  | 4 |  | 9 |
| chr5.trna31 | Arg | 7 |  |  | 2 |  | 10 |
| chr1.trna38 | Arg | 7 |  |  | 4 |  | 9 |
| chr10.trna15 | Arg | 7 |  |  | 4 |  | 9 |
| chr10.trna20 | Arg | 7 |  |  | 4 |  | 9 |
| chr3.trna30 | Arg | 7 |  |  | 4 |  | 9 |
| chr3.trna64 | Arg | 7 |  |  | 4 |  | 9 |
| chr4.trna17 | Arg | 7 |  |  | 4 |  | 9 |
| chr4.trna21 | Arg | 7 |  |  | 4 |  | 9 |
| chr4.trna28 | Arg | 7 |  |  | 4 |  | 9 |
| chr4.trna4 | Arg | 7 |  |  | 4 |  | 9 |
| chr8.trna48 | Arg | 7 |  |  | 4 |  | 9 |
| chr12.trna33 | Arg | 7 |  |  | 3 |  | 10 |
| chr10.trna16 | Arg | 7 |  |  | 3 |  | 10 |
| chr3.trna21 | Arg | 7 |  |  | 3 |  | 10 |
| chr3.trna4 | Arg | 7 |  |  | 3 |  | 10 |
| chr4.trna38 | Arg | 7 |  |  | 3 |  | 10 |
| chr1.trna20 | Arg | 7 |  |  | 3 |  | 10 |
| chr1.trna27 | Arg | 7 |  |  | 3 |  | 10 |
| chr3.trna29 | Arg | 7 |  |  | 3 |  | 10 |
| chr5.trna36 | Arg | 7 |  |  | 3 |  | 10 |
| chr5.trna44 | Arg | 7 |  |  | 3 |  | 10 |
| chr9.trna39 | Arg | 7 |  |  | 3 |  | 10 |
| chr3.trna25 | Arg | 7 |  |  | 4 |  | 8 |
| chr4.trna59 | Arg | 7 |  |  | 4 |  | 8 |
| chr8.trna10 | Arg | 7 |  |  | 4 |  | 8 |
| chr8.trna39 | Arg | 7 |  |  | 4 |  | 8 |
| chr9.trna21 | Arg | 7 |  |  | 4 |  | 8 |
| chr4.trna43 | Arg | 7 |  |  | 4 |  | 8 |
| chr1.trna41 | Arg | 7 |  |  | 4 |  | 8 |
| chr5.trna40 | Arg | 7 |  |  | 4 |  | 8 |
| chr1.trna92 | Arg | 4 |  |  | 4 |  | 8 |
| chr5.trna9 | Arg | 7 |  |  | 4 |  | 8 |
| chr1.trna91 | Arg | 7 |  |  | 4 |  | 8 |
| chr11.trna27 | Arg | 7 |  |  | 4 |  | 8 |
| chr12.trna41 | Arg | 7 |  |  | 4 |  | 8 |
| chr12.trna67 | Arg | 7 |  |  | 4 |  | 8 |
| chr3.trna57 | Arg | 7 |  |  | 4 |  | 8 |
| chr5.trna27 | Arg | 7 |  |  | 4 |  | 8 |
| chr3.trna54 | Arg | 7 |  |  | 4 |  | 8 |
| chr10.trna2 | Arg | 7 |  |  | 4 |  | 8 |
| chr3.trna72 | Arg | 7 |  |  | 4 |  | 8 |

| chr5.trna3 | Arg | 7 |  |  | 4 |  | 8 |
| --- | --- | --- | --- | --- | --- | --- | --- |
| chr1.trna52 | Arg | 7 |  |  | 4 |  | 9 |
| chr3.trna6 | Arg | 7 |  |  | 4 |  | 9 |
| chr5.trna4 | Arg | 7 |  |  | 4 |  | 9 |
| chr3.trna55 | Arg | 7 |  |  | 4 |  | 8 |
| chr10.trna58 | Arg | 7 |  |  | 4 |  | 8 |
| chr10.trna23 | Arg | 7 |  |  | 4 |  | 8 |
| chr4.trna97 | Arg | 7 |  |  | 4 |  | 8 |
| chr9.trna3 | Arg | 7 |  |  | 4 |  | 8 |
| chr8.trna11 | Arg | 7 |  |  | 4 |  | 8 |
| chr8.trna17 | Arg | 7 |  |  | 4 |  | 8 |
| chr8.trna50 | Arg | 7 |  |  | 4 |  | 8 |
| chr9.trna19 | Arg | 7 |  |  | 4 |  | 8 |
| chr1.trna57 | Arg | 7 |  |  | 4 |  | 8 |
| chr11.trna8 | Arg | 7 |  |  | 4 |  | 8 |
| chr12.trna6 | Arg | 7 |  |  | 4 |  | 8 |
| chr2.trna76 | Asn | 7 |  |  | 4 |  | 7 |
| chr4.trna40 | Asn | 7 |  |  | 4 |  | 7 |
| chr12.trna65 | Asn | 7 |  |  | 4 |  | 7 |
| chr11.trna19 | Asn | 7 |  |  | 4 |  | 9 |
| chr9.trna26 | Asn | 7 |  |  | 4 |  | 9 |
| chr1.trna18 | Asn | 7 |  |  | 4 |  | 7 |
| chr1.trna73 | Asn | 7 |  |  | 4 |  | 7 |
| chr10.trna38 | Asn | 7 |  |  | 4 |  | 7 |
| chr10.trna51 | Asn | 7 |  |  | 4 |  | 7 |
| chr12.trna12 | Asn | 7 |  |  | 4 |  | 7 |
| chr12.trna25 | Asn | 7 |  |  | 4 |  | 7 |
| chr3.trna15 | Asn | 7 |  |  | 4 |  | 7 |
| chr3.trna53 | Asn | 7 |  |  | 4 |  | 7 |
| chr4.trna77 | Asn | 7 |  |  | 4 |  | 7 |
| chr4.trna99 | Asn | 7 |  |  | 4 |  | 7 |
| chr8.trna12 | Asn | 7 |  |  | 4 |  | 7 |
| chr3.trna14 | Asn | 7 |  |  | 4 |  | 9 |
| chr3.trna78 | Asn | 7 |  |  | 4 |  | 9 |
| chr5.trna47 | Asn | 7 |  |  | 4 |  | 9 |
| chr10.trna24 | Asn | 7 |  |  | 4 |  | 9 |
| chr7.trna35 | Asn | 7 |  |  | 4 |  | 9 |
| chr1.trna14 | Asn | 7 |  |  | 4 |  | 9 |
| chr1.trna15 | Asn | 7 |  |  | 4 |  | 9 |
| chr1.trna17 | Asn | 7 |  |  | 4 |  | 9 |
| chr10.trna41 | Asn | 7 |  |  | 4 |  | 9 |
| chr3.trna81 | Asn | 7 |  |  | 4 |  | 9 |
| chr5.trna56 | Asn | 7 |  |  | 4 |  | 9 |
| chr5.trna48 | Asn | 7 |  |  | 4 |  | 9 |

| chr5.trna58 | Asn | 7 |  |  | 4 |  | 9 |
| --- | --- | --- | --- | --- | --- | --- | --- |
| chr10.trna9 | Asp | 7 |  |  | 4 |  | 9 |
| chr1.trna80 | Asp | 7 |  |  | 4 |  | 9 |
| chr7.trna16 | Asp | 7 |  |  | 4 |  | 9 |
| chr11.trna28 | Asp | 7 |  |  | 4 |  | 9 |
| chr2.trna6 | Asp | 7 |  |  | 4 |  | 8 |
| chr5.trna23 | Asp | 7 |  |  | 4 |  | 8 |
| chr2.trna58 | Asp | 7 |  |  | 4 |  | 8 |
| chr4.trna15 | Asp | 7 |  |  | 4 |  | 9 |
| chr4.trna26 | Asp | 7 |  |  | 4 |  | 9 |
| chr5.trna16 | Asp | 7 |  |  | 4 |  | 8 |
| chr8.trna18 | Asp | 7 |  |  | 4 |  | 8 |
| chr7.trna1 | Asp | 7 |  |  | 4 |  | 8 |
| chr1.trna5 | Asp | 7 |  |  | 4 |  | 8 |
| chr1.trna81 | Asp | 7 |  |  | 4 |  | 8 |
| chr1.trna84 | Asp | 7 |  |  | 4 |  | 8 |
| chr1.trna87 | Asp | 7 |  |  | 4 |  | 8 |
| chr10.trna49 | Asp | 7 |  |  | 4 |  | 8 |
| chr3.trna19 | Asp | 7 |  |  | 4 |  | 8 |
| chr3.trna20 | Asp | 7 |  |  | 4 |  | 8 |
| chr3.trna76 | Asp | 7 |  |  | 4 |  | 8 |
| chr4.trna2 | Asp | 7 |  |  | 4 |  | 8 |
| chr5.trna15 | Asp | 7 |  |  | 4 |  | 8 |
| chr5.trna17 | Asp | 7 |  |  | 4 |  | 8 |
| chr5.trna18 | Asp | 7 |  |  | 4 |  | 8 |
| chr5.trna19 | Asp | 7 |  |  | 4 |  | 8 |
| chr5.trna25 | Asp | 7 |  |  | 4 |  | 8 |
| chr6.trna2 | Asp | 7 |  |  | 4 |  | 8 |
| chr6.trna38 | Asp | 7 |  |  | 4 |  | 8 |
| chr7.trna34 | Asp | 7 |  |  | 4 |  | 8 |
| chr9.trna8 | Asp | 7 |  |  | 4 |  | 8 |
| chr9.trna9 | Asp | 7 |  |  | 4 |  | 8 |
| chr12.trna54 | Asp | 7 |  |  | 4 |  | 9 |
| chr2.trna2 | Cys | 7 |  |  | 4 |  | 9 |
| chr4.trna29 | Cys | 7 |  |  | 3 |  | 9 |
| chr10.trna43 | Cys | 7 |  |  | 3 |  | 9 |
| chr10.trna59 | Cys | 7 |  |  | 3 |  | 9 |
| chr2.trna12 | Cys | 7 |  |  | 3 |  | 9 |
| chr4.trna78 | Cys | 7 |  |  | 3 |  | 9 |
| chr4.trna86 | Cys | 7 |  |  | 3 |  | 9 |
| chr2.trna50 | Cys | 7 |  |  | 4 |  | 7 |
| chr4.trna73 | Cys | 7 |  |  | 4 |  | 7 |
| chr7.trna5 | Cys | 7 |  |  | 4 |  | 7 |
| chr1.trna56 | Cys | 7 |  |  | 4 |  | 7 |

| chr2.trna32 | Cys | 7 |  |  | 4 |  | 7 |
| --- | --- | --- | --- | --- | --- | --- | --- |
| chr5.trna1 | Cys | 7 |  |  | 4 |  | 7 |
| chr4.trna47 | Cys | 7 |  |  | 4 |  | 7 |
| chr1.trna99 | Cys | 7 |  |  | 4 |  | 7 |
| chr3.trna33 | Cys | 7 |  |  | 4 |  | 7 |
| chr12.trna23 | Cys | 7 |  |  | 4 |  | 9 |
| chr9.trna5 | Cys | 7 |  |  | 4 |  | 9 |
| chr3.trna32 | Gln | 7 |  |  | 4 |  | 8 |
| chr12.trna38 | Gln | 7 |  |  | 4 |  | 8 |
| chr1.trna29 | Gln | 7 |  |  | 4 |  | 8 |
| chr1.trna11 | Gln | 7 |  |  | 4 |  | 8 |
| chr5.trna7 | Gln | 7 |  |  | 4 |  | 8 |
| chr6.trna25 | Gln | 7 |  |  | 4 |  | 8 |
| chr2.trna9 | Gln | 7 |  |  | 4 |  | 8 |
| chr3.trna26 | Gln | 7 |  |  | 4 |  | 8 |
| chr2.trna27 | Gln | 7 |  |  | 4 |  | 8 |
| chr6.trna29 | Gln | 7 |  |  | 4 |  | 8 |
| chr10.trna33 | Gln | 7 |  |  | 3 |  | 9 |
| chr6.trna30 | Gln | 7 |  |  | 3 |  | 9 |
| chr12.trna28 | Gln | 7 |  |  | 3 |  | 9 |
| chr1.trna98 | Gln | 7 |  |  | 3 |  | 9 |
| chr2.trna68 | Gln | 7 |  |  | 4 |  | 8 |
| chr10.trna63 | Gln | 7 |  |  | 3 |  | 9 |
| chr4.trna84 | Gln | 7 |  |  | 3 |  | 9 |
| chr4.trna90 | Gln | 7 |  |  | 3 |  | 9 |
| chr7.trna39 | Gln | 7 |  |  | 3 |  | 9 |
| chr2.trna64 | Gln | 7 |  |  | 4 |  | 8 |
| chr2.trna67 | Gln | 7 |  |  | 4 |  | 8 |
| chr2.trna69 | Gln | 7 |  |  | 4 |  | 8 |
| chr2.trna65 | Gln | 7 |  |  | 4 |  | 8 |
| chr2.trna66 | Gln | 7 |  |  | 4 |  | 8 |
| chr2.trna70 | Gln | 7 |  |  | 4 |  | 8 |
| chr2.trna63 | Gln | 7 |  |  | 4 |  | 8 |
| chr2.trna17 | Gln | 7 |  |  | 4 |  | 8 |
| chr3.trna43 | Gln | 7 |  |  | 4 |  | 8 |
| chr1.trna66 | Gln | 7 |  |  | 4 |  | 8 |
| chr4.trna51 | Gln | 7 |  |  | 4 |  | 8 |
| chr8.trna52 | Gln | 7 |  |  | 4 |  | 8 |
| chr7.trna11 | Glu | 7 |  |  | 4 |  | 15 |
| chr3.trna46 | Glu | 7 |  |  | 4 |  | 9 |
| chr1.trna21 | Glu | 7 |  |  | 4 |  | 9 |
| chr5.trna39 | Glu | 7 |  |  | 4 |  | 9 |
| chr1.trna71 | Glu | 7 |  |  | 4 |  | 9 |
| chr4.trna62 | Glu | 7 |  |  | 4 |  | 9 |

| chr6.trna13 | Glu | 7 |  |  | 4 |  | 9 |
| --- | --- | --- | --- | --- | --- | --- | --- |
| chr2.trna39 | Glu | 7 |  |  | 4 |  | 9 |
| chr1.trna10 | Glu | 7 |  |  | 4 |  | 9 |
| chr1.trna3 | Glu | 7 |  |  | 4 |  | 9 |
| chr1.trna70 | Glu | 7 |  |  | 4 |  | 9 |
| chr1.trna9 | Glu | 7 |  |  | 4 |  | 9 |
| chr2.trna35 | Glu | 7 |  |  | 4 |  | 9 |
| chr2.trna72 | Glu | 7 |  |  | 4 |  | 9 |
| chr2.trna74 | Glu | 7 |  |  | 4 |  | 9 |
| chr3.trna66 | Glu | 7 |  |  | 4 |  | 9 |
| chr5.trna32 | Glu | 7 |  |  | 4 |  | 9 |
| chr5.trna46 | Glu | 7 |  |  | 4 |  | 9 |
| chr5.trna49 | Glu | 7 |  |  | 4 |  | 9 |
| chr7.trna44 | Glu | 7 |  |  | 4 |  | 9 |
| chr8.trna30 | Glu | 7 |  |  | 4 |  | 9 |
| chr8.trna35 | Glu | 7 |  |  | 4 |  | 9 |
| chr9.trna1 | Glu | 7 |  |  | 4 |  | 9 |
| chr3.trna37 | Glu | 7 |  |  | 4 |  | 9 |
| chr1.trna78 | Glu | 7 |  |  | 4 |  | 9 |
| chr10.trna7 | Glu | 7 |  |  | 4 |  | 9 |
| chr2.trna56 | Glu | 7 |  |  | 4 |  | 9 |
| chr4.trna13 | Glu | 7 |  |  | 4 |  | 9 |
| chr4.trna24 | Glu | 7 |  |  | 4 |  | 9 |
| chr7.trna2 | Glu | 7 |  |  | 4 |  | 10 |
| chr11.trna12 | Glu | 7 |  |  | 4 |  | 10 |
| chr12.trna37 | Glu | 7 |  |  | 4 |  | 10 |
| chr12.trna43 | Glu | 7 |  |  | 4 |  | 10 |
| chr3.trna52 | Glu | 7 |  |  | 4 |  | 10 |
| chr12.trna5 | Glu | 7 |  |  | 4 |  | 10 |
| chr3.trna73 | Glu | 7 |  |  | 4 |  | 10 |
| chr4.trna56 | Glu | 7 |  |  | 4 |  | 10 |
| chr8.trna4 | Glu | 7 |  |  | 4 |  | 10 |
| chr8.trna5 | Glu | 7 |  |  | 4 |  | 10 |
| chr11.trna6 | Glu | 7 |  |  | 4 |  | 8 |
| chr3.trna5 | Gly | 7 |  |  | 3 |  | 9 |
| chr1.trna85 | Gly | 7 |  |  | 3 |  | 9 |
| chr8.trna42 | Gly | 7 |  |  | 3 |  | 9 |
| chr2.trna8 | Gly | 7 |  |  | 3 |  | 9 |
| chr1.trna86 | Gly | 7 |  |  | 3 |  | 9 |
| chr12.trna30 | Gly | 7 |  |  | 3 |  | 9 |
| chr3.trna10 | Gly | 7 |  |  | 3 |  | 9 |
| chr7.trna30 | Gly | 7 |  |  | 3 |  | 9 |
| chr9.trna29 | Gly | 7 |  |  | 3 |  | 9 |
| chr2.trna54 | Gly | 7 |  |  | 3 |  | 9 |

| chr12.trna57 | Gly | 7 |  |  | 3 |  | 9 |
| --- | --- | --- | --- | --- | --- | --- | --- |
| chr1.trna8 | Gly | 7 |  |  | 4 |  | 7 |
| chr12.trna36 | Gly | 7 |  |  | 4 |  | 7 |
| chr1.trna37 | Gly | 7 |  |  | 3 |  | 9 |
| chr10.trna5 | Gly | 7 |  |  | 3 |  | 9 |
| chr4.trna11 | Gly | 7 |  |  | 3 |  | 9 |
| chr4.trna22 | Gly | 7 |  |  | 3 |  | 9 |
| chr7.trna8 | Gly | 7 |  |  | 4 |  | 7 |
| chr2.trna42 | Gly | 7 |  |  | 4 |  | 7 |
| chr7.trna28 | Gly | 7 |  |  | 4 |  | 7 |
| chr1.trna19 | Gly | 7 |  |  | 4 |  | 7 |
| chr10.trna30 | Gly | 7 |  |  | 4 |  | 7 |
| chr2.trna52 | Gly | 7 |  |  | 4 |  | 7 |
| chr2.trna71 | Gly | 7 |  |  | 4 |  | 7 |
| chr3.trna1 | Gly | 7 |  |  | 4 |  | 7 |
| chr3.trna24 | Gly | 7 |  |  | 4 |  | 7 |
| chr3.trna34 | Gly | 7 |  |  | 4 |  | 7 |
| chr3.trna77 | Gly | 7 |  |  | 4 |  | 7 |
| chr3.trna8 | Gly | 7 |  |  | 4 |  | 7 |
| chr4.trna63 | Gly | 7 |  |  | 4 |  | 7 |
| chr5.trna14 | Gly | 7 |  |  | 4 |  | 7 |
| chr5.trna29 | Gly | 7 |  |  | 4 |  | 7 |
| chr5.trna55 | Gly | 7 |  |  | 4 |  | 7 |
| chr6.trna24 | Gly | 7 |  |  | 4 |  | 7 |
| chr6.trna4 | Gly | 7 |  |  | 4 |  | 7 |
| chr7.trna7 | Gly | 7 |  |  | 4 |  | 7 |
| chr8.trna44 | Gly | 7 |  |  | 4 |  | 7 |
| chr11.trna23 | Gly | 7 |  |  | 4 |  | 8 |
| chr12.trna45 | Gly | 7 |  |  | 4 |  | 8 |
| chr3.trna67 | Gly | 7 |  |  | 4 |  | 8 |
| chr7.trna36 | Gly | 7 |  |  | 4 |  | 8 |
| chr7.trna6 | Gly | 7 |  |  | 4 |  | 8 |
| chr8.trna2 | Gly | 7 |  |  | 4 |  | 8 |
| chr8.trna3 | Gly | 7 |  |  | 4 |  | 8 |
| chr5.trna38 | Gly | 7 |  |  | 4 |  | 8 |
| chr4.trna32 | Gly | 7 |  |  | 4 |  | 8 |
| chr11.trna7 | His | 7 |  |  | 3 |  | 11 |
| chr4.trna41 | His | 7 |  |  | 3 |  | 11 |
| chr10.trna64 | His | 7 |  |  | 3 |  | 11 |
| chr12.trna16 | His | 7 |  |  | 3 |  | 11 |
| chr10.trna27 | His | 7 |  |  | 3 |  | 11 |
| chr10.trna13 | His | 7 |  |  | 3 |  | 11 |
| chr5.trna52 | His | 7 |  |  | 4 |  | 8 |
| chr4.trna19 | His | 7 |  |  | 3 |  | 11 |

| chr4.trna7 | His | 7 |  |  | 3 |  | 11 |
| --- | --- | --- | --- | --- | --- | --- | --- |
| chr4.trna85 | His | 7 |  |  | 3 |  | 11 |
| chr4.trna91 | His | 7 |  |  | 3 |  | 11 |
| chr7.trna40 | His | 7 |  |  | 3 |  | 11 |
| chr8.trna27 | His | 7 |  |  | 3 |  | 11 |
| chr8.trna46 | His | 7 |  |  | 3 |  | 11 |
| chr9.trna28 | His | 7 |  |  | 3 |  | 11 |
| chr9.trna32 | His | 7 |  |  | 3 |  | 11 |
| chr1.trna13 | His | 7 |  |  | 4 |  | 8 |
| chr10.trna25 | His | 7 |  |  | 4 |  | 8 |
| chr3.trna7 | His | 7 |  |  | 4 |  | 8 |
| chr3.trna79 | His | 7 |  |  | 4 |  | 8 |
| chr4.trna75 | His | 7 |  |  | 4 |  | 8 |
| chr6.trna15 | His | 7 |  |  | 4 |  | 8 |
| chr7.trna18 | His | 7 |  |  | 4 |  | 8 |
| chr7.trna23 | His | 7 |  |  | 4 |  | 8 |
| chr7.trna27 | His | 7 |  |  | 4 |  | 8 |
| chr2.trna53 | His | 7 |  |  | 4 |  | 8 |
| chr4.trna42 | Ile | 7 |  |  | 4 |  | 9 |
| chr9.trna27 | Ile | 7 |  |  | 4 |  | 9 |
| chr8.trna28 | Ile | 7 |  |  | 4 |  | 9 |
| chr1.trna39 | Ile | 7 |  |  | 4 |  | 9 |
| chr1.trna45 | Ile | 7 |  |  | 4 |  | 9 |
| chr1.trna54 | Ile | 7 |  |  | 4 |  | 9 |
| chr1.trna90 | Ile | 7 |  |  | 4 |  | 9 |
| chr10.trna17 | Ile | 7 |  |  | 4 |  | 9 |
| chr11.trna1 | Ile | 7 |  |  | 4 |  | 9 |
| chr2.trna21 | Ile | 7 |  |  | 4 |  | 9 |
| chr3.trna62 | Ile | 7 |  |  | 4 |  | 9 |
| chr4.trna39 | Ile | 7 |  |  | 4 |  | 9 |
| chr4.trna64 | Ile | 7 |  |  | 4 |  | 9 |
| chr4.trna65 | Ile | 7 |  |  | 4 |  | 9 |
| chr5.trna37 | Ile | 7 |  |  | 4 |  | 9 |
| chr7.trna22 | Ile | 7 |  |  | 4 |  | 9 |
| chr8.trna41 | Ile | 7 |  |  | 4 |  | 9 |
| chr9.trna15 | Ile | 7 |  |  | 4 |  | 9 |
| chr12.trna13 | Ile | 7 |  |  | 4 |  | 9 |
| chr1.trna63 | Ile | 7 |  |  | 4 |  | 9 |
| chr1.trna96 | Ile | 7 |  |  | 4 |  | 9 |
| chr10.trna44 | Ile | 7 |  |  | 4 |  | 9 |
| chr5.trna10 | Ile | 7 |  |  | 4 |  | 9 |
| chr2.trna7 | Leu | 7 |  |  | 3 |  | 12 |
| chr1.trna1 | Leu | 7 |  |  | 3 |  | 12 |
| chr8.trna37 | Leu | 7 |  |  | 3 |  | 12 |
| chr1.trna47 | Leu | 7 |  |  | 3 |  | 12 |

| chr1.trna60 | Leu | 7 |  |  | 3 |  | 12 |
| --- | --- | --- | --- | --- | --- | --- | --- |
| chr1.trna72 | Leu | 7 |  |  | 3 |  | 12 |
| chr1.trna95 | Leu | 7 |  |  | 3 |  | 12 |
| chr2.trna51 | Leu | 7 |  |  | 3 |  | 12 |
| chr3.trna69 | Leu | 7 |  |  | 3 |  | 12 |
| chr4.trna33 | Leu | 7 |  |  | 3 |  | 12 |
| chr6.trna20 | Leu | 7 |  |  | 3 |  | 12 |
| chr8.trna33 | Leu | 7 |  |  | 3 |  | 12 |
| chr8.trna8 | Leu | 7 |  |  | 3 |  | 12 |
| chr9.trna17 | Leu | 7 |  |  | 3 |  | 12 |
| chr9.trna37 | Leu | 7 |  |  | 3 |  | 12 |
| chr9.trna34 | Leu | 7 |  |  | 2 |  | 12 |
| chr3.trna38 | Leu | 7 |  |  | 3 |  | 10 |
| chr10.trna3 | Leu | 7 |  |  | 3 |  | 10 |
| chr10.trna52 | Leu | 7 |  |  | 3 |  | 10 |
| chr2.trna59 | Leu | 7 |  |  | 3 |  | 10 |
| chr4.trna8 | Leu | 7 |  |  | 3 |  | 10 |
| chr4.trna81 | Leu | 7 |  |  | 3 |  | 10 |
| chr4.trna93 | Leu | 7 |  |  | 3 |  | 10 |
| chr5.trna8 | Leu | 7 |  |  | 3 |  | 10 |
| chr7.trna13 | Leu | 7 |  |  | 3 |  | 10 |
| chr8.trna13 | Leu | 7 |  |  | 3 |  | 10 |
| chr11.trna4 | Leu | 7 |  |  | 2 |  | 11 |
| chr10.trna45 | Leu | 7 |  |  | 3 |  | 11 |
| chr12.trna3 | Leu | 7 |  |  | 3 |  | 11 |
| chr2.trna33 | Leu | 7 |  |  | 3 |  | 11 |
| chr3.trna2 | Leu | 7 |  |  | 3 |  | 11 |
| chr3.trna36 | Leu | 7 |  |  | 3 |  | 11 |
| chr6.trna21 | Leu | 7 |  |  | 3 |  | 11 |
| chr7.trna10 | Leu | 7 |  |  | 3 |  | 11 |
| chr11.trna21 | Leu | 7 |  |  | 2 |  | 12 |
| chr9.trna38 | Leu | 7 |  |  | 2 |  | 12 |
| chr1.trna100 | Leu | 7 |  |  | 3 |  | 12 |
| chr2.trna45 | Leu | 7 |  |  | 3 |  | 12 |
| chr3.trna16 | Leu | 7 |  |  | 3 |  | 12 |
| chr5.trna42 | Leu | 7 |  |  | 3 |  | 12 |
| chr6.trna10 | Leu | 7 |  |  | 3 |  | 12 |
| chr6.trna11 | Leu | 7 |  |  | 3 |  | 12 |
| chr7.trna31 | Leu | 7 |  |  | 3 |  | 12 |
| chr9.trna13 | Leu | 7 |  |  | 3 |  | 12 |
| chr1.trna50 | Leu | 7 |  |  | 3 |  | 10 |
| chr11.trna24 | Leu | 7 |  |  | 3 |  | 10 |
| chr12.trna50 | Leu | 7 |  |  | 3 |  | 10 |
| chr4.trna67 | Leu | 7 |  |  | 3 |  | 10 |

| chr12.trna11 | Leu | 7 |  |  | 3 |  | 11 |
| --- | --- | --- | --- | --- | --- | --- | --- |
| chr10.trna50 | Leu | 7 |  |  | 3 |  | 11 |
| chr3.trna58 | Leu | 7 |  |  | 3 |  | 11 |
| chr6.trna14 | Leu | 7 |  |  | 3 |  | 11 |
| chr2.trna49 | Leu | 7 |  |  | 3 |  | 11 |
| chr1.trna49 | Leu | 7 |  |  | 3 |  | 11 |
| chr1.trna67 | Leu | 7 |  |  | 3 |  | 11 |
| chr5.trna12 | Leu | 7 |  |  | 3 |  | 11 |
| chr5.trna2 | Leu | 7 |  |  | 3 |  | 11 |
| chr4.trna72 | Leu | 7 |  |  | 3 |  | 11 |
| chr1.trna59 | Lys | 7 |  |  | 4 |  | 8 |
| chr1.trna26 | Lys | 7 |  |  | 4 |  | 8 |
| chr10.trna35 | Lys | 7 |  |  | 4 |  | 8 |
| chr12.trna32 | Lys | 7 |  |  | 4 |  | 8 |
| chr1.trna25 | Lys | 7 |  |  | 4 |  | 8 |
| chr1.trna42 | Lys | 7 |  |  | 4 |  | 8 |
| chr1.trna53 | Lys | 7 |  |  | 4 |  | 8 |
| chr10.trna34 | Lys | 7 |  |  | 4 |  | 8 |
| chr10.trna42 | Lys | 7 |  |  | 4 |  | 8 |
| chr11.trna22 | Lys | 7 |  |  | 4 |  | 8 |
| chr12.trna44 | Lys | 7 |  |  | 4 |  | 8 |
| chr3.trna18 | Lys | 7 |  |  | 4 |  | 8 |
| chr5.trna34 | Lys | 7 |  |  | 4 |  | 8 |
| chr5.trna35 | Lys | 7 |  |  | 4 |  | 8 |
| chr5.trna50 | Lys | 7 |  |  | 4 |  | 8 |
| chr8.trna23 | Lys | 7 |  |  | 4 |  | 8 |
| chr8.trna24 | Lys | 7 |  |  | 4 |  | 8 |
| chr8.trna25 | Lys | 7 |  |  | 4 |  | 8 |
| chr9.trna11 | Lys | 7 |  |  | 4 |  | 8 |
| chr9.trna33 | Lys | 7 |  |  | 4 |  | 8 |
| chr12.trna7 | Lys | 7 |  |  | 4 |  | 13 |
| chr10.trna18 | Lys | 7 |  |  | 4 |  | 8 |
| chr12.trna24 | Lys | 7 |  |  | 4 |  | 8 |
| chr5.trna41 | Lys | 7 |  |  | 4 |  | 8 |
| chr11.trna9 | Lys | 7 |  |  | 4 |  | 7 |
| chr12.trna8 | Lys | 7 |  |  | 4 |  | 7 |
| chr1.trna46 | Lys | 7 |  |  | 4 |  | 7 |
| chr2.trna10 | Lys | 7 |  |  | 4 |  | 7 |
| chr6.trna26 | Lys | 7 |  |  | 4 |  | 7 |
| chr9.trna24 | Lys | 7 |  |  | 4 |  | 7 |
| chr4.trna36 | Lys | 7 |  |  | 4 |  | 8 |
| chr6.trna34 | Lys | 7 |  |  | 4 |  | 8 |

| chr12.trna21 | Met | 6 |  |  | 4 |  | 11 |
| --- | --- | --- | --- | --- | --- | --- | --- |
| chr11.trna10 | Met | 7 |  |  | 4 |  | 8 |
| chr12.trna10 | Met | 7 |  |  | 4 |  | 8 |
| chr6.trna3 | Met | 7 |  |  | 4 |  | 8 |
| chr12.trna15 | Met | 7 |  |  | 4 |  | 8 |
| chr1.trna35 | Met | 7 |  |  | 4 |  | 8 |
| chr10.trna12 | Met | 7 |  |  | 4 |  | 8 |
| chr12.trna61 | Met | 7 |  |  | 4 |  | 8 |
| chr3.trna31 | Met | 7 |  |  | 4 |  | 8 |
| chr6.trna23 | Met | 7 |  |  | 3 |  | 10 |
| chr10.trna32 | Met | 7 |  |  | 3 |  | 10 |
| chr12.trna51 | Met | 7 |  |  | 3 |  | 11 |
| chr12.trna53 | Met | 7 |  |  | 3 |  | 11 |
| chr12.trna55 | Met | 7 |  |  | 3 |  | 11 |
| chr12.trna26 | Met | 7 |  |  | 4 |  | 9 |
| chr10.trna4 | Met | 7 |  |  | 3 |  | 10 |
| chr10.trna53 | Met | 7 |  |  | 3 |  | 10 |
| chr4.trna82 | Met | 7 |  |  | 3 |  | 10 |
| chr4.trna94 | Met | 7 |  |  | 3 |  | 10 |
| chr7.trna14 | Met | 7 |  |  | 3 |  | 10 |
| chr8.trna14 | Met | 7 |  |  | 3 |  | 10 |
| chr4.trna34 | Met | 7 |  |  | 4 |  | 7 |
| chr12.trna27 | Met | 7 |  |  | 4 |  | 8 |
| chr1.trna74 | Met | 7 |  |  | 4 |  | 9 |
| chr1.trna97 | Met | 7 |  |  | 4 |  | 9 |
| chr10.trna60 | Met | 7 |  |  | 4 |  | 9 |
| chr2.trna13 | Met | 7 |  |  | 4 |  | 9 |
| chr4.trna79 | Met | 7 |  |  | 4 |  | 9 |
| chr4.trna87 | Met | 7 |  |  | 4 |  | 9 |
| chr4.trna68 | Met | 7 |  |  | 3 |  | 10 |
| chr4.trna9 | Met | 7 |  |  | 3 |  | 10 |
| chr2.trna37 | Met | 7 |  |  | 4 |  | 7 |
| chr10.trna66 | Met | 7 |  |  | 4 |  | 7 |
| chr2.trna1 | Met | 7 |  |  | 4 |  | 7 |
| chr2.trna28 | Met | 7 |  |  | 4 |  | 7 |
| chr2.trna38 | Met | 7 |  |  | 4 |  | 7 |
| chr2.trna61 | Met | 7 |  |  | 4 |  | 7 |
| chr2.trna62 | Met | 7 |  |  | 4 |  | 7 |
| chr3.trna13 | Met | 7 |  |  | 4 |  | 7 |
| chr3.trna70 | Met | 7 |  |  | 4 |  | 7 |
| chr3.trna75 | Met | 7 |  |  | 4 |  | 7 |
| chr6.trna7 | Met | 7 |  |  | 4 |  | 7 |
| chr3.trna60 | Met | 7 |  |  | 4 |  | 9 |
| chr12.trna56 | Met | 7 |  |  | 4 |  | 9 |
| chr10.trna31 | Met | 7 |  |  | 4 |  | 9 |
| chr7.trna4 | Met | 7 |  |  | 4 |  | 9 |

| chr5.trna20 | Met | 7 |  |  | 4 |  | 9 |
| --- | --- | --- | --- | --- | --- | --- | --- |
| chr7.trna21 | Met | 7 |  |  | 4 |  | 9 |
| chr12.trna35 | Met | 7 |  |  | 4 |  | 9 |
| chr5.trna24 | Met | 7 |  |  | 4 |  | 9 |
| chr4.trna49 | Met | 7 |  |  | 4 |  | 9 |
| chr1.trna30 | Met | 7 |  |  | 4 |  | 9 |
| chr1.trna31 | Met | 7 |  |  | 4 |  | 9 |
| chr3.trna35 | Met | 7 |  |  | 4 |  | 9 |
| chr2.trna34 | Met | 7 |  |  | 4 |  | 9 |
| chr1.trna55 | Met | 7 |  |  | 4 |  | 9 |
| chr6.trna31 | Met | 7 |  |  | 3 |  | 10 |
| chr12.trna59 | Phe | 7 |  |  | 4 |  | 8 |
| chr1.trna83 | Phe | 7 |  |  | 4 |  | 8 |
| chr1.trna33 | Phe | 7 |  |  | 4 |  | 8 |
| chr1.trna76 | Phe | 7 |  |  | 4 |  | 8 |
| chr10.trna11 | Phe | 7 |  |  | 4 |  | 8 |
| chr10.trna37 | Phe | 7 |  |  | 4 |  | 8 |
| chr12.trna14 | Phe | 7 |  |  | 4 |  | 8 |
| chr8.trna29 | Phe | 7 |  |  | 4 |  | 8 |
| chr4.trna44 | Phe | 7 |  |  | 4 |  | 8 |
| chr1.trna101 | Phe | 7 |  |  | 4 |  | 8 |
| chr1.trna24 | Phe | 7 |  |  | 4 |  | 8 |
| chr1.trna58 | Phe | 7 |  |  | 4 |  | 8 |
| chr1.trna61 | Phe | 7 |  |  | 4 |  | 8 |
| chr2.trna18 | Phe | 7 |  |  | 4 |  | 8 |
| chr2.trna22 | Phe | 7 |  |  | 4 |  | 8 |
| chr4.trna35 | Phe | 7 |  |  | 4 |  | 8 |
| chr5.trna22 | Phe | 7 |  |  | 4 |  | 8 |
| chr7.trna9 | Phe | 7 |  |  | 4 |  | 8 |
| chr9.trna10 | Phe | 7 |  |  | 4 |  | 8 |
| chr11.trna11 | Pro | 7 |  |  | 3 |  | 9 |
| chr12.trna20 | Pro | 7 |  |  | 3 |  | 9 |
| chr1.trna7 | Pro | 7 |  |  | 3 |  | 9 |
| chr11.trna17 | Pro | 7 |  |  | 3 |  | 9 |
| chr11.trna20 | Pro | 7 |  |  | 3 |  | 9 |
| chr11.trna31 | Pro | 7 |  |  | 3 |  | 9 |
| chr12.trna18 | Pro | 7 |  |  | 3 |  | 9 |
| chr12.trna29 | Pro | 7 |  |  | 3 |  | 9 |
| chr12.trna31 | Pro | 7 |  |  | 3 |  | 9 |
| chr12.trna68 | Pro | 7 |  |  | 3 |  | 9 |
| chr5.trna6 | Pro | 7 |  |  | 3 |  | 9 |
| chr6.trna22 | Pro | 7 |  |  | 3 |  | 9 |
| chr11.trna15 | Pro | 7 |  |  | 4 |  | 7 |
| chr11.trna16 | Pro | 7 |  |  | 4 |  | 7 |
| chr11.trna2 | Pro | 7 |  |  | 3 |  | 10 |
| chr12.trna71 | Pro | 7 |  |  | 2 |  | 11 |
| chr12.trna1 | Pro | 7 |  |  | 3 |  | 9 |
| chr7.trna38 | Pro | 7 |  |  | 3 |  | 9 |
| chr3.trna59 | Pro | 7 |  |  | 3 |  | 9 |
| chr11.trna34 | Pro | 7 |  |  | 3 |  | 9 |
| chr4.trna46 | Pro | 7 |  |  | 3 |  | 9 |
| chr8.trna38 | Pro | 7 |  |  | 3 |  | 9 |
| chr9.trna20 | Pro | 7 |  |  | 3 |  | 9 |
| chr11.trna29 | Pro | 7 |  |  | 4 |  | 9 |
| chr12.trna58 | Pro | 7 |  |  | 4 |  | 9 |
| chr1.trna93 | Pro | 7 |  |  | 3 |  | 11 |
| chr6.trna8 | Pro | 7 |  |  | 4 |  | 9 |
| chr10.trna54 | Pro | 7 |  |  | 4 |  | 9 |
| chr12.trna62 | Pro | 7 |  |  | 4 |  | 9 |
| chr8.trna15 | Pro | 7 |  |  | 4 |  | 9 |
| chr2.trna24 | Pro | 7 |  |  | 3 |  | 9 |
| chr11.trna26 | Pro | 7 |  |  | 3 |  | 9 |
| chr3.trna65 | Pro | 7 |  |  | 3 |  | 9 |
| chr7.trna25 | Pro | 7 |  |  | 3 |  | 9 |
| chr6.trna5 | Pro | 7 |  |  | 3 |  | 9 |
| chr6.trna19 | Pro | 7 |  |  | 3 |  | 9 |
| chr9.trna23 | Pro | 7 |  |  | 3 |  | 9 |
| chr9.trna30 | Val | 7 |  |  | 3 |  | 11 |
| chr5.trna45 | Val | 7 |  |  | 3 |  | 11 |
| chr10.trna22 | Met | 3 |  |  | 3 |  | 9 |
| chr10.trna57 | Met | 3 |  |  | 3 |  | 9 |
| chr4.trna96 | Met | 3 |  |  | 3 |  | 9 |
| chr1.trna32 | Met | 7 |  |  | 4 |  | 9 |
| chr3.trna44 | Ala | 7 |  |  | 4 |  | 7 |
| chr5.trna59 | Val | 7 |  |  | 4 |  | 7 |
| chr8.trna49 | Tyr | 7 |  |  | 2 |  | 13 |
| chr8.trna43 | Asn | 6 |  |  | 3 |  | 6 |
| chr8.trna51 | Thr | 7 |  |  | 3 |  | 9 |
| chr3.trna27 | Ser | 6 |  |  | Absent |  | Absent |
| chr2.trna20 | Ser | 4 |  |  | 3 |  | 10 |
| chr1.trna68 | Ser | 7 |  |  | 3 |  | 9 |
| chr4.trna71 | Ser | 7 |  |  | 3 |  | 8 |
| chr1.trna2 | Ser | 7 |  |  | 4 |  | 8 |
| chr2.trna73 | Ser | 7 |  |  | 4 |  | 8 |
| chr6.trna12 | Ser | 7 |  |  | 4 |  | 8 |
| chr1.trna64 | Ser | 7 |  |  | 4 |  | 8 |
| chr2.trna43 | Ser | 7 |  |  | 4 |  | 8 |
| chr2.trna47 | Ser | 7 |  |  | 4 |  | 8 |
| chr3.trna40 | Ser | 7 |  |  | 4 |  | 8 |
| chr5.trna11 | Ser | 7 |  |  | 4 |  | 8 |

| chr7.trna42 | Ser | 7 |  |  | 4 |  | 8 |
| --- | --- | --- | --- | --- | --- | --- | --- |
| chr8.trna1 | Ser | 7 |  |  | Absent |  | Absent |
| chr7.trna43 | Ser | 7 |  |  | 3 |  | 4 |
| chr6.trna28 | Ser | 7 |  |  | 3 |  | 10 |
| chr1.trna89 | Ser | 7 |  |  | 3 |  | 10 |
| chr3.trna41 | Ser | 7 |  |  | 3 |  | 10 |
| chr5.trna26 | Ser | 7 |  |  | 3 |  | 10 |
| chr5.trna43 | Ser | 7 |  |  | 3 |  | 10 |
| chr2.trna60 | Ser | 7 |  |  | 3 |  | 10 |
| chr11.trna25 | Ser | 7 |  |  | 3 |  | 11 |
| chr10.trna28 | Ser | 7 |  |  | 3 |  | 11 |
| chr10.trna62 | Ser | 7 |  |  | 3 |  | 11 |
| chr2.trna15 | Ser | 7 |  |  | 3 |  | 11 |
| chr2.trna26 | Ser | 7 |  |  | 3 |  | 11 |
| chr2.trna31 | Ser | 7 |  |  | 3 |  | 11 |
| chr4.trna83 | Ser | 7 |  |  | 3 |  | 11 |
| chr4.trna89 | Ser | 7 |  |  | 3 |  | 11 |
| chr7.trna33 | Ser | 7 |  |  | 3 |  | 11 |
| chr11.trna18 | Ser | 7 |  |  | 3 |  | 10 |
| chr2.trna5 | Ser | 7 |  |  | 3 |  | 10 |
| chr2.trna75 | Ser | 7 |  |  | 3 |  | 10 |
| chr6.trna33 | Ser | 7 |  |  | 3 |  | 10 |
| chr6.trna39 | Ser | 7 |  |  | 3 |  | 10 |
| chr7.trna12 | Ser | 7 |  |  | 3 |  | 10 |
| chr7.trna19 | Ser | 7 |  |  | 3 |  | 10 |
| chr7.trna20 | Ser | 7 |  |  | 3 |  | 10 |
| chr8.trna40 | Ser | 7 |  |  | 3 |  | 10 |
| chr12.trna22 | Ser | 7 |  |  | 3 |  | 11 |
| chr9.trna4 | Ser | 7 |  |  | 3 |  | 11 |
| chr10.trna10 | Ser | 7 |  |  | 3 |  | 11 |
| chr10.trna36 | Ser | 7 |  |  | 3 |  | 11 |
| chr4.trna5 | Ser | 7 |  |  | 3 |  | 11 |
| chr4.trna6 | Ser | 7 |  |  | 3 |  | 11 |
| chr12.trna52 | Ser | 7 |  |  | 3 |  | 11 |
| chr4.trna55 | Ser | 7 |  |  | 3 |  | 11 |
| chr12.trna17 | Ser | 7 |  |  | 4 |  | 9 |
| chr1.trna75 | Ser | 7 |  |  | 4 |  | 9 |
| chr10.trna61 | Ser | 7 |  |  | 4 |  | 9 |
| chr2.trna14 | Ser | 7 |  |  | 4 |  | 9 |
| chr3.trna45 | Ser | 7 |  |  | 4 |  | 9 |
| chr4.trna80 | Ser | 7 |  |  | 4 |  | 9 |
| chr4.trna88 | Ser | 7 |  |  | 4 |  | 9 |
| chr5.trna53 | Ser | 7 |  |  | 4 |  | 9 |
| chr3.trna11 | Ser | 7 |  |  | 3 |  | 10 |

| chr7.trna26 | Ser | 7 |  |  | 3 |  | 10 |
| --- | --- | --- | --- | --- | --- | --- | --- |
| chr1.trna6 | Ser | 7 |  |  | 3 |  | 10 |
| chr11.trna3 | Ser | 7 |  |  | 3 |  | 10 |
| chr12.trna2 | Ser | 7 |  |  | 3 |  | 10 |
| chr5.trna5 | Ser | 7 |  |  | 3 |  | 10 |
| chr4.trna1 | Ser | 7 |  |  | 4 |  | 9 |
| chr2.trna40 | Thr | 7 |  |  | 4 |  | 9 |
| chr1.trna28 | Thr | 7 |  |  | 4 |  | 9 |
| chr1.trna4 | Thr | 7 |  |  | 4 |  | 9 |
| chr10.trna1 | Thr | 7 |  |  | 4 |  | 9 |
| chr12.trna40 | Thr | 7 |  |  | 4 |  | 9 |
| chr12.trna66 | Thr | 7 |  |  | 4 |  | 9 |
| chr3.trna51 | Thr | 7 |  |  | 4 |  | 9 |
| chr3.trna71 | Thr | 7 |  |  | 4 |  | 9 |
| chr7.trna3 | Thr | 7 |  |  | 4 |  | 9 |
| chr12.trna46 | Thr | 7 |  |  | 4 |  | 9 |
| chr5.trna57 | Thr | 7 |  |  | 4 |  | 9 |
| chr2.trna25 | Thr | 7 |  |  | 4 |  | 7 |
| chr4.trna48 | Thr | 7 |  |  | 4 |  | 7 |
| chr6.trna40 | Thr | 7 |  |  | 4 |  | 7 |
| chr9.trna18 | Thr | 7 |  |  | 4 |  | 7 |
| chr6.trna17 | Thr | 7 |  |  | 4 |  | 7 |
| chr1.trna77 | Thr | 7 |  |  | 4 |  | 7 |
| chr3.trna56 | Thr | 7 |  |  | 3 |  | 9 |
| chr1.trna12 | Thr | 7 |  |  | 4 |  | 7 |
| chr10.trna6 | Thr | 7 |  |  | 4 |  | 7 |
| chr2.trna55 | Thr | 7 |  |  | 4 |  | 7 |
| chr4.trna12 | Thr | 7 |  |  | 4 |  | 7 |
| chr4.trna23 | Thr | 7 |  |  | 4 |  | 7 |
| chr4.trna76 | Thr | 7 |  |  | 4 |  | 7 |
| chr12.trna48 | Thr | 7 |  |  | 4 |  | 8 |
| chr12.trna60 | Thr | 7 |  |  | 4 |  | 8 |
| chr1.trna36 | Thr | 7 |  |  | 4 |  | 8 |
| chr10.trna21 | Thr | 7 |  |  | 4 |  | 8 |
| chr10.trna56 | Thr | 7 |  |  | 4 |  | 8 |
| chr12.trna47 | Thr | 7 |  |  | 4 |  | 8 |
| chr12.trna49 | Thr | 7 |  |  | 4 |  | 8 |
| chr4.trna95 | Thr | 7 |  |  | 4 |  | 8 |
| chr4.trna98 | Thr | 7 |  |  | 4 |  | 8 |
| chr10.trna40 | Thr | 7 |  |  | 4 |  | 7 |
| chr2.trna41 | Thr | 7 |  |  | 4 |  | 7 |
| chr3.trna74 | Thr | 7 |  |  | 4 |  | 7 |
| chr8.trna34 | Thr | 7 |  |  | 4 |  | 7 |
| chr3.trna80 | Thr | 7 |  |  | 4 |  | 7 |

| chr9.trna25 | Thr | 7 |  |  | 4 |  | 7 |
| --- | --- | --- | --- | --- | --- | --- | --- |
| chr3.trna61 | Trp | 7 |  |  | 4 |  | 7 |
| chr12.trna63 | Trp | 7 |  |  | 4 |  | 9 |
| chr1.trna94 | Trp | 7 |  |  | 4 |  | 9 |
| chr10.trna55 | Trp | 7 |  |  | 4 |  | 9 |
| chr6.trna9 | Trp | 7 |  |  | 4 |  | 9 |
| chr8.trna16 | Trp | 7 |  |  | 4 |  | 9 |
| chr6.trna32 | Trp | 7 |  |  | 4 |  | 7 |
| chr4.trna70 | Trp | 7 |  |  | 4 |  | 7 |
| chr10.trna47 | Trp | 7 |  |  | 4 |  | 7 |
| chr2.trna29 | Trp | 7 |  |  | 4 |  | 7 |
| chr2.trna44 | Trp | 7 |  |  | 4 |  | 7 |
| chr2.trna46 | Trp | 7 |  |  | 4 |  | 7 |
| chr3.trna17 | Trp | 7 |  |  | 4 |  | 7 |
| chr4.trna57 | Trp | 7 |  |  | 4 |  | 7 |
| chr4.trna69 | Trp | 7 |  |  | 4 |  | 7 |
| chr7.trna32 | Trp | 7 |  |  | 4 |  | 7 |
| chr8.trna6 | Trp | 7 |  |  | 4 |  | 7 |
| chr8.trna7 | Trp | 7 |  |  | 4 |  | 7 |
| chr12.trna19 | Tyr | 7 |  |  | 4 |  | 9 |
| chr2.trna4 | Tyr | 7 |  |  | 4 |  | 9 |
| chr4.trna14 | Tyr | 7 |  |  | 4 |  | 9 |
| chr4.trna25 | Tyr | 7 |  |  | 4 |  | 9 |
| chr2.trna57 | Tyr | 7 |  |  | 4 |  | 9 |
| chr1.trna34 | Tyr | 7 |  |  | 2 |  | 13 |
| chr1.trna79 | Tyr | 7 |  |  | 4 |  | 9 |
| chr10.trna8 | Tyr | 7 |  |  | 4 |  | 9 |
| chr10.trna48 | Tyr | 7 |  |  | 4 |  | 8 |
| chr9.trna31 | Tyr | 7 |  |  | 4 |  | 8 |
| chr5.trna51 | Tyr | 7 |  |  | 4 |  | 8 |
| chr8.trna45 | Tyr | 7 |  |  | 4 |  | 8 |
| chr8.trna21 | Tyr | 7 |  |  | 4 |  | 8 |
| chr8.trna22 | Tyr | 7 |  |  | 4 |  | 8 |
| chr3.trna22 | Tyr | 7 |  |  | 4 |  | 8 |
| chr4.trna58 | Tyr | 7 |  |  | 4 |  | 8 |
| chr2.trna30 | Tyr | 7 |  |  | 4 |  | 8 |
| chr1.trna82 | Tyr | 7 |  |  | 4 |  | 8 |
| chr4.trna52 | Tyr | 7 |  |  | 4 |  | 8 |
| chr3.trna3 | Tyr | 7 |  |  | 4 |  | 8 |
| chr9.trna6 | Tyr | 7 |  |  | 4 |  | 8 |
| chr3.trna48 | Val | 7 |  |  | 3 |  | 11 |
| chr3.trna49 | Val | 7 |  |  | 3 |  | 11 |
| chr12.trna42 | Val | 7 |  |  | 3 |  | 11 |
| chr4.trna50 | Val | 7 |  |  | 3 |  | 11 |

| chr5.trna13 | Val | 7 |  |  | 3 |  | 11 |
| --- | --- | --- | --- | --- | --- | --- | --- |
| chr6.trna6 | Val | 7 |  |  | 3 |  | 11 |
| chr8.trna9 | Val | 7 |  |  | 3 |  | 11 |
| chr3.trna42 | Val | 7 |  |  | 3 |  | 11 |
| chr4.trna30 | Val | 7 |  |  | 3 |  | 11 |
| chr5.trna28 | Val | 7 |  |  | 3 |  | 11 |
| chr1.trna51 | Val | 7 |  |  | 3 |  | 11 |
| chr1.trna65 | Val | 7 |  |  | 3 |  | 11 |
| chr3.trna47 | Val | 7 |  |  | 3 |  | 11 |
| chr9.trna16 | Val | 7 |  |  | 3 |  | 11 |
| chr8.trna31 | Val | 7 |  |  | 3 |  | 11 |
| chr8.trna32 | Val | 7 |  |  | 3 |  | 11 |
| chr9.trna12 | Val | 7 |  |  | 3 |  | 11 |
| chr1.trna69 | Val | 7 |  |  | 3 |  | 11 |
| chr8.trna36 | Val | 7 |  |  | 3 |  | 11 |
| chr1.trna44 | Val | 7 |  |  | 3 |  | 11 |
| chr11.trna13 | Val | 7 |  |  | 3 |  | 11 |
| chr11.trna5 | Val | 7 |  |  | 3 |  | 11 |
| chr12.trna4 | Val | 7 |  |  | 3 |  | 11 |
| chr3.trna28 | Val | 7 |  |  | 3 |  | 11 |
| chr4.trna66 | Val | 7 |  |  | 3 |  | 11 |
| chr9.trna35 | Val | 7 |  |  | 3 |  | 11 |
| chr9.trna36 | Val | 7 |  |  | 3 |  | 11 |
| chr4.trna10 | Val | 7 |  |  | 3 |  | 11 |
| chr4.trna20 | Val | 7 |  |  | 4 |  | 7 |
| chr4.trna92 | Val | 7 |  |  | 4 |  | 7 |
| chr3.trna39 | Val | 7 |  |  | 4 |  | 7 |
| chr10.trna14 | Val | 7 |  |  | 4 |  | 7 |
| chr10.trna19 | Val | 7 |  |  | 4 |  | 7 |
| chr10.trna65 | Val | 7 |  |  | 4 |  | 7 |
| chr2.trna11 | Val | 7 |  |  | 4 |  | 7 |
| chr2.trna77 | Val | 7 |  |  | 4 |  | 7 |
| chr4.trna16 | Val | 7 |  |  | 4 |  | 7 |
| chr4.trna18 | Val | 7 |  |  | 4 |  | 7 |
| chr4.trna27 | Val | 7 |  |  | 4 |  | 7 |
| chr4.trna3 | Val | 7 |  |  | 4 |  | 7 |
| chr5.trna54 | Val | 7 |  |  | 4 |  | 7 |
| chr7.trna41 | Val | 7 |  |  | 4 |  | 7 |
| chr8.trna47 | Val | 7 |  |  | 4 |  | 7 |
| chr1.trna23 | Val | 7 |  |  | 3 |  | 10 |
| chr4.trna61 | Val | 7 |  |  | 3 |  | 10 |
| chr5.trna30 | Val | 7 |  |  | 3 |  | 10 |
| chr5.trna33 | Val | 7 |  |  | 3 |  | 10 |
