# Supplementary material for: Analyses of Genomic tRNA Reveal Presence of Novel tRNAs in Oryza sativa
Source: Front Genet. 2017 Jun 30;8:90. doi: 10.3389/fgene.2017.00090 (PMC5492330; doi:10.3389/fgene.2017.00090)
Supplement: Supplementary file 3 [file Image1.PDF]

Supplementary Figure 1.

Conserved region of O. sativa tRNA. AA; accepter arm, DA; D-arm, DL; D-loop, ACA; anticodon arm, ACL; anti-codon loop, VR; variable region, TA; T arm, TL; T-loop.

```
                   AA       DA   DL          DA   ACA      ACL      ACA VR   TA     TL     TA     AA
Ala_tRNA18  GGGGATGTGGCTCAGATGGT --AGAGCGCTCGCTTAGCA- -TGTGAGAGGTACGGGGATCGATACCCCGCAT-CTACA
Ala_tRNA12  GGGGATGTAGCTCAGATGGT --AGAGCGCTCGCTTAGCA- -TGCGAGAAGTACGGGGATCGATACCCCGCAT-CTCCA
Ala_tRNA32  GGGGGTGTAGCTCAGATGGT --AGAGCGCTCGCTTAGCA- -TGCGAGAGGTACGGGGATCGATACCCCGCAT-CTCCA
Ala_tRNA62  GGGGATGTAGCTCAGATGGT --AGAGCGCTCGCTTAGCA- -TGCGAGAGGTACGGGGATCGATACCCCGCAT-CTCCA
Ala_tRNA26  GGGGATGTAGCTCAGATGGT --AGAGCGCTCGCTTAGCA- -TGCGAGAGGTACGGGGATCGATACCCCGCAT-CTCCA
Ala_tRNA39  GGGGATGTAGCTCAGATGGT --AGAGCGCTCGCTTAGCA- -TGCGAGAGGTACGGGGATCGATACCCCGCAT-CTCCA
Ala_tRNA14  GGGGATGTAGCTCAGATGGT --AGAGCGCTCGCTTAGCA- -TGCGAGAGGTACGGGGATCGATACCCCGCAT-CTCCA
Ala_tRNA69  GGGGATGTAGCTCAGATGGT --AGAGCGCTCGCTTAGCA- -TGCGAGAGGTACGGGGATCGATACCCCGCAT-CTCCA
Ala_tRNA50  GGGGATGTAGCTCAGATGGT --AGAGCGCTCGCTTAGCA- -TGCGAGAGGTACGGGGATCGATACCCCGCAT-CTCCA
Ala_tRNA63  GGGGATGTAGCTCAGATGGT --AGAGCGCTCGCTTAGCA- -TGCGAGAGGTACGGGGATCGATACCCCGCAT-CTCCA
Ala_tRNA68  GGGGATGTAGCTCAGATGGT --AGAGCGCTCGCTTAGCA- -TGCGAGAGGTACGGGGATCGATACCCCGCAT-CTCCA
Ala_tRNA22  GGGGATGTAGCTCAGATGGT --AGAGCGCTCGCTTAGCA- -TGCGAGAGGTACGGGGATCGATACCCCGCAT-CTCCA
Ala_tRNA20  GGGGATGTAGCTCAGATGGT --AGAGCGCTCGCTTAGCA- -TGCGAGAGGTACGGGGATCGATACCCCGCAT-CTCCA
Ala_tRNA19  GGGGATGTAGCTCAGATGGT --AGAGCGCTCGCTTAGCA- -TGCGAGAGGTACGGGGATCGATACCCCGCAT-CTCCA
Ala_tRNA24  GGGGATGTAGCTCAGATGGT --AGAGCGCTCGCTTAGCA- -TGCGAGAGGTACGGGGATCGATACCCCGCAT-CTCCA
Ala_tRNA37  GGGGATGTAGCTCAGATGGT --AGAGCGCTCGCTTAGCA- -TGCGAGAGGTACGGGGATCGATACCCCGCAT-CTCCA
Ala_tRNA35  GGGGATGTAGCTCAGATGGT --AGAGCGCTCGCTTAGCA- -TGCGAGAGGTACGGGGATCGATACCCCGCAT-CTCCA
Ala_tRNA16  GGGGATGTAGCTCAGATGGT --AGAGCGCTCGCTTAGCA- -TGCGAGAGGTACGGGGATCGATACCCCGCAT-CTCCA
Ala_tRNA21  GGGGATGTAGCTCAGATGGT --AGAGCGCTCGCTTAGCA- -TGCGAGAGGTACGGGGATCGATACCCCGCAT-CTCCA
Ala_tRNA31  GGGGATGTAGCTCAGATGGT --AGAGCGCTCGCTTAGCA- -TGCGAGAGGTACGGGGATCGATACCCCGCAT-CTCCA
Ala_tRNA53  GGGGGTGTAGCTCATATGGT --AGAGCGCTCGCTTCGCA- -TGCGAGAGGCACGGTGTTCGATTCCCCGCAC-CTCCA
Ala_tRNA3_  GGGGGTGTAGCTCATATGGT --AGAGCGCTCGCTTCGCA- -TGCGAGAGGCACGGGGTTCGATTCCCCGCAC-CTCCA
Ala_tRNA9_  GGGGGTGTAGCTCATATGGT --AGAGCGCTCGCTTCGCA- -TGCGAGAGGCACGGGGTTCGATTCCCCGCAC-CTCCA
Ala_tRNA60  GGGGGTGTAGCTCATATGGT --AGAGCGCTCGCTTCGCA- -TGCGAGAGGCACGGGGTTCGATTCCCCGCAC-CTCCA
Ala_tRNA29  GGGGGTGTAGCTCATATGGT --AGAGCGCTCGCTTCGCA- -TGCGAGAGGCACGGGGTTCGATTCCCCGCAC-CTCCA
Ala_tRNA16  GGGGACGTAGCTCATATGGT --AGAGCGCTCGCTTCGCA- -TGCGAGAGGCACGGGGTTCGATTCCCCGCGT-CTCCA
Ala_tRNA19  GGGGACGTAGCTCATATGGT --AGAGCGCTCGCTTCGCA- -TGCGAGAGGCACGGGGTTCGATTCCCCGCGT-CTCCA
Ala_tRNA36  GGGGACGTAGCTCATATGGT --AGAGCGCTCGCTTCGCA- -TGCGAGAGGCACGGGGTTCGATTCCCCGCGT-CTCCA
Ala_tRNA37  GGGGACGTAGCTCATATGGT --AGAGCGCTCGCTTCGCA- -TGCGAGAGGCACGGGGTTCGATTCCCCGCGT-CTCCA
Ala_tRNA26  GGGGACGTAGCTCATATGGT --AGAGCGCTCGCTTCGCA- -TGCGAGAGGCACGGGGTTCGATTCCCCGCGT-CTCCA
Ala_tRNA14  GGGGACGTAGCTCATATGGT --AGAGCGCTCGCTTCGCA- -TGCGAGAGGCACGGGGTTCGATTCCCCGCGT-CTCCA
Ala_tRNA48  GGGGATGTAGCTCAAATGGT --AGAGCGCTCGCTTTGCA- -TGCGAGAGGCACGGGGTTCGATCCCCCGCAT-CTCCA
Ala_tRNA29  GGGGATGTAGCTCAAATGGT --AGAGCGCTCGCTTTGCA- -TGCGAGAGGCACGGGGTTCGATCCCCCGCAT-CTCCA
Ala_tRNA30  GGGGATGTAGCTCAAATGGT --AGAGCGCTCGCTTTGCA- -TGCGAGAGGCACGGGGTTCGATCCCCCGCAT-CTCCA
Ala_tRNA48  GGGGATGTAGCTCAAATGGT --AGAGCGCTCGCTTTGCA- -TGCGAGAGGCACGGGGTTCGATCCCCCGCAT-CTCCA
Ala_tRNA45  GGGGATGTAGCTCAAATGGT --AGAGCGCTCGCTTTGCA- -TGCGAGAGGCACGGGGTTCGATCCCCCGCAT-CTCCA
Ala_tRNA23  GGGGATGTAGCTCAAATGGT --AGAGCGCTCGCTTTGCA- -TGCGAGAGGCACGGGGTTCGATCCCCCGCAT-CTCCA
Ala_tRNA33  GGGGATGTAGCTCAAATGGT --AGAGCGCTCGCTTTGCA- -TGCGAGAGGCACGGGGTTCGATCCCCCGCAT-CTCCA
Ala_tRNA39  GGGGATGTAGCTCAAATGGT --AGAGCGCTCGCTTTGCA- -TGCGAGAGGCACGGGGTTCGATCCCCCGCAT-CTCCA
Ala_tRNA70  GGGGATGTAGCTCAAATGGT --AGAGCGCTCGCTTTGCA- -TGCGAGAGGCACGGGGTTCGATCCCCCGCAT-CTCCA
Ala_tRNA9_  GGGGATGTAGCTCAAATGGT --AGAGCGCTCGCTTTGCA- -TGCGAGAGGCACGGGGTTCGATCCCCCGCAT-CTCCA
Ala_tRNA1_  CGGGGTGTAGCTCATATGGT --AGAGCGCTCGCTTCGCA- -TGCGAGGTGCACGGGGTTCGATTCCGGCAC-CTCCA
Ala_tRNA44  GCGCTCGTAGCGCAGTGGCA --GGGTCGCTGGCA-CGCA- -CCCCATTGGCCCGG-GTTCGATCCTTGTCGAGCGCC
Ala_tRNA43  GTCAAGATGGCCGAGTTGGT CTAAGGCGCCAGTTTGGCAA ATTTCATTGGCATGG-GTTCGAATCCCATTCTTGACA
```

```
Arg_tRNA64  GGGCCTGTAGCTTAGAGGATTAGAGCACGTGGCTACGA-ACCACGGTGTCGGGGGTTCGATTCCCTCC-TCGCCCA
Arg_tRNA38  GGGCCTGTAGCTCAGAGGATTAGAGCACGTGGCTACGA-ACCACGGTGTCGGGGGTTCGAATCCCTCC-TCGCCCA
Arg_tRNA15  GGGCCTGTAGCTCAGAGGATTAGAGCACGTGGCTACGA-ACCACGGTGTCGGGGGTTCGAATCCCTCC-TCGCCCA
Arg_tRNA20  GGGCCTGTAGCTCAGAGGATTAGAGCACGTGGCTACGA-ACCACGGTGTCGGGGGTTCGAATCCCTCC-TCGCCCA
Arg_tRNA30  GGGCCTGTAGCTCAGAGGATTAGAGCACGTGGCTACGA-ACCACGGTGTCGGGGGTTCGAATCCCTCC-TCGCCCA
Arg_tRNA64  GGGCCTGTAGCTCAGAGGATTAGAGCACGTGGCTACGA-ACCACGGTGTCGGGGGTTCGAATCCCTCC-TCGCCCA
Arg_tRNA17  GGGCCTGTAGCTCAGAGGATTAGAGCACGTGGCTACGA-ACCACGGTGTCGGGGGTTCGAATCCCTCC-TCGCCCA
Arg_tRNA21  GGGCCTGTAGCTCAGAGGATTAGAGCACGTGGCTACGA-ACCACGGTGTCGGGGGTTCGAATCCCTCC-TCGCCCA
Arg_tRNA28  GGGCCTGTAGCTCAGAGGATTAGAGCACGTGGCTACGA-ACCACGGTGTCGGGGGTTCGAATCCCTCC-TCGCCCA
Arg_tRNA4_  GGGCCTGTAGCTCAGAGGATTAGAGCACGTGGCTACGA-ACCACGGTGTCGGGGGTTCGAATCCCTCC-TCGCCCA
Arg_tRNA48  GGGCCTGTAGCTCAGAGGATTAGAGCACGTGGCTACGA-ACCACGGTGTCGGGGGTTCGAATCCCTCC-TCGCCCA
Arg_tRNA41  GATCATATAGCGAAGTGGAT-ATCGCGTTAGATTCCGA-ATCTAAAAGTCGTGGGTTCGAATCCCACT-ATGATCG
Arg_tRNA40  GGTCGCATAGCGAAGTGGAT-ATCGCGTTAGATTCCGA-ATCTAAAAGTCGTGGGTTCGAATCCCACT-GCGATCA
Arg_tRNA3_  GACCGCATAGCGCAGTGGATTAGCGCGTCTGACTTCGG-ATCAGAAAGTCGTGGGTTCGACTCCCACT-GTGGTCG
Arg_tRNA52  GACCGCATAGCGCAGTGGATTAGCGCGTCTGACTTCGG-ATCAGAAGGTCGTGGGTTCGACTCCCACT-GTGGTCG
Arg_tRNA6_  GACCGCATAGCGCAGTGGATTAGCGCGTCTGACTTCGG-ATCAGAAGGTCGTGGGTTCGACTCCCACT-GTGGTCG
Arg_tRNA4_  GACCGCATAGCGCAGTGGATTAGCGCGTCTGACTTCGG-ATCAGAAGGTCGTGGGTTCGACTCCCACT-GTGGTCG
Arg_tRNA92    ACGTATAGCTCAGTGGAT-AGAGCGTCTGTTTCCTA-AGCAGAAGGCCGTAGGTTCGACCCCTACC-TGGCGCG
Arg_tRNA9_  GCGCCTGTAGCTCAGTGGAT-AGAGCGTCTGTTTCCTA-AGCA--AGGTCGTAGGTTCGACCCCTACC-TGGCGCG
Arg_tRNA91  GCGCCTGTAGCTCAGTGGAT-AGAGCGTCTGTTTCCTA-AGCAGAAGGCCGTAGGTTCGACCCCTACC-TGGCGCG
Arg_tRNA27  GCGCCTGTAGCTCAGTGGAT-AGAGCGTCTGTTTCCTA-AGCAGAAGGCCGTAGGTTCGACCCCTACC-TGGCGCG
Arg_tRNA41  GCGCCTGTAGCTCAGTGGAT-AGAGCGTCTGTTTCCTA-AGCAGAAGGCCGTAGGTTCGACCCCTACC-TGGCGCG
Arg_tRNA67  GCGCCTGTAGCTCAGTGGAT-AGAGCGTCTGTTTCCTA-AGCAGAAGGCCGTAGGTTCGACCCCTACC-TGGCGCG
Arg_tRNA57  GCGCCTGTAGCTCAGTGGAT-AGAGCGTCTGTTTCCTA-AGCAGAAGGCCGTAGGTTCGACCCCTACC-TGGCGCG
Arg_tRNA27  GCGCCTGTAGCTCAGTGGAT-AGAGCGTCTGTTTCCTA-AGCAGAAGGCCGTAGGTTCGACCCCTACC-TGGCGCG
Arg_tRNA2_  GCGCCTGTAGCTCAGTGGAT-AGAGCGTCTGTTTCCTA-AGCAGAAGGTCGTAGGTTCGACCCCTACC-TGGCGCG
Arg_tRNA72  GCGCCTGTAGCTCAGTGGAT-AGAGCGTCTGTTTCCTA-AGCAGAAGGTCGTAGGTTCGACCCCTACC-TGGCGCG
Arg_tRNA54  GCGCCTGTAGCTCAGTGGAT-AGAGCGTTTGTTTCCTA-AACAAAAAGTCGAAGGTTCGAACCCTTCC-TGGCGCG
Arg_tRNA31  GACTCCGTGGCCCAATGGATAAG-GAGCTGGTCTACGG-AACCAGAGATTCTGGGTTCGATCCCCAGC-GGAGTCG
Arg_tRNA16  GACTCCATGGCCCAATGGATAAG-GCGCTGGTCTACGG-AACCAGAGATTCTGGGTTCGATCCCCAGT-GGAGTCG
Arg_tRNA21  GACTCCATGGCCCAATGGATAAG-GCGCTGGTCTACGG-AACCAGAGATTCTGGGTTCGATCCCCAGT-GGAGTCG
Arg_tRNA4_  GACTCCATGGCCCAATGGATAAG-GCGCTGGTCTACGG-AACCAGAGATTCTGGGTTCGATCCCCAGT-GGAGTCG
Arg_tRNA38  GACTCCATGGCCCAATGGATAAG-GCGCTGGTCTACGG-AACCAGAGATTCTGGGTTCGATCCCCAGT-GGAGTCG
Arg_tRNA20  GACTCCGTGGCCCAATGGATAAG-GCGCTGGTCTACGG-AACCAGAGATTCTGGGTTCGATCCCCAGC-GGAGTCG
Arg_tRNA27  GACTCCGTGGCCCAATGGATAAG-GCGCTGGTCTACGG-AACCAGAGATTCTGGGTTCGATCCCCAGC-GGAGTCG
Arg_tRNA29  GACTCCGTGGCCCAATGGATAAG-GCGCTGGTCTACGG-AACCAGAGATTCTGGGTTCGATCCCCAGC-GGAGTCG
Arg_tRNA36  GACTCCGTGGCCCAATGGATAAG-GCGCTGGTCTACGG-AACCAGAGATTCTGGGTTCGATCCCCAGC-GGAGTCG
Arg_tRNA44  GACTCCGTGGCCCAATGGATAAG-GCGCTGGTCTACGG-AACCAGAGATTCTGGGTTCGATCCCCAGC-GGAGTCG
Arg_tRNA39  GACTCCGTGGCCCAATGGATAAG-GCGCTGGTCTACGG-AACCAGAGATTCTGGGTTCGATCCCCAGC-GGAGTCG
Arg_tRNA33  GATTCCATGGCCCAATGGATAAG-GCGCTGGTCTACGG-AACCAGAGATTCTGGGTTCGATCCCCAGT-GGAATCG
Arg_tRNA25  GACCGCGTGGCCTAATGGATAAG-GCGCTCGCCTCCGG-AGCGGGAGATTGTGTGTTCGAGTCCCAGC-GTGGTCG
Arg_tRNA59  GACCGCGTGGCCTAATGGATAAG-GCGCTCGCCTCCGG-AGCGGGAGATTGTGGGTTCGAGTCCCATC-GTGGTCG
Arg_tRNA10  GACCGCGTGGCCTAATGGATAAG-GCGCTCGCCTCCGG-AGCGGGAGATTGTGGGTTCGAGTCCCATC-GTGGTCG
Arg_tRNA39  GACCGCGTGGCCTAATGGATAAG-GCGCTCGCCTCCGG-AGCGGGAGATTGTGGGTTCGAGTCCCATC-GTGGTCG
Arg_tRNA21  GACCGCGTGGCCTAATGGATAAG-GCGCTCGCCTCCGG-AGCGGGAGATTGTGGGTTCGAGTCCCATC-GTGGTCG
Arg_tRNA43  GGTCGCGTGGCCTAATGGATAAG-GCGCTCGCCTCCGG-AGCGGGAGATTGTGGGTTCGAGTCCCACC-GTGATCG
Arg_tRNA11  GCGCCCATGGCCTAATGGATAAG-GCGTCTGACTTCTA-ATCAGGCGATTGTGGGTTCGAGTCCCACT-GGGCGTG
Arg_tRNA17  GCGCCCATGGCCTAATGGATAAG-GCGTCTGACTTCTA-ATCAGGCGATTGTGGGTTCGAGTCCCACT-GGGCGTG
Arg_tRNA50  GCGCCCATGGCCTAATGGATAAG-GCGTCTGACTTCTA-ATCAGGCGATTGTGGGTTCGAGTCCCACT-GGGCGTG
Arg_tRNA19  GCGCCCATGGCCTAATGGATAAG-GCGTCTGACTTCTA-ATCAGGCGATTGTGGGTTCGAGTCCCACT-GGGCGTG
Arg_tRNA57  GCGCCCGTGGCCTAATGGATAAG-GCGTCTGACTTCTA-ATCAGGCGATTGTGGGTTCGAGTCCCACC-GGGCGTG
Arg_tRNA8_  GCGCCTGTGGCCTAATGGATAAG-GCGTCTGACTTCTA-ATCAGACGATTGTGGGTTCGAGTCCCACC-AGGCGTG
Arg_tRNA6_  GCGCCTGTGGCCTAATGGATAAG-GCGTCTGACTTCTA-ATCAGACGATTGTGGGTTCGAGTCCCACC-AGGCGTG
Arg_tRNA55  GCGTCCATTGTCTAATGGAT-AGGACAGAGGTCTTCTA-AACCTTTGGTA-TATGTTCAAATCCTATTTGGACGCA
Arg_tRNA58  GTGTCCATTGTCTAATGGAT-AGGACAGAGGTCTTCTA-AACCTTTGGTA-TAGGTTCAAATCCTATT-GGACGCA
Arg_tRNA23  GCGTCCATTGTCTAATGGAT-AGGACAGAGGTCTTCTA-AACCTTTGGTA-TAGGTTCAAATCCTATT-GGACGCA
Arg_tRNA3_  GCGTCCATTGTCTAATGGAT-AGGACAGAGGTCTTCTA-AACCTTTGGTA-TAGGTTCAAATCCTATT-GGACGCA
```

```
             AA   DA   DL   DA   ACA   ACL   ACA   VR   TA   TL   TA   AA
Asn_tRNA76  TCCTCAGTAGCTCAGTGG--TAGAGCGGTCGGTTGTTAACTGACTGGTCGTAGGTTCGAATTCTACTTGGGGAG
Asn_tRNA40  TCCTCAGTAGCTCAGTGG--TAGAGCGGTCGGCTGTTAATTGACTGGTCGTAGGTTCGAATCCTACTTGGGGAG
Asn_tRNA18  TCCTCAGTAGCTCAGTGG--TAGAGCGGTCGGCTGTTAACTGACTGGTCGTAGGTTCGAATCCTACTTGGGGAG
Asn_tRNA73  TCCTCAGTAGCTCAGTGG--TAGAGCGGTCGGCTGTTAACTGACTGGTCGTAGGTTCGAATCCTACTTGGGGAG
Asn_tRNA38  TCCTCAGTAGCTCAGTGG--TAGAGCGGTCGGCTGTTAACTGACTGGTCGTAGGTTCGAATCCTACTTGGGGAG
Asn_tRNA51  TCCTCAGTAGCTCAGTGG--TAGAGCGGTCGGCTGTTAACTGACTGGTCGTAGGTTCGAATCCTACTTGGGGAG
Asn_tRNA12  TCCTCAGTAGCTCAGTGG--TAGAGCGGTCGGCTGTTAACTGACTGGTCGTAGGTTCGAATCCTACTTGGGGAG
Asn_tRNA99  TCCTCAGTAGCTCAGTGG--TAGAGCGGTCGGCTGTTAACTGACTGGTCGTAGGTTCGAATCCTACTTGGGGAG
Asn_tRNA77  TCCTCAGTAGCTCAGTGG--TAGAGCGGTCGGCTGTTAACTGACTGGTCGTAGGTTCGAATCCTACTTGGGGAG
Asn_tRNA53  TCCTCAGTAGCTCAGTGG--TAGAGCGGTCGGCTGTTAACTGACTGGTCGTAGGTTCGAATCCTACTTGGGGAG
Asn_tRNA15  TCCTCAGTAGCTCAGTGG--TAGAGCGGTCGGCTGTTAACTGACTGGTCGTAGGTTCGAATCCTACTTGGGGAG
Asn_tRNA25  TCCTCAGTAGCTCAGTGG--TAGAGCGGTCGGCTGTTAACTGACTGGTCGTAGGTTCGAATCCTACTTGGGGAG
Asn_tRNA12  TCCTCAGTAGCTCAGTGG--TAGAGCGGTCGGCTGTTAACTGACTGGTCGTAGGTTCGAATCCTACTTGGGGAG
Asn_tRNA65  TCCTCGGTAGCTCAGTGG--TAGAGCGGTCAGCTGTTAACTGACTGGTCGTAGGTTCGAATCCTACTTGGGGAG
Asn_tRNA19  GCTGTAATAGCTCAGTTGGTTAGAGCGTGTGGCTGTTAACCACAAGGTCGGAGGTTCAAGCCCTCCTTCTAGCG
Asn_tRNA26  GCTGGAATAGCTCAGTTGGTTAGAGCGTGTGGCTGTTAACCACAAGGTCGGAGGTTCAAGCCCTCCTTCTAACG
Asn_tRNA14  GCTGGAATAGCTCAGTTGGTTAGAGCGTGTGGCTGTTAACCACAAGGTCGGAGGTTCAAGCCCTCCTTCTAGCG
Asn_tRNA15  GCTGGAATAGCTCAGTTGGTTAGAGCGTGTGGCTGTTAACCACAAGGTCGGAGGTTCAAGCCCTCCTTCTAGCG
Asn_tRNA17  GCTGGAATAGCTCAGTTGGTTAGAGCGTGTGGCTGTTAACCACAAGGTCGGAGGTTCAAGCCCTCCTTCTAGCG
Asn_tRNA56  GCTGGAATAGCTCAGTTGGTTAGAGCGTGTGGCTGTTAACCACAAGGTCGGAGGTTCAAGCCCTCCTTCTAGCG
Asn_tRNA81  GCTGGAATAGCTCAGTTGGTTAGAGCGTGTGGCTGTTAACCACAAGGTCGGAGGTTCAAGCCCTCCTTCTAGCG
Asn_tRNA41  GCTGGAATAGCTCAGTTGGTTAGAGCGTGTGGCTGTTAACCACAAGGTCGGAGGTTCAAGCCCTCCTTCTAGCG
Asn_tRNA47  GCTGGAGTAGCTCAGTTGGTTAGAGCGTGTGGCTGTTAACCACAAGGTCGGAGGTTCAAGCCCTCCCTCTAGCG
Asn_tRNA48  GCTGGAGTAGCTCAGTTGGTTAGAGCGTGTGGCTGTTAACCACAAGGTCGGAGGTTCAAGCCCTCCCTCTAGCG
Asn_tRNA58  GCTGGAGTAGCTCAGTTGGTTAGAGCGTGTGGCTGTTAACCACAAGGTCGGAGGTTCAAGCCCTCCCTCTAGCG
Asn_tRNA14  GCTGGAGTAGCTCAGATGGTTAGAGCGTGTGGCTGTTAACCACAAGGTCGAAGGTTCAAGCCCTTCCTCTAGCG
Asn_tRNA78  GCTGGAATAGCTCAGTTGGCCAGAGCGTATGGCTGTTAACCATAAGGTCGGAGGTTCGAGCCCTCCTTCTAGCG
Asn_tRNA24  GCTGGAATAGCTCAGTTGGCTAGAGCGTATGGCTGTTAACCATAAGGTCGGAGGTTCGAGCCCTCCTTCTAGCG
Asn_tRNA35  GCTGGAATAGCTCAGTTGGCTAGAGCGTATGGCTGTTAACCATAAGGTCGGAGGTTCGAGCCCTCCTTCTAGCG
Asn_tRNA43  ACTTGTGTGGCTTAGTGG--TGGGAACATAGGCTGTTGTTCTTGTGGTTGTGGGTTCGAATCCCCCGAAGAGCA
```

```
              AA      DA   DL      DA  ACA  ACL  ACA VR   TA  TL   TA   AA
Asp_tRNA9_  GGGATTGTAGTTCAATTGGTCAGAGCACCGCCCTATCAAGGCGGAA-GCTGCGGGTTCGAGCCCCGTCAGTCCCG
Asp_tRNA58  GGGATTGTAGTTCAATTGGTCAGAGCACCGCCCTGTCAAGGCGGAA-GCTGCGGGTTCGAGCCCCGTCAGTCCCG
Asp_tRNA15  GGGATTGTAGTTCAATTGGTCAGAGCACCGCCCTGTCAAGGCGGAA-GCTGCGGGTTCGAGCCCCGTCAGTCCCG
Asp_tRNA26  GGGATTGTAGTTCAATTGGTCAGAGCACCGCCCTGTCAAGGCGGAA-GCTGCGGGTTCGAGCCCCGTCAGTCCCG
Asp_tRNA16  GGGATTGTAGTTCAATTGGTCAGAGCACCGCCCTGTCAAGGCGGAA-CCTGCGGGTTCGAGCCCCGTCAGTCCCG
Asp_tRNA28  GGGATTGTAGTTCAATTGGTCAGAGCACCGCCCTGTCAAGGCGGAA-GCTGTGGGTTCGAGCCCCGTCAGTCCCG
Asp_tRNA80    GATTGTAGTTCAATTGGTCAGAGCACCGCCCTGTCAAGGCGGAA-GCTGCGGGTTCGAGCCCCGTCAGTCA
Asp_tRNA54  GGGGAAATAGCTCAGTTGGTTAGAGTGCTGGTCTGTCACGCCAGAA-GTCGCGGGTTCGAACCCCGTTTTCCCCG
Asp_tRNA6_  GTCGTTGTAGTATAGT-GGTGAGTATTCCCGCCTGTCACGCAGGTGTGACCCGGGTTCGATCCCCGGCAACGGCG
Asp_tRNA23  GTCGTTGTAGTGTAGT-GGTGAGTATTCCCGCCTGTCACGCGGGTG--ACCCGGGTTCGATCCCCGGCAACGGGG
Asp_tRNA16  ATCGTTGTAGTATAGT-GGTGAGTATTCCCGCCTGTCACGCGGGTG--ACCCGGGTTCGATCCCCGGCAACGGCG
Asp_tRNA5_  GTCGTTGTAGTATAGT-GGTGAGTATTCCCGCCTGTCACGCGGGTG--ACCCGGGTTCGATCCCCGGCAACGGCG
Asp_tRNA81  GTCGTTGTAGTATAGT-GGTGAGTATTCCCGCCTGTCACGCGGGTG--ACCCGGGTTCGATCCCCGGCAACGGCG
Asp_tRNA84  GTCGTTGTAGTATAGT-GGTGAGTATTCCCGCCTGTCACGCGGGTG--ACCCGGGTTCGATCCCCGGCAACGGCG
Asp_tRNA87  GTCGTTGTAGTATAGT-GGTGAGTATTCCCGCCTGTCACGCGGGTG--ACCCGGGTTCGATCCCCGGCAACGGCG
Asp_tRNA49  GTCGTTGTAGTATAGT-GGTGAGTATTCCCGCCTGTCACGCGGGTG--ACCCGGGTTCGATCCCCGGCAACGGCG
Asp_tRNA19  GTCGTTGTAGTATAGT-GGTGAGTATTCCCGCCTGTCACGCGGGTG--ACCCGGGTTCGATCCCCGGCAACGGCG
Asp_tRNA20  GTCGTTGTAGTATAGT-GGTGAGTATTCCCGCCTGTCACGCGGGTG--ACCCGGGTTCGATCCCCGGCAACGGCG
Asp_tRNA76  GTCGTTGTAGTATAGT-GGTGAGTATTCCCGCCTGTCACGCGGGTG--ACCCGGGTTCGATCCCCGGCAACGGCG
Asp_tRNA2_  GTCGTTGTAGTATAGT-GGTGAGTATTCCCGCCTGTCACGCGGGTG--ACCCGGGTTCGATCCCCGGCAACGGCG
Asp_tRNA15  GTCGTTGTAGTATAGT-GGTGAGTATTCCCGCCTGTCACGCGGGTG--ACCCGGGTTCGATCCCCGGCAACGGCG
Asp_tRNA17  GTCGTTGTAGTATAGT-GGTGAGTATTCCCGCCTGTCACGCGGGTG--ACCCGGGTTCGATCCCCGGCAACGGCG
Asp_tRNA18  GTCGTTGTAGTATAGT-GGTGAGTATTCCCGCCTGTCACGCGGGTG--ACCCGGGTTCGATCCCCGGCAACGGCG
Asp_tRNA19  GTCGTTGTAGTATAGT-GGTGAGTATTCCCGCCTGTCACGCGGGTG--ACCCGGGTTCGATCCCCGGCAACGGCG
Asp_tRNA25  GTCGTTGTAGTATAGT-GGTGAGTATTCCCGCCTGTCACGCGGGTG--ACCCGGGTTCGATCCCCGGCAACGGCG
Asp_tRNA2_  GTCGTTGTAGTATAGT-GGTGAGTATTCCCGCCTGTCACGCGGGTG--ACCCGGGTTCGATCCCCGGCAACGGCG
Asp_tRNA38  GTCGTTGTAGTATAGT-GGTGAGTATTCCCGCCTGTCACGCGGGTG--ACCCGGGTTCGATCCCCGGCAACGGCG
Asp_tRNA34  GTCGTTGTAGTATAGT-GGTGAGTATTCCCGCCTGTCACGCGGGTG--ACCCGGGTTCGATCCCCGGCAACGGCG
Asp_tRNA8_  GTCGTTGTAGTATAGT-GGTGAGTATTCCCGCCTGTCACGCGGGTG--ACCCGGGTTCGATCCCCGGCAACGGCG
Asp_tRNA9_  GTCGTTGTAGTATAGT-GGTGAGTATTCCCGCCTGTCACGCGGGTG--ACCCGGGTTCGATCCCCGGCAACGGCG
Asp_tRNA1_  GTCGTTGTAGTATAGT-GGTGAGTATTCCCGCCTGTCACGCGGGTG--ATCCGGGTTCGATCCCCGGCAACGGCG
Asp_tRNA18  GTCGTTGTAGTATAGT-GGTGAGTATTCCCGCCTGTCACGCCGGTG--TCCCGGGTTCGATCCCCGGCAACGGCG
```

```
               AA      DA   DL       DA  ACA   ACL   ACA  VR  TA   TL    TA   AA
Cys_tRNA2_  GGGCCTGTAGCTCAGAGGATTAGAGCACGTGGCTACAAACCACGGTGTCGGGGGTTCAAATCCCTCCTCGCCCA
Cys_tRNA50  GGGTCCATAGCTCAGTGG--TAGAGCATTTGACTGCAGATCAAGAGGTCTCCGGTTCGAACCCGGATGGGCCCT
Cys_tRNA73  GGGTCCATAGCTCAGTGG--TAGAGCATTTGACTGCAGATCAAGAGGTCTCCGGTTCGAACCCGGATGGGCCCT
Cys_tRNA47  GGGTCCATAGCTCAGTGG--TAGAGCATTTGACTGCAGATCAAGAGGTCACCGGTTCGAACCCGGTTGGGCCCT
Cys_tRNA56  GGGTCCATAGCTCAGTGG--TAGAGCAATTGACTGCAGATCAATAGGTCTCCGGTTCGAACCCGGATGGGCCCT
Cys_tRNA32  GGGTCCATAGCTCAGTGG--TAGAGCAATTGACTGCAGATCAATAGGTCTCCGGTTCGAACCCGGATGGGCCCT
Cys_tRNA1_  GGGTCCATAGCTCAGTGG--TAGAGCAATTGACTGCAGATCAATAGGTCTCCGGTTCGAACCCGGATGGGCCCT
Cys_tRNA99  GGGTCCATAGCTCAGTGG--TAGAGCAATTGACTGCAGATCAATAGGTCACCGGTTCGAACCCGGTTGGGCCCT
Cys_tRNA33  GGGTCCATAGCTCAGTGG--TAGAGCAATTGACTGCAGATCAATAGGTCACCGGTTCGAACCCGGTTGGGCCCT
Cys_tRNA5_  GGGTCCATAGCTCAGTGG--TAGAGCAATTGACTGCAGATCAATAGGTTACCGGTTCGAACCCGGTTGGGCCCT
Cys_tRNA29  GGAAGCATGGCCAAGCGG--TAAGGCAGGGGACTGCAAATC-CTTTATCCCCAGTTCAAATCTGGGTGCCGCCT
Cys_tRNA43  GGCAGCATGGCCAAGCGG--TAAGGCAGGGGACTGCAAATC-CTTTATCCCCAGTTCAAATCTGGGTACCGCCT
Cys_tRNA59  GGCGGCATGGCCAAGCGG--TAAGGCAGGGGACTGCAAATC-CTTTATCCCCAGTTCAAATCTGGGTGCCGCCT
Cys_tRNA12  GGCGGCATGGCCAAGCGG--TAAGGCAGGGGACTGCAAATC-CTTTATCCCCAGTTCAAATCTGGGTGCCGCCT
Cys_tRNA78  GGCGGCATGGCCAAGCGG--TAAGGCAGGGGACTGCAAATC-CTTTATCCCCAGTTCAAATCTGGGTGCCGCCT
Cys_tRNA86  GGCGGCATGGCCAAGCGG--TAAGGCAGGGGACTGCAAATC-CTTTATCCCCAGTTCAAATCTGGGTGCCGCCT
Cys_tRNA23  GTTCTGGTAGCTCAGCTGGTTAGAGCAAAGGACTGCAAATCCT----TCAGTGGTTCGATTCCACCTCAGAACG
Cys_tRNA5_  GTTCTGGTAGCTCAGCTGGTTAGAGCAAAGGACTGCAAATCCT----TCAGTGGTTCGATTCCACCTCAGAACG
```

```
              AA         DA    DL     DA   ACA   ACL   ACA  VR   TA    TL    TA    AA
Gln_tRNA32   GGCCCCATGGTCTAGCGGTTAGGACATTGGACTCTGAATCCAGTAACCCG-AGTTCAAATCTCGGTGGGACCT
Gln_tRNA9_   GGTCCCATGGTCTAGCGGTTAGGACATTGGACTCTGAATCCAGTAACCCG-AGTTCAAATCTCGGTGGGACCT
Gln_tRNA26   GGTCCCATGGTCTAGCGGTTAGGACATTGGACTCTGAATCCAGTAACCCG-AGTTCAAATCTCGGTGGGACCT
Gln_tRNA29   GGTCCCATGGTCTAGCGGTTAGGACATTGGACTCTGAATCCAGCAACCCG-AGTTCAAATCTCGGTGGGACCT
Gln_tRNA11   GGTTCCATGGTCTAGCGGTTAGGACATTGGACTCTGAATCCAGTAACCCG-AGTTCAAATCTCGGTGGAACCT
Gln_tRNA7_   GGTTCCATGGTCTAGCGGTTAGGACATTGGACTCTGAATCCAGTAACCCG-AGTTCAAATCTCGGTGGAACCT
Gln_tRNA27   GGTCCCATGGTCTAGTGGTCAGGACATTGGACTCTGAATCCAGTAACCCG-AGTTCAAATCTCGGTGGGACCT
Gln_tRNA29   GGTCCCATGGTCTAGTGGTCAGGACATTGGACTCTGAATCCAGTAACCCG-AGTTCAAATCTCGGTGGGACCT
Gln_tRNA38   GGTCCCATGGTCTAGCGGTTAGGACATTAGACTCTGAATCTAGTAACCCG-AGTTCAAATCTCGGTGGGACCT
Gln_tRNA25   GGTCCCATGGTCTAGCGGTTAGGACATTAGACTCTGAATCTAGTAACCCG-AGTTCAAATCTCGGTGGGACCT
Gln_tRNA68   GGTTCCATAGTGTAGTGGTTAGCACTCCAGACTTTGAATCTAGCAAACTG-GGTTCAAATCCCGGTGGGACCT
Gln_tRNA64   GGTCCCATAGTGTAGTGGTTAGCACTCCAGACTTTGAATCTGGCAACCTG-GGTTCAAATCCCTGTGGGACCT
Gln_tRNA67   GGTTCCATAGTGTAGTGGTTAGCACTCCAGACTTTGAATCTGGCAACCTG-GGTTCAAATCCCGGTGGGACCT
Gln_tRNA69   GGTTCCATAGTGTAGTGGTTAGCACTCCAGACTTTGAATCTGGCAACCTG-GGTTCAAATCCCGGTGGGACCT
Gln_tRNA65   GGTTCCATAGTGTAGTGGTTAGCACTCCAGACTTTGAATCTGGCAACCTG-GGTTCAAATCCCGGTGGAACCT
Gln_tRNA66   GGTTCCATAGTGTAGTGGTTAGCACTCCAGACTTTGAATCTGGCAACCTG-GGTTCAAATCCCGGTGGAACCT
Gln_tRNA70   GGTTCCATAGTGTAGTGGTTAGCACTCCAGACTTTGAATCTGGCAACCTG-GGTTCAAATCCCGGTGGAACCT
Gln_tRNA63   GGTTCCATAGTGTAGTGGTTAGCACTCCAGACTTTGAATCTGGCAACCTG-GGTTCGAATCCCGGTGGGACCT
Gln_tRNA17   GGTTCCATAGTGTAGTGGTTAGCACTCCAGACTTTGAATCTGGCGACCTG-GGTTCGAATCCCGGTGGGACCT
Gln_tRNA43   GGTTCCATAGTGTAGTGGTTAGCACTCCAGACTTTGAATCTGGCGACCTG-GGTTCGAATCCCGGTGGGACCT
Gln_tRNA66   GGTTCCATGGTGTAGTGGTTAGCACTCCAGACTTTGAATCTGGCGACCTG-GGTTCGAATCCCGGTGGGACCT
Gln_tRNA51   GGTTCCATGGTGTAGTGGTTAGCACTCCAGACTTTGAATCTGGCGACCTG-GGTTCGAATCCCGGTGGGACCT
Gln_tRNA52   GGTTCCATGGTGTAGTGGTTAGCACTCCAGACTTTGAATCTGGCGACCTG-GGTTCGAATCCCGGTGGGACCT
Gln_tRNA33   TGGGGCGTGGCCAAGTGGTAAGG-CAGCGGGTTTTGGTCCCATTACTCGGAGGTTCGAATCCTTCCATCCCAG
Gln_tRNA98   TGGGGCGTGGCCAAGTGGTAAGG-CAGCGGGTTTTGGTCCCGTTACTCGGAGGTTCGAATCCTTCCGTCCCAG
Gln_tRNA63   TGGGGCGTGGCCAAGTGGTAAGG-CAGCGGGTTTTGGTCCCGTTACTCGGAGGTTCGAATCCTTCCGTCCCAG
Gln_tRNA84   TGGGGCGTGGCCAAGTGGTAAGG-CAGCGGGTTTTGGTCCCGTTACTCGGAGGTTCGAATCCTTCCGTCCCAG
Gln_tRNA90   TGGGGCGTGGCCAAGTGGTAAGG-CAGCGGGTTTTGGTCCCGTTACTCGGAGGTTCGAATCCTTCCGTCCCAG
Gln_tRNA39   TGGGGCGTGGCCAAGTGGTAAGG-CAGCGGGTTTTGGTCCCGTTACTCGGAGGTTCGAATCCTTCCGTCCCAG
Gln_tRNA30   TGGAGTATAGCCAAGCGGTAAGG-CATCGGTTTTTGGTCCCGGCATGCAAAGGTTCGAATCCTTTTACTCCAG
Gln_tRNA28   TGGAGTATAGCCAAGTGGTAAGG-CATCGGTTTTTGGTACCGGCATGCAAAGGTTCGAATCCTTTTACTCCAG
```

```
                AA        DA        DL          DA      ACA    ACL    ACA   VR  TA    TL    TA    AA
Glu_tRNA11   TCCGTTGTAATCTACTTGTAGCTGGTTAGGATAATCGGCTCTCACCCGAGAGACCCGGGTTCGAGTCCCGGCAACGGAA
Glu_tRNA2_   TCCGTTGTAGTCTA------GCTGGTTAG-ATACTCGGCTCTCACCCGAGAGACCCGGGTTCGAGTCCCGGCAACGGAA
Glu_tRNA46   TCCGTTGTAGTCTA------GCTGGTTGGGATACTCGGCTCTCACCCGAGAGACCCGGGTTCGAGTCCCGGCAACGGAA
Glu_tRNA10   TCCGTTGTAGTCTA------GCTGGTTAGGATACTCGGCTCTCACCCGAGAGACCCGGGTTCGAGTCCCGGCAACGGAA
Glu_tRNA3_   TCCGTTGTAGTCTA------GCTGGTTAGGATACTCGGCTCTCACCCGAGAGACCCGGGTTCGAGTCCCGGCAACGGAA
Glu_tRNA70   TCCGTTGTAGTCTA------GCTGGTTAGGATACTCGGCTCTCACCCGAGAGACCCGGGTTCGAGTCCCGGCAACGGAA
Glu_tRNA9_   TCCGTTGTAGTCTA------GCTGGTTAGGATACTCGGCTCTCACCCGAGAGACCCGGGTTCGAGTCCCGGCAACGGAA
Glu_tRNA35   TCCGTTGTAGTCTA------GCTGGTTAGGATACTCGGCTCTCACCCGAGAGACCCGGGTTCGAGTCCCGGCAACGGAA
Glu_tRNA72   TCCGTTGTAGTCTA------GCTGGTTAGGATACTCGGCTCTCACCCGAGAGACCCGGGTTCGAGTCCCGGCAACGGAA
Glu_tRNA74   TCCGTTGTAGTCTA------GCTGGTTAGGATACTCGGCTCTCACCCGAGAGACCCGGGTTCGAGTCCCGGCAACGGAA
Glu_tRNA66   TCCGTTGTAGTCTA------GCTGGTTAGGATACTCGGCTCTCACCCGAGAGACCCGGGTTCGAGTCCCGGCAACGGAA
Glu_tRNA32   TCCGTTGTAGTCTA------GCTGGTTAGGATACTCGGCTCTCACCCGAGAGACCCGGGTTCGAGTCCCGGCAACGGAA
Glu_tRNA46   TCCGTTGTAGTCTA------GCTGGTTAGGATACTCGGCTCTCACCCGAGAGACCCGGGTTCGAGTCCCGGCAACGGAA
Glu_tRNA49   TCCGTTGTAGTCTA------GCTGGTTAGGATACTCGGCTCTCACCCGAGAGACCCGGGTTCGAGTCCCGGCAACGGAA
Glu_tRNA1_   TCCGTTGTAGTCTA------GCTGGTTAGGATACTCGGCTCTCACCCGAGAGACCCGGGTTCGAGTCCCGGCAACGGAA
Glu_tRNA35   TCCGTTGTAGTCTA------GCTGGTTAGGATACTCGGCTCTCACCCGAGAGACCCGGGTTCGAGTCCCGGCAACGGAA
Glu_tRNA30   TCCGTTGTAGTCTA------GCTGGTTAGGATACTCGGCTCTCACCCGAGAGACCCGGGTTCGAGTCCCGGCAACGGAA
Glu_tRNA44   TCCGTTGTAGTCTA------GCTGGTTAGGATACTCGGCTCTCACCCGAGAGACCCGGGTTCGAGTCCCGGCAACGGAA
Glu_tRNA13   TCCGTTGTAGTCTA------GCTGGTTAGGATACTCGGCTCTCACCCGAGAGACCCGGGTTCGAGTCCCGGCATCGGAA
Glu_tRNA37   TCCGTTGTGGTCTA------GCTGGTTAGGATACTCGGCTCTCACCCGAGAGACCCGGGTTCGAGTCCCGGCAACGGAA
Glu_tRNA71   TCCGTTGTAGTCTA------GCTGGTTAGGATACTCGGCTCTCACCCGAGAGACCCGGGTTCGAGTCCCGGCAACGGAA
Glu_tRNA39   TCCGTTGTAGTCTA------GCTGGTTAGGATACTCGGCTCTCACCCGAGAGACCCGGGTTCGAGTCCCGGCAACGGAA
Glu_tRNA21   TCCGTTGTAGTCTA------GCTGGTTAGGATACTCGGCTCTCACCCGAGAGACCCGGGTTCGAGTCCCGGCAACGGAA
Glu_tRNA62   TTCGTTGTAGTCTA------GCTGGTTAGGATACTCGGCTCTCACCCGAGAGACCCGGGTTCGAGTCCCGGCAACGGAA
Glu_tRNA39   TCCGTTGTAGTCTA------GCTGGTTAGGATACTTGGCTCTCACCCAAGAGACCCGGGTTCGAGTCCCGGCAACGGAA
Glu_tRNA2_   TCCATTGTCGTCTA-----GTCCGGTTAGGATACCTGGCTTTCACCCAGGCGACCCGGGTTCAAATCCCTGCAATGGAA
Glu_tRNA12   TCCATTGTCGTCTA-----GTCCGGTCAGGATACCTGGCTTTCACCCAGGCGACCCGGGTTCAAATCCCGGCAATGGAA
Glu_tRNA5_   TCCATTGTCGTCTA-----GTCCGGTTAGGATACCTGGCTTTCACCCAGGCGACCCGGGTTCAAATCCCGGCAATGGAA
Glu_tRNA73   TCCATTGTCGTCTA-----GTCCGGTTAGGATACCTGGCTTTCACCCAGGCGACCCGGGTTCAAATCCCGGCAATGGAA
Glu_tRNA56   TCCATTGTCGTCTA-----GTCCGGTTAGGATACCTGGCTTTCACCCAGGCGACCCGGGTTCAAATCCCGGCAATGGAA
Glu_tRNA5_   TCCATTGTCGTCTA-----GTCCGGTTAGGATACCTGGCTTTCACCCAGGCGACCCGGGTTCAAATCCCGGCAATGGAA
Glu_tRNA4_   TCCATTGTCGTCTA-----GTCCGGTTAGGATACCTGGCTTTCACCCAGGCGACCCGGGTTCAAATCCCGGCAATGGAA
Glu_tRNA37   TCCATTGTCGTCTA-----GTCCGGTTAGGATATCTGGCTTTCACCCAGACGACCCGGGTTCAAATCCCGGCAATGGAA
Glu_tRNA43   TCCATTGTCGTCTA-----GTCCGGTTAGGATATCTGGCTTTCACCCAGACGACCCGGGTTCAAATCCCGGCAATGGAA
Glu_tRNA52   TCCATTGTCGTCTA-----GTCCGGTTAGGATATCTGGCTTTCACCCAGACGACCCGGGTTCAAATCCCGGCAATGGAA
Glu_tRNA6_   TCCGTTGTCGTCTA-----G--CGGTTAGGATATCTGGCTTTCACCCAGGAGACCCGGGTTCGATTCCCGGCAACGGAA
Glu_tRNA78   GCCCCTATCGTCTA------GTGGTTCAGGACATCTCTCTTTCAAGGAGGCAGCGGGGATTCGACTTCCCCTGGGGGTA
Glu_tRNA56   GCCCCTATCGTCTA------GTGGTTCAGGACATCTCTCTTTCAAGGAGGCAGCGGGGATTCGACTTCCCCTGGGGGTA
Glu_tRNA13   GCCCCTATCGTCTA------GTGGTTCAGGACATCTCTCTTTCAAGGAGGCAGCGGGGATTCGACTTCCCCTGGGGGTA
Glu_tRNA24   GCCCCTATCGTCTA------GTGGTTCAGGACATCTCTCTTTCAAGGAGGCAGCGGGGATTCGACTTCCCCTGGGGGTA
Glu_tRNA7_   GCCCCTATCGTCTA------GTGGTTCAGGACATCTCTCTTTCAAGGAGGCAGCGGGGATTCGACTTCCCCTGGGGGTA
```

```
             AA     DA   DL    DA    ACA   ACL   ACA VR  TA   TL   TA   AA
Gly_tRNA5_   GCGCATCTGGTGTAGTGGTAT--CATAGTACCCTCCCACGGTACTGACC-GGGTTCGATTCCCCGGATGCGCA
Gly_tRNA85   GCGCATCTGGTGTAGTGGTAT--CATAGTACCCTCCCACGGTACTGACCAAGGTTCGATTCCCTGGATGCGCA
Gly_tRNA8_   GCGCATCTGGTGTAGTGGTAT--CATAGTACCCTCCCACGGTACTGACCAGGGTTCGATTCCCTGGATGCGCA
Gly_tRNA42   GCGCATCTGGTGTAGTGGTAT--CATAGTACCCTCCCACGGTACTGACCGGGGTTCGATTCCCCGGATGAGCA
Gly_tRNA86   GCGCATCTGGTGTAGTGGTAT--CATAGTACCCTCCCACGGTACTGACCGGGGTTCGATTCCCCGGATGCGCA
Gly_tRNA30   GCGCATCTGGTGTAGTGGTAT--CATAGTACCCTCCCACGGTACTGACCGGGGTTCGATTCCCCGGATGCGCA
Gly_tRNA10   GCGCATCTGGTGTAGTGGTAT--CATAGTACCCTCCCACGGTACTGACCGGGGTTCGATTCCCCGGATGCGCA
Gly_tRNA30   GCGCATCTGGTGTAGTGGTAT--CATAGTACCCTCCCACGGTACTGACCGGGGTTCGATTCCCCGGATGCGCA
Gly_tRNA29   GCGCATCTGGTGTAGTGGTAT--CATAGTACCCTCCCACGGTACTGACCGGGGTTCGATTCCCCGGATGCGCA
Gly_tRNA8_   GCGCCAGTGGTCTAGTGGTAG--AATAGTACCCTGCCACGGTACAGACCCGGGTTCGATTCCCTGCTGGTGCA
Gly_tRNA36   GCACCAGTGGTCTAGTGGTAG--AATAGTACCCTGCCACGGTACGGACCCGGGTTCGATTCCCGGCTGGTGCA
Gly_tRNA8_   GCACCAGTGGTCTAGTGGTAG--AATAGTACCCTGCCACGGTACAGACCCGGGTTCGATTCCCGGCTGGTGCA
Gly_tRNA19   GCACCAGTGGTCTAGTGGTAG--AATAGTACCCTGCCACGGTACAGACCCGGGTTCGATTCCCGGCTGGTGCA
Gly_tRNA30   GCACCAGTGGTCTAGTGGTAG--AATAGTACCCTGCCACGGTACAGACCCGGGTTCGATTCCCGGCTGGTGCA
Gly_tRNA52   GCACCAGTGGTCTAGTGGTAG--AATAGTACCCTGCCACGGTACAGACCCGGGTTCGATTCCCGGCTGGTGCA
Gly_tRNA44   GCACCAGTGGTCTAGTGGTAG--AATAGTACCCTGCCACGGTACAGACCCGGGTTCGATTCCCGGCTGGTGCA
Gly_tRNA7_   GCACCAGTGGTCTAGTGGTAG--AATAGTACCCTGCCACGGTACAGACCCGGGTTCGATTCCCGGCTGGTGCA
Gly_tRNA4_   GCACCAGTGGTCTAGTGGTAG--AATAGTACCCTGCCACGGTACAGACCCGGGTTCGATTCCCGGCTGGTGCA
Gly_tRNA24   GCACCAGTGGTCTAGTGGTAG--AATAGTACCCTGCCACGGTACAGACCCGGGTTCGATTCCCGGCTGGTGCA
Gly_tRNA55   GCACCAGTGGTCTAGTGGTAG--AATAGTACCCTGCCACGGTACAGACCCGGGTTCGATTCCCGGCTGGTGCA
Gly_tRNA29   GCACCAGTGGTCTAGTGGTAG--AATAGTACCCTGCCACGGTACAGACCCGGGTTCGATTCCCGGCTGGTGCA
Gly_tRNA14   GCACCAGTGGTCTAGTGGTAG--AATAGTACCCTGCCACGGTACAGACCCGGGTTCGATTCCCGGCTGGTGCA
Gly_tRNA63   GCACCAGTGGTCTAGTGGTAG--AATAGTACCCTGCCACGGTACAGACCCGGGTTCGATTCCCGGCTGGTGCA
Gly_tRNA8_   GCACCAGTGGTCTAGTGGTAG--AATAGTACCCTGCCACGGTACAGACCCGGGTTCGATTCCCGGCTGGTGCA
Gly_tRNA77   GCACCAGTGGTCTAGTGGTAG--AATAGTACCCTGCCACGGTACAGACCCGGGTTCGATTCCCGGCTGGTGCA
Gly_tRNA34   GCACCAGTGGTCTAGTGGTAG--AATAGTACCCTGCCACGGTACAGACCCGGGTTCGATTCCCGGCTGGTGCA
Gly_tRNA24   GCACCAGTGGTCTAGTGGTAG--AATAGTACCCTGCCACGGTACAGACCCGGGTTCGATTCCCGGCTGGTGCA
Gly_tRNA1_   GCACCAGTGGTCTAGTGGTAG--AATAGTACCCTGCCACGGTACAGACCCGGGTTCGATTCCCGGCTGGTGCA
Gly_tRNA71   GCACCAGTGGTCTAGTGGTAG--AATAGTACCCTGCCACGGTACAGACCCGGGTTCGATTCCCGGCTGGTGCA
Gly_tRNA28   GCACCAGTGGTCTAGTGGTAG--AATAGTACCCTGCCACGGTACAGACCCGGGTTCGATTCCCGGCTGGTGCA
Gly_tRNA42   GCACCAGTGGTCTAGTGGTAG--AATAGTACCCTGCCACGGTACAGACCCGGGTTCGATTCCCGGCTGGTGCA
Gly_tRNA54   GCGAGCGTAGTTCAATGGTAA--AACATCACCTTGCCAAGGAGAAGATACGGGTTCGATTCCCGCCGCTCGCC
Gly_tRNA37   GCGAGCGTAGTTCAATGGTAA--AACATCTCCTTGCCAAGGAGAAGATACGGGTTCGATTCCCGCCGCTCGCC
Gly_tRNA5_   GCGAGCGTAGTTCAATGGTAA--AACATCTCCTTGCCAAGGAGAAGATACGGGTTCGATTCCCGCCGCTCGCC
Gly_tRNA11   GCGAGCGTAGTTCAATGGTAA--AACATCTCCTTGCCAAGGAGAAGATACGGGTTCGATTCCCGCCGCTCGCC
Gly_tRNA22   GCGAGCGTAGTTCAATGGTAA--AACATCTCCTTGCCAAGGAGAAGATACGGGTTCGATTCCCGCCGCTCGCC
Gly_tRNA57   GCGAGCGTAGTTCAATGGTAA--AACATCTCCTTGCCAAGGAGAAGATATGGGTTCGATTCCCGCCGCTCGCC
Gly_tRNA23   GCGTTTGTAGTCCAACGGTTAGCATAATTGCCTT-CCAAGCAATAGACCCGGGTTCGACTCCCGGCAAACGCA
Gly_tRNA45   GCGTTTGTAGTCCAACGGTTAGGATAATTGCCTT-CCAAGCAATAGACCCGGGTTCGACTCCCGGCAGACGCA
Gly_tRNA32   GCGTCTGTAGTCCAACGGTTAGGATAATTGCCTT-CCAAGCAATAGACCCGGGTTCGACTCCCGGCAGACGCA
Gly_tRNA40   GCGTTTGTAGTCCAACGGTTAGGATAATTGCCTT-CCAAGCAATAGACCCGGGTTCGACTCCCGGCAAACGCA
Gly_tRNA67   GCGTTTGTAGTCCAACGGTTAGGATAATTGCCTT-CCAAGCAATAGACCCGGGTTCGACTCCCGGCAAACGCA
Gly_tRNA36   GCGTTTGTAGTCCAACGGTTAGGATAATTGCCTT-CCAAGCAATAGACCCGGGTTCGACTCCCGGCAAACGCA
Gly_tRNA6_   GCGTTTGTAGTCCAACGGTTAGGATAATTGCCTT-CCAAGCAATAGACCCGGGTTCGACTCCCGGCAAACGCA
Gly_tRNA2_   GCGTTTGTAGTCCAACGGTTAGGATAATTGCCTT-CCAAGCAATAGACCCGGGTTCGACTCCCGGCAAACGCA
Gly_tRNA3_   GCGTTTGTAGTCCAACGGTTAGGATAATTGCCTT-CCAAGCAATAGACCCGGGTTCGACTCCCGGCAAACGCA
Gly_tRNA38   GCGTTCGTAGTCCAACGGTTAGGATAATTGCCTT-CCAAGCAATAGACCCGGGTTCGACTCCCGGCGAACGCA
```

```
             AA      DA   DL     DA   ACA   ACL    ACA  VR  TA    TL    TA   AA
His_tRNA7_   ACGGATGTAGCCAAGTTGATCAAGGCAGTGGATTGTGAATCCACCATGCGCGGGTTCAATTCCCGTCGTTCGCC
His_tRNA41   GCGGATGTAGCCAAGTGGATCAAGGCAGTGGATTGTGAATCCACCATGCGCGGGTTCAATTCCCATTGTTCGCC
His_tRNA13   GCGGATGTAGCCAAGTGGATCAAGGCAGTGGATTGTGAATCCACCATGCGCGGGTTCAATTCCCGTTGTTCGCC
His_tRNA64   GCGGATGTAGCCAACTGGATCAAGGCAGTGGATTGTGAATCCACCATGCGCGGGTTCAATTCCCGTCGTTCGCC
His_tRNA27   GCGGATGTAGCCAAGTGGATCAAGGCAGTGGATTGTGAATCCACCATGCGCGGGTTCAATTCCCGTCGTTCGCC
His_tRNA19   GCGGATGTAGCCAAGTGGATCAAGGCAGTGGATTGTGAATCCACCATGCGCGGGTTCAATTCCCGTCGTTCGCC
His_tRNA7_   GCGGATGTAGCCAAGTGGATCAAGGCAGTGGATTGTGAATCCACCATGCGCGGGTTCAATTCCCGTCGTTCGCC
His_tRNA85   GCGGATGTAGCCAAGTGGATCAAGGCAGTGGATTGTGAATCCACCATGCGCGGGTTCAATTCCCGTCGTTCGCC
His_tRNA91   GCGGATGTAGCCAAGTGGATCAAGGCAGTGGATTGTGAATCCACCATGCGCGGGTTCAATTCCCGTCGTTCGCC
His_tRNA40   GCGGATGTAGCCAAGTGGATCAAGGCAGTGGATTGTGAATCCACCATGCGCGGGTTCAATTCCCGTCGTTCGCC
His_tRNA27   GCGGATGTAGCCAAGTGGATCAAGGCAGTGGATTGTGAATCCACCATGCGCGGGTTCAATTCCCGTCGTTCGCC
His_tRNA46   GCGGATGTAGCCAAGTGGATCAAGGCAGTGGATTGTGAATCCACCATGCGCGGGTTCAATTCCCGTCGTTCGCC
His_tRNA28   GCGGATGTAGCCAAGTGGATCAAGGCAGTGGATTGTGAATCCACCATGCGCGGGTTCAATTCCCGTCGTTCGCC
His_tRNA32   GCGGATGTAGCCAAGTGGATCAAGGCAGTGGATTGTGAATCCACCATGCGCGGGTTCAATTCCCGTCGTTCGCC
His_tRNA16   GCGGATGTAGCCAAGTGGATCAAGGCAGTGGATTGTGAATCCACCATGCGCGGGTTCAATTCCCGTCGTTCGCC
His_tRNA52   TCGGCAGTAGTTTAGTGGTGAGAATTCCACG-TTGTGG-TCGTGGAGACCTGGGCTCGAATCCCAGCAGACGAG
His_tRNA13   GTGGCTGTAGTTTAGTGGTGAGAATTCCACG-TTGTGG-CCGTGGAGACCTGGGCTCGAATCCCAGCAGCCACA
His_tRNA25   GTGGCTGTAGTTTAGTGGTGAGAATTCCACG-TTGTGG-CCGTGGAGACCTGGGCTCGAATCCCAGCAGCCACA
His_tRNA79   GTGGCTGTAGTTTAGTGGTGAGAATTCCACG-TTGTGG-CCGTGGAAGCCTGGGCTCGAATCCCAGCAGCCACA
His_tRNA75   GTGGCTGTAGTTTAGTGGTGAGAATTCCACG-TTGTGG-CCGTGGAGACCTGGGCTCGAATCCCAGCAGCCACA
His_tRNA15   GTGGCTGTAGTTTAGTGGTGAGAATTCCACG-TTGTGG-CCGTGGAGACCTGGGCTCGAATCCCAGCAGCCACA
His_tRNA18   GTGGCTGTAGTTTAGTGGTGAGAATTCCACG-TTGTGG-CCGTGGAGACCTGGGCTCGAATCCCAGCAGCCACA
His_tRNA23   GTGGCTGTAGTTTAGTGGTGAGAATTCCACG-TTGTGG-CCGTGGAGACCTGGGCTCGAATCCCAGCAGCCACA
His_tRNA27   GTGGCTGTAGTTTAGTGGTGAGAATTCCACG-TTGTGG-CCGTGGAGACCTGGGCTCGAATCCCAGCAGCCACA
His_tRNA7_   GTGGCTGTAGTTTAGTGGTGAGAATTCCACG-TTGTGG-CCGTGGAGACCTGGGCTCGAATCCCAGCAGCCACA
His_tRNA53   GTGGCTGTAGTTTAGTGGTGAGAATTCTACG-TTGTGG-CCGTAGAGACCTGGGCTCGAATCCCAGCAGCCACA
```

```
            AA      DA   DL   DA   ACA   ACL   ACA   VR   TA   TL   TA   AA
Ile_tRNA42  GGCCTATTAGCTCAGCTGGTTAGAGCGTCGTACTAATAACGCGAAGGTCGCAGGTTCGAGACCTTCATGGGCCA
Ile_tRNA27  GGCCTATTAGCTCAGCTGGTTAGAGCGTCGTGCTAATAACGCGAAGGTCGCAGGTTCGAGACCTGCATGGGCCA
Ile_tRNA39  GGCCTATTAGCTCAGCTGGTTAGAGCGTCGTGCTAATAACGCGAAGGTCGCAGGTTCGAGACCTGCATGGGCCA
Ile_tRNA45  GGCCTATTAGCTCAGCTGGTTAGAGCGTCGTGCTAATAACGCGAAGGTCGCAGGTTCGAGACCTGCATGGGCCA
Ile_tRNA54  GGCCTATTAGCTCAGCTGGTTAGAGCGTCGTGCTAATAACGCGAAGGTCGCAGGTTCGAGACCTGCATGGGCCA
Ile_tRNA15  GGCCTATTAGCTCAGCTGGTTAGAGCGTCGTGCTAATAACGCGAAGGTCGCAGGTTCGAGACCTGCATGGGCCA
Ile_tRNA41  GGCCTATTAGCTCAGCTGGTTAGAGCGTCGTGCTAATAACGCGAAGGTCGCAGGTTCGAGACCTGCATGGGCCA
Ile_tRNA22  GGCCTATTAGCTCAGCTGGTTAGAGCGTCGTGCTAATAACGCGAAGGTCGCAGGTTCGAGACCTGCATGGGCCA
Ile_tRNA37  GGCCTATTAGCTCAGCTGGTTAGAGCGTCGTGCTAATAACGCGAAGGTCGCAGGTTCGAGACCTGCATGGGCCA
Ile_tRNA65  GGCCTATTAGCTCAGCTGGTTAGAGCGTCGTGCTAATAACGCGAAGGTCGCAGGTTCGAGACCTGCATGGGCCA
Ile_tRNA64  GGCCTATTAGCTCAGCTGGTTAGAGCGTCGTGCTAATAACGCGAAGGTCGCAGGTTCGAGACCTGCATGGGCCA
Ile_tRNA39  GGCCTATTAGCTCAGCTGGTTAGAGCGTCGTGCTAATAACGCGAAGGTCGCAGGTTCGAGACCTGCATGGGCCA
Ile_tRNA62  GGCCTATTAGCTCAGCTGGTTAGAGCGTCGTGCTAATAACGCGAAGGTCGCAGGTTCGAGACCTGCATGGGCCA
Ile_tRNA21  GGCCTATTAGCTCAGCTGGTTAGAGCGTCGTGCTAATAACGCGAAGGTCGCAGGTTCGAGACCTGCATGGGCCA
Ile_tRNA1_  GGCCTATTAGCTCAGCTGGTTAGAGCGTCGTGCTAATAACGCGAAGGTCGCAGGTTCGAGACCTGCATGGGCCA
Ile_tRNA17  GGCCTATTAGCTCAGCTGGTTAGAGCGTCGTGCTAATAACGCGAAGGTCGCAGGTTCGAGACCTGCATGGGCCA
Ile_tRNA90  GGCCTATTAGCTCAGCTGGTTAGAGCGTCGTGCTAATAACGCGAAGGTCGCAGGTTCGAGACCTGCATGGGCCA
Ile_tRNA28  GGCCTATTAGCTCAGCTGGTTAGAGCGTCGTGCTAATAACGCGAAGGTCGCAGGTTTGAGACCTGCATGGGCCA
Ile_tRNA13  GGTCCCGTAGCTCAGTTGGTTAGAGCGTTGATCTTATGAGCCGAAGGTCGCGGGTTCGAGCCCCGCCGGGACCA
Ile_tRNA96  GGTCCCGTAGCTCAGTTGGTTAGAGCGTTGGTCTTATGAGCCGAAGGTCGCGGGTTCGAGCCCCGCCGGGACCA
Ile_tRNA44  GGTCCCGTAGCTCAGTTGGTTAGAGCGTTGGTCTTATGAGCCGAAGGTCGCGGGTTCGAGCCCCGCCGGGACCA
Ile_tRNA10  GGTCCCGTAGCTCAGTTGGTTAGAGCGTTGGTCTTATGAGCCGAAGGTCGCGGGTTCGAGCCCCGCCGGGACCA
Ile_tRNA63  GGTCCCGTAGCTCAGTTGGTTAGAGCGTTGGTCTTATGAGCCGAAGGTCGCGGGTTCGAGCCCCGCCGGGACCA
```

```
Leu_tRNA7_  GTTGAGATGGCCGAGTTGGTCTAAGGCGCCAGATTAAGGTTCTGGTCC----GAAAGGGCGTGGATTCAAATCCCACTCTCAACA
Leu_tRNA47  GTTGAGATGGCCGAGTTGGTCTAAGGCGCCAGATTAAGGTTCTGGTCC----GAAAGGGCGTGGGTTCAAATCCCACTCTCAACA
Leu_tRNA60  GTTGAGATGGCCGAGTTGGTCTAAGGCGCCAGATTAAGGTTCTGGTCC----GAAAGGGCGTGGGTTCAAATCCCACTCTCAACA
Leu_tRNA72  GTTGAGATGGCCGAGTTGGTCTAAGGCGCCAGATTAAGGTTCTGGTCC----GAAAGGGCGTGGGTTCAAATCCCACTCTCAACA
Leu_tRNA95  GTTGAGATGGCCGAGTTGGTCTAAGGCGCCAGATTAAGGTTCTGGTCC----GAAAGGGCGTGGGTTCAAATCCCACTCTCAACA
Leu_tRNA51  GTTGAGATGGCCGAGTTGGTCTAAGGCGCCAGATTAAGGTTCTGGTCC----GAAAGGGCGTGGGTTCAAATCCCACTCTCAACA
Leu_tRNA69  GTTGAGATGGCCGAGTTGGTCTAAGGCGCCAGATTAAGGTTCTGGTCC----GAAAGGGCGTGGGTTCAAATCCCACTCTCAACA
Leu_tRNA33  GTTGAGATGGCCGAGTTGGTCTAAGGCGCCAGATTAAGGTTCTGGTCC----GAAAGGGCGTGGGTTCAAATCCCACTCTCAACA
Leu_tRNA20  GTTGAGATGGCCGAGTTGGTCTAAGGCGCCAGATTAAGGTTCTGGTCC----GAAAGGGCGTGGGTTCAAATCCCACTCTCAACA
Leu_tRNA33  GTTGAGATGGCCGAGTTGGTCTAAGGCGCCAGATTAAGGTTCTGGTCC----GAAAGGGCGTGGGTTCAAATCCCACTCTCAACA
Leu_tRNA8_  GTTGAGATGGCCGAGTTGGTCTAAGGCGCCAGATTAAGGTTCTGGTCC----GAAAGGGCGTGGGTTCAAATCCCACTCTCAACA
Leu tRNA17  GTTGAGATGGCCGAGTTGGTCTAAGGCGCCAGATTAAGGTTCTGGTCC----GAAAGGGCGTGGGTTCAAATCCCACTCTCAACA
Leu_tRNA37  GTTGAGATGGCCGAGTTGGTCTAAGGCGCCAGATTAAGGTTCTGGTCC----GAAAGGGCGTGGGTTCAAATCCCACTCTCAACA
Leu_tRNA37  GTTGAGATGGCCGAGTTGGTCTAAGGCGCCAGATTAAGGTTCTGGTCC----GAAAGGGCGTGGGTTCAAATCCCACTCTTAACA
Leu_tRNA1_  GTTGAGATGGCCGAGTTGGTCCAAGGCGCCAGATTAAGGTTCTGGTCC----GAAAGGGCGTGGGTTCAAATCCCACTCTTAACA
Leu_tRNA10  GTCAAGATGGCCGAGTTGGTCTAAGGCGCCAGTTTCAGGTACTGGTCC----GAAAGGGCATGGGTTCGAATCCCATTCTTGACA
Leu_tRNA45  GTCAAGATGGCCGAGTTGGTCTAAGGCGCCAGTTTCAGGTACTGGTCC----GAAAGGGCATGGGTTCGAATCCCATTCTTGACA
Leu_tRNA16  GTCAAGATGGCCGAGTTGGTCTAAGGCGCCAGTTTCAGGTACTGGTCC----GAAAGGGCATGGGTTCGAATCCCATTCTTGACA
Leu_tRNA42  GTCAAGATGGCCGAGTTGGTCTAAGGCGCCAGTTTCAGGTACTGGTCC----GAAAGGGCATGGGTTCGAATCCCATTCTTGACA
Leu_tRNA13  GTCAAGATGGCCGAGTTGGTCTAAGGCGCCAGTTTCAGGTACTGGTCC----GAAAGGGCATGGGTTCGAATCCCATTCTTGACA
Leu_tRNA31  GTCAAGATGGCCGAGTTGGTCTAAGGCGCCAGTTTCAGGTACTGGTCC----GAAAGGGCATGGGTTCGAATCCCATTCTTGACA
Leu_tRNA11  GTCAAGATGGCCGAGTTGGTCTAAGGCGCCAGTTTCAGGTACTGGTCC----GAAAGGGCATGGGTTCGAATCCCATTCTTGACA
Leu_tRNA10  GTCAAGATGGCCGAGTTGGTCTAAGGCGCCAGTTTCAGGTACTGGTCC----GAAAGGGCATGGGTTCGAATCCCATTCTTGACA
Leu_tRNA4_  GTCAGGATGCCCGAGT-GGTCTAAGGCGCCAGACTCAAGTTCTGGTCCTCTAACGAGGGCGTGGGTTCAAATCCCACTTCTGACA
Leu_tRNA45  GTCAGGATGGCCGAGT-GGTCTAAGGCGCCAGACTCAAGTTCTGGTCCTCTAACGAGGGCGTGGGTTCAAATCCCACTTCTGACA
Leu_tRNA3_  GTCAGGATGGCCGAGT-GGTCTAAGGCGCCAGACTCAAGTTCTGGTCCTCTAACGAGGGCGTGGGTTCAAATCCCACTTCTGACA
Leu_tRNA33  GTCAGGATGGCCGAGT-GGTCTAAGGCGCCAGACTCAAGTTCTGGTCCTCTAACGAGGGCGTGGGTTCAAATCCCACTTCTGACA
Leu_tRNA2_  GTCAGGATGGCCGAGT-GGTCTAAGGCGCCAGACTCAAGTTCTGGTCCTCTAACGAGGGCGTGGGTTCAAATCCCACTTCTGACA
Leu_tRNA36  GTCAGGATGGCCGAGT-GGTCTAAGGCGCCAGACTCAAGTTCTGGTCCTCTAACGAGGGCGTGGGTTCAAATCCCACTTCTGACA
Leu_tRNA21  GTCAGGATGGCCGAGT-GGTCTAAGGCGCCAGACTCAAGTTCTGGTCCTCTAACGAGGGCGTGGGTTCAAATCCCACTTCTGACA
Leu_tRNA10  GTCAGGATGGCCGAGT-GGTCTAAGGCGCCAGACTCAAGTTCTGGTCCTCTAACGAGGGCGTGGGTTCAAATCCCACTTCTGACA
Leu tRNA49  GACGGTTTGGCCGAGT-GGTCTAAGGCGCCAGATTTAGGCTCTGGTCC----GAATGGGCGTGGGTTCAAATCCCACAGCTGTCA
Leu_tRNA67  GACAGTTTGGCCGAGT-GGTCTAAGGCGCCAGATTTAGGCTCTGGTCC----GAAAGGGCGTGGGTTCAAATCCCACAGCTGTCA
Leu_tRNA12  GACAGTTTGGCCGAGT-GGTCTAAGGCGCCAGATTTAGGCTCTGGTCC----GAAAGGGCGTGGGTTCAAATCCCACAGCTGTCA
Leu_tRNA2_  GACAGTTTGGCCGAGT-GGTCTAAGGCGCCAGATTTAGGCTCTGGTCC----GAAAGGGCGTGGGTTCAAATCCCACAGCTGTCA
Leu_tRNA72  GACAGTTTGGCCGAGT-GGTCTAAGGCGCCAGATTTAGGCTCTGGTCC----GTAAGGGCGTGGGTTCAAATCCCACAGCTGTCA
Leu_tRNA49   ACAGTTTGGCCGAGT-GGTCTAAGGCGCCAGATTTAGGCTCTGGTCC----GAAAGGGCGTGGGTTCAAATCCCACAGCTGTCA
Leu_tRNA50  GCTGGTTTGCCCGAGT-GGT-TAAGGGGGAAGACTTAAGATCTTGTGCACAGAAGTGCGCGTGGGTTCGAATCCCACAGCCAGCA
Leu_tRNA50  GCTGGTTTGCCCGAGT-GGT-TAAGGGGGAAGACTTAAGATCTTGTGCACTGAAGTGCGCGTGGGTTCGAATCCCACAGCCAGCA
Leu_tRNA67  GCTGGTTTGCCCGAGT-GGT-TAAGGGGGAAGACTTAAGATCTTGTGCACTGAAGTGCGCGTGGGTTCGAACCCCACAGCCAGCA
Leu_tRNA24  GCTGGTTTGCCCGAGT-GGT-TAAGGGGGAAGACTTAAGATCTTGTGCACATGAGTGCGCGTGGGTTCGAACCCCACAGCCAGCA
Leu_tRNA34  GCCTTGATGGTAAAAT-AGTAGACA-CGCGAGACTCAAAATCTCGTGCTA--AAGAGCGCGGAGGTTCGAGTCCTCTTCAAGGCA
Leu_tRNA38  GCATTGATGGTGAAAT-GGTAGACA-CGCGAGACTCAAAATCTCGTGCTA--AAGAGCGTGGAGGTTCGAGTCCTCTTCAAGGCA
Leu_tRNA3_  GCCTTGATGGTGAAAT-GGTAGACA-CGCGAGACTCAAAATCTCGTGCTA--AAGAGCGTGGAGGTTCGAGTCCTCTTCAAGGCA
Leu_tRNA52  GCCTTGATGGTGAAAT-GGTAGACA-CGCGAGACTCAAAATCTCGTGCTA--AAGAGCGTGGAGGTTCGAGTCCTCTTCAAGGCA
Leu_tRNA59  GCCTTGATGGTGAAAT-GGTAGACA-CGCGAGACTCAAAATCTCGTGCTA--AAGAGCGTGGAGGTTCGAGTCCTCTTCAAGGCA
Leu_tRNA8_  GCCTTGATGGTGAAAT-GGTAGACA-CGCGAGACTCAAAATCTCGTGCTA--AAGAGCGTGGAGGTTCGAGTCCTCTTCAAGGCA
Leu_tRNA81  GCCTTGATGGTGAAAT-GGTAGACA-CGCGAGACTCAAAATCTCGTGCTA--AAGAGCGTGGAGGTTCGAGTCCTCTTCAAGGCA
Leu_tRNA93  GCCTTGATGGTGAAAT-GGTAGACA-CGCGAGACTCAAAATCTCGTGCTA--AAGAGCGTGGAGGTTCGAGTCCTCTTCAAGGCA
Leu_tRNA8_  GCCTTGATGGTGAAAT-GGTAGACA-CGCGAGACTCAAAATCTCGTGCTA--AAGAGCGTGGAGGTTCGAGTCCTCTTCAAGGCA
Leu_tRNA13  GCCTTGATGGTGAAAT-GGTAGACA-CGCGAGACTCAAAATCTCGTGCTA--AAGAGCGTGGAGGTTCGAGTCCTCTTCAAGGCA
Leu tRNA13  GCCTTGATGGTGAAAT-GGTAGACA-CGCGAGACTCAAAATCTCGTGCTA--AAGAGCGTGGAGGTTCGAGTCCTCTTCAAGGCA
Leu_tRNA21  GCCTTGATGGTAAAAT-GGTAGACA-CGCGAGACTCAGAATCTCGTGCTA--ATAAGCGTGGAGGTTCGAGTCCTCTTCAAGGTA
Leu_tRNA38  GCCTTGATGGTAAAAT-GGTAGACA-CGCGAGACTCAGAATCTCGTGCTA--ATAAGCGTGGAGGTTCGAGTCCTCTTCAAGGTA
Leu_tRNA11  GCCGCCATGGTGAAATTGGTAGACA-CGCTGCTCTTAGGAAGCAGTGCTC--A--AGCATCTCGGTTCGAGTCCGAGTGGCGGCA
Leu_tRNA50  GCCGCCATGGTGAAATTGGTAGACA-CGCTGCTCTTAGGAAGCAGTGCTC--A--AGCATCTCGGTTCGAGTCCGAGTGGCGGCA
Leu tRNA14  GCCGCCATGGTGAAATTGGTAGACA-CGCTGCTCTTAGGAAGCAGTGCTC--A--AGCATCTCGGTTCGAGTCCGAGTGGCGGCA
```

```
                AA      DA     DL           DA    ACA    ACL      ACA   VR  TA    TL     TA     AA
Lys_tRNA59    GCCCGTCTAGCCCAGTCG-----GCAGAGCGCAAGGCTCTTAACCT---GGTCGTGGGTTTGAGCCCCACGGTGGGCG
Lys_tRNA26    GCCCGTCTAGCTCAGTCG-----GTAGAGCGCAAGGCTCTTAACCT---GGTCGTGGGTTCGAGCCCCACGGTGGGCG
Lys_tRNA35    GCCCGTCTAGCTCAGTCG-----GTAGAGCGCAAGGCTCTTAACCTTGTGGTCGTGGGTTCAAGCCCCACGGTGGGCG
Lys_tRNA32    GCCCGTCTAGCTCAGTCG-----GTAGAGCGCAAGGCTCTTAACCTTGTGGTCGTGGGTTCAAGCCCCACGGTGGGCG
Lys_tRNA25    GCCCGTCTAGCTCAGTCG-----GTAGAGCGCAAGGCTCTTAACCTTGTGGTCGTGGGTTCGAGCCCCACGGTGGGCG
Lys_tRNA42    GCCCGTCTAGCTCAGTCG-----GTAGAGCGCAAGGCTCTTAACCTTGTGGTCGTGGGTTCGAGCCCCACGGTGGGCG
Lys_tRNA53    GCCCGTCTAGCTCAGTCG-----GTAGAGCGCAAGGCTCTTAACCTTGTGGTCGTGGGTTCGAGCCCCACGGTGGGCG
Lys_tRNA34    GCCCGTCTAGCTCAGTCG-----GTAGAGCGCAAGGCTCTTAACCTTGTGGTCGTGGGTTCGAGCCCCACGGTGGGCG
Lys_tRNA42    GCCCGTCTAGCTCAGTCG-----GTAGAGCGCAAGGCTCTTAACCTTGTGGTCGTGGGTTCGAGCCCCACGGTGGGCG
Lys_tRNA22    GCCCGTCTAGCTCAGTCG-----GTAGAGCGCAAGGCTCTTAACCTTGTGGTCGTGGGTTCGAGCCCCACGGTGGGCG
Lys_tRNA44    GCCCGTCTAGCTCAGTCG-----GTAGAGCGCAAGGCTCTTAACCTTGTGGTCGTGGGTTCGAGCCCCACGGTGGGCG
Lys_tRNA18    GCCCGTCTAGCTCAGTCG-----GTAGAGCGCAAGGCTCTTAACCTTGTGGTCGTGGGTTCGAGCCCCACGGTGGGCG
Lys_tRNA34    GCCCGTCTAGCTCAGTCG-----GTAGAGCGCAAGGCTCTTAACCTTGTGGTCGTGGGTTCGAGCCCCACGGTGGGCG
Lys_tRNA35    GCCCGTCTAGCTCAGTCG-----GTAGAGCGCAAGGCTCTTAACCTTGTGGTCGTGGGTTCGAGCCCCACGGTGGGCG
Lys_tRNA50    GCCCGTCTAGCTCAGTCG-----GTAGAGCGCAAGGCTCTTAACCTTGTGGTCGTGGGTTCGAGCCCCACGGTGGGCG
Lys_tRNA23    GCCCGTCTAGCTCAGTCG-----GTAGAGCGCAAGGCTCTTAACCTTGTGGTCGTGGGTTCGAGCCCCACCGGTGGCCG
Lys_tRNA24    GCCCGTCTAGCTCAGTCG-----GTAGAGCGCAAGGCTCTTAACCTTGTGGTCGTGGGTTCGAGCCCCACGGTGGGCG
Lys_tRNA25    GCCCGTCTAGCTCAGTCG-----GTAGAGCGCAAGGCTCTTAACCTTGTGGTCGTGGGTTCGAGCCCCACGGTGGGCG
Lys_tRNA33    GCCCGTCTAGCTCAGTCG-----GTAGAGCGCAAGGCTCTTAACCTTGTGGTCGTGGGTTCGAGCCCCACGGTGGGCG
Lys_tRNA11    GCCCGTCTAGCTCAGTCG-----GTAGAGCGCAAGGCTCTTAACCTTGTGGTCGTGGGTTCGAGCCCCACGGTGGGCG
Lys_tRNA41    GCCCGTCTAGCTCAGTCG-----GTAGAGCGCAAGGCTTTTAACCTTGTGGTCGTGGGTTCGAGCCCCACGGTGGGCG
Lys_tRNA9_    GCCGACCTAGCTCAGT-G-----GTAGAGCGCGTGGCTTTTAACCACGTGGTCGTGGGTTCGATCCCCACGGTCGGCG
Lys_tRNA8_    GCCGACCTAGCTCAGT-G-----GTAGAGCGCGTGGCTTTTAACCACGTGGTCGTGGGTTCGATCCCCACGGTCGGCG
Lys_tRNA46    GCCGTCCTAGCTCAGT-G-----GTAGAGCGCGTGGCTTTTAACCACGTGGTCGTGGGTTCGATCCCCACGGACGGCG
Lys_tRNA10    GCCGTCCTAGCTCAGT-G-----GTAGAGCGCGTGGCTTTTAACCACGTGGTCGTGGGTTCGATCCCCACGGACGGCG
Lys_tRNA26    GCCGTCCTAGCTCAGT-G-----GTAGAGCGCGTGGCTTTTAACCACGTGGTCGTGGGTTCGATCCCCACGGACGGCG
Lys_tRNA24    GCCGTCCTAGCTCAGT-G-----GTAGAGCGCGTGGCTTTTAACCACGTGGTCGTGGGTTCGATCCCCACGGACGGCG
Lys_tRNA36    GCCGTCTTAGCTCAGCCG-----GTAGAGCGCATGGCTTTTAACCATGTGGTCGTGGGTTCGATTCCCACAGACGGCG
Lys_tRNA34    GCCGTCCTAGCTCAGTCG-----GTAGAGCGCACGGCTTTTAACCGTGTGGTCGTGGGTTCGAATCCCACGGACGGCG
Lys_tRNA7_  GCGGACCTATAGCTCAGCGGTAGGCGTAGAGCGTGTGGCTTTTAACCATGTGGTCGTGGGTTTGATCCCAACGGTCGGCG
Lys_tRNA18    GGGTGTATAGCTCAGTTG-----GTAGAGCATTGGGCTTTTAACCTAATGGTCGCAGGTTCAAGTCCTGACGTGACATCCT
Lys_tRNA24    GGGTGTATAGCTCAGTTG-----GTAGAGCATTGGGCTTTTAACCTAATGGTCGCAGGTTCAAGTCCTGCTATACCCA
```

```
Met_tRNA74  AGCGGAGTAGAGCAGTTTGGTA--GCTCACGAGGCTCATAACC-TTGAGGTCACGGGTTCGATTCCCG-TCTCCGCAC
Met_tRNA97  AGCGGAGTAGAGCAGTTTGGTA--GCTCACGAGGCTCATAACC-TTGAGGTCACGGGTTCGATTCCCG-TCTCCGCAC
Met_tRNA60  AGCGGAGTAGAGCAGTTTGGTA--GCTCACGAGGCTCATAACC-TTGAGGTCACGGGTTCGATTCCCG-TCTCCGCAC
Met_tRNA13  AGCGGAGTAGAGCAGTTTGGTA--GCTCACGAGGCTCATAACC-TTGAGGTCACGGGTTCGATTCCCG-TCTCCGCAC
Met_tRNA79  AGCGGAGTAGAGCAGTTTGGTA--GCTCACGAGGCTCATAACC-TTGAGGTCACGGGTTCGATTCCCG-TCTCCGCAC
Met_tRNA87  AGCGGAGTAGAGCAGTTTGGTA--GCTCACGAGGCTCATAACC-TTGAGGTCACGGGTTCGATTCCCG-TCTCCGCAC
Met_tRNA51  AGCGGGGTAGAGGAATTGGTCA--ACTCATCAGGCTCATAACC-TGAAGACTGCAGGTTCGAATCCTG-TCCCCGCCT
Met_tRNA53  AGCGGGGTAGAGGAATTGGTCA--ACTCATCAGGCTCATAACC-TGAAGACTGCAGGTTCGAATCCTG-TCCCCGCCT
Met_tRNA55  AGCGGGGTAGAGGAATTGGTCA--ACTCATCAGGCTCATAACC-TGAAGACTGCAGGTTCGAATCCTG-TCCCCGCCT
Met_tRNA22      GGATAGAACAGTTTGGTA--GCTGACAAGGCTCATAACC-GTGGGGTTGCGGGTTCGATTCCCACTACCCG
Met_tRNA57      GGATAGAACAGTTTGGTA--GCTGACAAGGCTCATAACC-GTGGGGTTGCGGGTTCGATTCCCACTACCCG
Met_tRNA96      GGATAGAACAGTTTGGTA--GCTGACAAGGCTCATAACC-GTGGGGTTGCGGGTTCGATTCCCACTACCCG
Met_tRNA60  GGGGTGGTGGCGCAGTTGGCTA--GCGCGTAGGTCTCATAATC-CTGAGGTCGAGAGTTCGAGCCTCTCTCACCCCA
Met_tRNA56  GGGGTGGTGGCGCAGTTGGCTA--GCGCGTAGGTCTCATAATC-CTGAGGTCGAGAGTTCGAGCCTCTCTCACCCCA
Met_tRNA31  GGGGTGGTGGCGCAGTTGGCTA--GCGCGTAGGTCTCATAATC-CTGAGGTCGAGAGTTCGAGCCTCTCTCACCCCA
Met_tRNA4_  GGGGTGGTGGCGCAGTTGGCTA--GCGCGTAGGTCTCATAATC-CTGAGGTCGAGAGTTCGAGCCTCTCTCACCCCA
Met_tRNA20  GGGGTGGTGGCGCAGTTGGCTA--GCGCGTAGGTCTCATAATC-CTGAGGTCGAGAGTTCGAGCCTCTCTCACCCCA
Met_tRNA35  GGGGTGGTGGCGCAGTTGGCTA--GCGCGTAGGTCTCATAATC-CTGAGGTCGAGAGTTCGAGCCTCTCTCACCCCA
Met tRNA24  GGGGTGGTGGCGCAGTTGGCTA--GCGCGTAGGTCTCATAATC-CTGAGGTCGAGAGTTCGAGCCTCTCTCACCCCA
Met_tRNA34  GGGGTGGTGGCGCAGTTGGCTA--GCGCGTAGGTCTCATAATC-CTGAGGTCGAGAGTTCGAGCCTCTCTCACCCCA
Met_tRNA35  GGGGTGGTGGCGCAGTTGGCTA--GCGCGTAGGTCTCATAATC-CTGAGGTCGAGAGTTCGAGCCTCTCTCACCCCA
Met_tRNA31  GGGGTGGTGGCGCAGTTGGCTA--GCGCGTAGGTCTCATAATC-CTGAGGTCGAGAGTTCGAGCCTCTCTCACCCCA
Met_tRNA30  GGGGTGGTGGCGCAGTTGGCTA--GCGCGTAGGTCTCATAATC-CTGAGGTCGAGAGTTCGAGCCTCTCTCACCCCA
Met_tRNA49  GGGGTGGTGGCGCAGTTGGCTA--GCGCGTAGGTCTCATAATC-CTGAGGTCGAGAGTTCGAGCCTCTCTCACCCCA
Met_tRNA55  TTTGTGGTGGCGCAGTTGGCTA--GCGCGTAGGTCTCATAATC-CTGAGGTCGAGAGTTCGAGCCTCTCTCCCCCCAAT
Met_tRNA32  GGGGTGGTGGTGCAGTTGGCTA--GCGCGTAGGTCTCATAATC-CTGAGGTCTA-AGTTCGATCCCCTCTCACCCCA
Met_tRNA21  GGGGTGGTGGCGCAGTTGGCTA--GCGCGTAGGTCTCATAATC-CTGAGGTCGAGAGTTCGAGCCTCAC---CCCAAATTT
Met_tRNA34  ATCAGAGTGGCGCAGCGGAA----GCGTGGTGGGCCCATAACC-CACAGGTCTCAGGATCGAAACCCG-GCTCTGATA
Met_tRNA37  ATCAGAGTGGCGCAGCGGAA----GCGTGGTGGGCCCATAACC-CACAGGTCCCAGGATCAAAACCTG-GCTCTGATA
Met_tRNA66  ATCAGAGTGGCGCAGCGGAA----GCGTGGTGGGCCCATAACC-CACAGGTCCCAGGATCGAAACCTG-GCTCTGATA
Met_tRNA1_  ATCAGAGTGGCGCAGCGGAA----GCGTGGTGGGCCCATAACC-CACAGGTCCCAGGATCGAAACCTG-GCTCTGATA
Met_tRNA7_  ATCAGAGTGGCGCAGCGGAA----GCGTGGTGGGCCCATAACC-CACAGGTCCCAGGATCGAAACCTG-GCTCTGATA
Met_tRNA75  ATCAGAGTGGCGCAGCGGAA----GCGTGGTGGGCCCATAACC-CACAGGTCCCAGGATCGAAACCTG-GCTCTGATA
Met_tRNA70  ATCAGAGTGGCGCAGCGGAA----GCGTGGTGGGCCCATAACC-CACAGGTCCCAGGATCGAAACCTG-GCTCTGATA
Met_tRNA13  ATCAGAGTGGCGCAGCGGAA----GCGTGGTGGGCCCATAACC-CACAGGTCCCAGGATCGAAACCTG-GCTCTGATA
Met_tRNA62  ATCAGAGTGGCGCAGCGGAA----GCGTGGTGGGCCCATAACC-CACAGGTCCCAGGATCGAAACCTG-GCTCTGATA
Met_tRNA61  ATCAGAGTGGCGCAGCGGAA----GCGTGGTGGGCCCATAACC-CACAGGTCCCAGGATCGAAACCTG-GCTCTGATA
Met_tRNA38  ATCAGAGTGGCGCAGCGGAA----GCGTGGTGGGCCCATAACC-CACAGGTCCCAGGATCGAAACCTG-GCTCTGATA
Met tRNA28  ATCAGAGTGGCGCAGCGGAA----GCGTGGTGGGCCCATAACC-CACAGGTCCCAGGATCGAAACCTG-GCTCTGATA
Met_tRNA10   GCCTACTTAACTCAGTTGTTA--GAGTATTGCTTTCATA-CGGCGGGAGTCATTGGTTCAAATCCAATAGTAGGTA
Met_tRNA3_   GCCTACTTAACTCAGTGGTCA--GAGTATTGCTTTCATA-CGGCGGGAGTCATTGGTTCAAATCCAATAGTAGGTA
Met_tRNA35   GCCTACTTAACTCAGTGGTTA--GAGTATTGCTTTCATA-CGGCGGGAGTCATTGGTTCAAATCCAATAGTAGGTA
Met_tRNA12   GCCTACTTAACTCAGTGGTTA--GAGTATTGCTTTCATA-CGGCGGGAGTCATTGGTTCAAATCCAATAGTAGGTA
Met_tRNA61   GCCTACTTAACTCAGTGGTTA--GAGTATTGCTTTCATA-CGGCGGGAGTCATTGGTTCAAATCCAATAGTAGGTA
Met_tRNA31   GCCTACTTAACTCAGTGGTTA--GAGTATTGCTTTCATA-CGGCGGGAGTCATTGGTTCAAATCCAATAGTAGGTA
Met_tRNA23   GCCTACTTAACTCAGTGGTTA--GAGTATTGCTTTCATA-CGGCGGGAGTCATTGGTTCAAATCCAATAGTAGGTA
Met_tRNA10   GCCTACTTAACTCAGTGGTTA--GAGTATTGCTTTCATA-TGACGGGAGTCATTGGTTCAAATCCAATAGTAGGTA
Met_tRNA15   GCCTACTTAACTCAGTGGTTA--GAGTATTGCTTTCATA-TGGCGGGAGTCATTGGTTCAAATCCAATAGTAGGTA
Met_tRNA27   ACCTACTTGACTCAGCGGTTA--GAGTATCGCTTTCATA-CGGCGAGAGTCATTGGTTCAAATCCAATAGTAGGTA
Met_tRNA32   GCATCCATGGCTAAATGGTTA--AAGCGCCCAACTCATAATTGGTAAATTTGCGGGTTCAATTCCTGCTGGATGCA
Met_tRNA4_   GCATCCATGGCTGAATGGTTA--AAGCGCCCAACTCATAATTGGTAAATTTGCGGGTTCAATTCCTGCTGGATGCA
Met_tRNA53   GCATCCATGGCTGAATGGTTA--AAGCGCCCAACTCATAATTGGTAAATTTGCGGGTTCAATTCCTGCTGGATGCA
Met_tRNA82   GCATCCATGGCTGAATGGTTA--AAGCGCCCAACTCATAATTGGTAAATTTGCGGGTTCAATTCCTGCTGGATGCA
Met_tRNA94   GCATCCATGGCTGAATGGTTA--AAGCGCCCAACTCATAATTGGTAAATTTGCGGGTTCAATTCCTGCTGGATGCA
Met_tRNA14   GCATCCATGGCTGAATGGTTA--AAGCGCCCAACTCATAATTGGTAAATTTGCGGGTTCAATTCCTGCTGGATGCA
Met_tRNA14   GCATCCATGGCTGAATGGTTA--AAGCGCCCAACTCATAATTGGTAAATTTGCGGGTTCAATTCCTGCTGGATGCA
Met_tRNA68   GCATCCATGGCTGAATGGTTA--AAGCGCCCAACTCATAATTGGTAAATTTGCAGGTTCAATTCCTGCTGGATGCA
```

```
               AA      DA    DL    DA   ACA   ACL    ACA    VR  TA       TL    TA     AA
Phe_tRNA31  GCCTTGATGGTGAAATGGTAGACACGCGAGACTGAAAATCTCGTGCAAAGAGCGTGGAGGTTCGAGTCCTCTTCAAGGCA
Phe_tRNA59  GTCAGGATAGATCAGTTGGTAGAGCAGAGGACTGAAAATCC---TCGTGTCACCAG----TTCAAATCTGGTTCCTGGCA
Phe_tRNA33  GTCAGGATAGCTCAGTTGGTAGAGCAGAGGACTGAAAATCC---TCGTGTCACCAG----TTCAAATCTGGTTCCTGGCA
Phe_tRNA76  GTCAGGATAGCTCAGTTGGTAGAGCAGAGGACTGAAAATCC---TCGTGTCACCAG----TTCAAATCTGGTTCCTGGCA
Phe_tRNA11  GTCAGGATAGCTCAGTTGGTAGAGCAGAGGACTGAAAATCC---TCGTGTCACCAG----TTCAAATCTGGTTCCTGGCA
Phe_tRNA14  GTCAGGATAGCTCAGTTGGTAGAGCAGAGGACTGAAAATCC---TCGTGTCACCAG----TTCAAATCTGGTTCCTGGCA
Phe_tRNA37  GTCAGGATAGCTCAGTTGGTAGAGCAGAGGACTGAAAATCC---TCGTGTCACCAG----TTCAAATCTGGTTCCTGGCA
Phe_tRNA83  GTGGGGATAGCTCAGTTGGGAGAGCGCCAGACTGAAGATCT---GAAGGTCGCGTG----TTCGATCCACGCTCACCGCA
Phe_tRNA29  GCGGGGATAGCTCAGTTGGGAGAGCGTCATACTGAAGATCT---GAAGGTCGCGTG----TTCGATCCACGCTCACCGCA
Phe_tRNA44  GCGGGGATAGCTCAGTTGGGAGAGCGTCAGACTGAAGATCT---GAAGGTCGCGTG----TTCGATCCACGCTCACCGCA
Phe_tRNA10  GCGGGGATAGCTCAGTTGGGAGAGCGTCAGACTGAAGATCT---GAAGGTCGCGTG----TTCGATCCACGCTCACCGCA
Phe_tRNA24  GCGGGGATAGCTCAGTTGGGAGAGCGTCAGACTGAAGATCT---GAAGGTCGCGTG----TTCGATCCACGCTCACCGCA
Phe_tRNA10  GCGGGGATAGCTCAGTTGGGAGAGCGTCAGACTGAAGATCT---GAAGGTCGCGTG----TTCGATCCACGCTCACCGCA
Phe_tRNA9_  GCGGGGATAGCTCAGTTGGGAGAGCGTCAGACTGAAGATCT---GAAGGTCGCGTG----TTCGATCCACGCTCACCGCA
Phe_tRNA22  GCGGGGATAGCTCAGTTGGGAGAGCGTCAGACTGAAGATCT---GAAGGTCGCGTG----TTCGATCCACGCTCACCGCA
Phe_tRNA35  GCGGGGATAGCTCAGTTGGGAGAGCGTCAGACTGAAGATCT---GAAGGTCGCGTG----TTCGATCCACGCTCACCGCA
Phe_tRNA22  GCGGGGATAGCTCAGTTGGGAGAGCGTCAGACTGAAGATCT---GAAGGTCGCGTG----TTCGATCCACGCTCACCGCA
Phe_tRNA18  GCGGGGATAGCTCAGTTGGGAGAGCGTCAGACTGAAGATCT---GAAGGTCGCGTG----TTCGATCCACGCTCACCGCA
Phe_tRNA61  GCGGGGATAGCTCAGTTGGGAGAGCGTCAGACTGAAGATCT---GAAGGTCGCGTG----TTCGATCCACGCTCACCGCA
Phe_tRNA58  GCGGGGATAGCTCAGTTGGGAGAGCGTCAGACTGAAGATCT---GAAGGTCGCGTG----TTCGATCCACGCTCACCGCA
```

```
             AA      DA       DL     DA    ACA    ACL    ACA  VR    TA    TL    TA    AA
Pro_tRNA11    GGGCATTTGGTCT-AGTGGTATGATTCTCGCTTAGGGTGCGAGAGGTCCCGAATTCAATTCTCGGAA--TGCCCC
Pro_tRNA7_    GGGCATTTGGTCT-AGTGGTATGATTCTCGCTTAGGGTGCGAGAGGTCCCGAGTTCAATTCTCGGAA--TGCCCC
Pro_tRNA17    GGGCATTTGGTCT-AGTGGTATGATTCTCGCTTAGGGTGCGAGAGGTCCCGAGTTCAATTCTCGGAA--TGCCCC
Pro_tRNA20    GGGCATTTGGTCT-AGTGGTATGATTCTCGCTTAGGGTGCGAGAGGTCCCGAGTTCAATTCTCGGAA--TGCCCC
Pro_tRNA31    GGGCATTTGGTCT-AGTGGTATGATTCTCGCTTAGGGTGCGAGAGGTCCCGAGTTCAATTCTCGGAA--TGCCCC
Pro_tRNA18    GGGCATTTGGTCT-AGTGGTATGATTCTCGCTTAGGGTGCGAGAGGTCCCGAGTTCAATTCTCGGAA--TGCCCC
Pro_tRNA29    GGGCATTTGGTCT-AGTGGTATGATTCTCGCTTAGGGTGCGAGAGGTCCCGAGTTCAATTCTCGGAA--TGCCCC
Pro_tRNA31    GGGCATTTGGTCT-AGTGGTATGATTCTCGCTTAGGGTGCGAGAGGTCCCGAGTTCAATTCTCGGAA--TGCCCC
Pro_tRNA68    GGGCATTTGGTCT-AGTGGTATGATTCTCGCTTAGGGTGCGAGAGGTCCCGAGTTCAATTCTCGGAA--TGCCCC
Pro_tRNA6_    GGGCATTTGGCCT-AGTGGTATGATTCTCGCTTAGGGTGCGAGAGGTCCCGAGTTCAATTCTCGGAA--TGCCCC
Pro_tRNA22    GGGCATTTGGTCT-AGTGGTATGATTCTCGCTTAGGGTGCGAGAGGTCCCGAGTTCAATTCTCGGAA--TGCCCC
Pro_tRNA24    GGGCATTTGGTCT-AGTGGTATGATTCTCGCTTTGGGTGCGAGAGGTCCCGAGTTCAATTCTCGGAA--TGCCCC
Pro_tRNA26    GGGCATTTGGTCT-AGTGGTATGATTCTCGCTTTGGGTGCGAGAGGTCCCGAGTTCGATTCTCGGAA--TGCCCC
Pro_tRNA65    GGGCATTTGGTCT-AGTGGTATGATTCTCGCTTTGGGTGCGAGAGGTCCCGAGTTCGATTCTCGGAA--TGCCCC
Pro_tRNA25    GGGCATTTGGTCT-AGTGGTATGATTCTCGCTTTGGGTGCGAGAGGTCCCGAGTTCGATTCTCGGAA--TGCCCC
Pro_tRNA20    GGGCATTTGGTCT-AGTGGTATGATTCTCGCTTAGGGTGCGAGAGGTCCCGAGTTCAATTCTCGGAA--TACCCC
Pro_tRNA5_    GGGCGTTTGGTCT-AGTGGTATGATTCTCGCTTTGGGTGCGAGAGGTCGCGAGTTCGATTCTCGCAA--CGCCCC
Pro_tRNA19    GGGCGTTTGGTCT-AGTGGTATGATTCTCGCTTTGGGTGCGAGAGGTCCCGAGTTCGATTCTCGGAA--CGCCCC
Pro_tRNA23    GGGCGTTTGGTCT-AGTGGTATGATTCTCGCTTTGGGTGCGAGAGGTCCCGAGTTCGATTCTCGGAA--CGCCCC
Pro_tRNA2_    GGGTGTTTGGTCTTAGTGGCATGATTCTCGCTTCGGGTGCGAGAGGTCATGAGTTCGATTCTCGCAAAACACCCC
Pro_tRNA71    GGGTGTTTGGTCT-AGTGGTATGATTCTCGCTTCGGGTGCGAGAGGTCGCGAGTTCGATTCTCGCAA--CACCCC
Pro_tRNA1_    GGGTGTTTGGTCT-AGTGGTATGATTCTCGCTTCGGGTGCGAGAGGTCGTGAGTTCGATTCTCGCAA--CACCCC
Pro_tRNA38    GGGTGTTTGGTCT-AGTGGTATGATTCTCGCTTCGGGTGCGAGAAGTCGCGAGTTCGATTCTCGCAA--CACCCC
Pro_tRNA34    GGGTGTTTGGTCT-AGTGGTATGATTCTCGCTTCGGGTGCGAGAGGTCGCGAGTTCGATTCTCGCAA--CACCCC
Pro_tRNA46    GGGTGTTTGGTCT-AGTGGTATGATTCTCGCTTCGGGTGCGAGAGGTCGCGAGTTCGATTCTCGCAA--CACCCC
Pro_tRNA38    GGGTGTTTGGTCT-AGTGGTATGATTCTCGCTTCGGGTGCGAGAGGTCGCGAGTTCGATTCTCGCAA--CACCCC
Pro_tRNA20    GGGTGTTTGGTCT-AGTGGTATGATTCTCGCTTCGGGTGCGAGAGGTCGCGAGTTCGATTCTCGCAA--CACCCC
Pro_tRNA59    GGGTGTTTGGTCT-AGTGGTATGATTCTCGCTTCGGGTGCGAGAGGTCGGGAGTTCGATTCTCCCAA--CACCCC
Pro_tRNA15    GGGGATTTAGTCT-AGTGGTAAGATATTCGCTTAGGGAGCGAAAGGTCCCGAGTTCAATTCTCGGAA---TCCCCA
Pro_tRNA16    GGGGATTTAGTCT-AGTGGTAAGATATTCGCTTAGGGAGCGAAAGGTCCCGAGTTCAATTCTCGGAA---TCCCCA
Pro_tRNA29   AGGGATGTAGCGCAGCTTGGTAGCACGTTTGTTTTGGGTACAAAATGTCATGGGTTCAAATCTTGTCA---TCCCTG
Pro_tRNA58   ACGAGTGTAGCGCAGCTTGGTAGCGCGTTTGTTTTGGGTACAAAATGTCACGGGTTCAAATCCTGTCA---TCCCTA
Pro_tRNA93   AGGGATGTAGCGCAGCTTGGTAGCGTGTTTGTTTTGGGTACAAAATGTCACAGGTTCAAATCCTGTCA---TCCCTA
Pro_tRNA8_   AGGGATGTAGCGCAGCTTGGTAGCGCGTTTGTTTTGGGTACAAAATGTCACAGGATCAAATCCTGTCA---TCCCTA
Pro_tRNA54   AGGGATGTAGCGCAGCTTGGTAGCGCGTTTGTTTTGGGTACAAAATGTCACAGGTTCAAATCCTGTCA---TCCCTA
Pro_tRNA62   AGGGATGTAGCGCAGCTTGGTAGCGCGTTTGTTTTGGGTACAAAATGTCACAGGTTCAAATCCTGTCA---TCCCTA
Pro_tRNA15   AGGGATGTAGCGCAGCTTGGTAGCGCGTTTGTTTTGGGTACAAAATGTCACAGGTTCAAATCCTGTCA---TCCCTA
```

```
                  AA     DA    DL     DA   ACA  ACL  ACA        VR        TA    TL    TA    AA
Ser_tRNA27     ACTTACTTAAGT--GGCTAGTGCACCAAGGCTAGAGTCCTTGGGAC--------------CCAGG-TTCGAATCCTGGCGGGGGCA
Ser_tRNA1_         GGGGGTGT--AGCTCATATGGCTCGCTTCGCATGCGAGAGGC--------------ACGGGGTTCGATTCCCCGCACCTCCA
Ser_tRNA20   GAATATGACAAATTTGGGTTATCGGGCATGACTAGAAATCATGTGGGCTCTACC------CGCGCAGGTTCGAATCCTGCCGACCA
Ser_tRNA71  GTGGACGTGCCAGAGT--GGTTATTGGGCATGACTAGAAATCATGTGGGCTCTGCC------CGCGTAGGTTCGAATCCTGCCATCCACG
Ser_tRNA2_  GTGGTCGTGCCGGAGT--GGTTATCGGGCATGACTAGAAATCATGTGGGCTCTGCC------CGCGCAGGTTCGAATCCTGCCGACCACG
Ser_tRNA73  GTGGTCGTGCCGGAGT--GGTTATCGGGCATGACTAGAAATCATGTGGGCTCTGCC------CGCGCAGGTTCGAATCCTGCCGACCACG
Ser_tRNA12  GTGGTCGTGCCGGAGT--GGTTATCGGGCATGACTAGAAATCATGTGGGCTCTGCC------CGCGCAGGTTCGAATCCTGCCGACCACG
Ser_tRNA64  GTGGTCGTGCCGGAGT--GGTTATCGGGCATGACTAGAAATCATGTGGGCTTTGCC------CGCGCAGGTTCGAATCCTGCCGACCACG
Ser_tRNA43  GTGGTCGTGCCGGAGT--GGTTATCGGGCATGACTAGAAATCATGTGGGCTTTGCC------CGCGCAGGTTCGAATCCTGCCGACCACG
Ser_tRNA47  GTGGTCGTGCCGGAGT--GGTTATCGGGCATGACTAGAAATCATGTGGGCTTTGCC------CGCGCAGGTTCGAATCCTGCCGACCACG
Ser_tRNA40  GTGGTCGTGCCGGAGT--GGTTATCGGGCATGACTAGAAATCATGTGGGCTTTGCC------CGCGCAGGTTCGAATCCTGCCGACCACG
Ser_tRNA11  GTGGTCGTGCCGGAGT--GGTTATCGGGCATGACTAGAAATCATGTGGGCTTTGCC------CGCGCAGGTTCGAATCCTGCCGACCACG
Ser_tRNA42  GTGGTCGTGCCGGAGT--GGTTATCGGGCATGACTAGAAATCATGTGGGCTTTGCC------CGCGCAGGTTCGAATCCTGCCGACCACG
Ser_tRNA43  GTCGATATGTCCGAGT--------GGAGACAGACTCGAAATCTGTTGGGATTTGCC------TGCGCAGGTTCGAATCCTGCTGTCGACG
Ser_tRNA28  GTCGATATGTCCGAGT--GGTTAAGGAGGTAGACTCGAAATCTACTGGGCTTTGCC------TGCGCAGGTTCGAATCCTGCTGTCGACG
Ser_tRNA60  GTCGATATGTCCGAGT--GGTTAAGGAGGTAGACTCGAAATCTACTGGGCTCTGCC------CGCGCAGGTTCGAATCCTGCTGTCGACG
Ser_tRNA89  GTCGATATGTCCGAGT--GGTTAAGGAGACAGACTCGAAATCTGTTGGGCTTTGCC------TGCGCAGGTTCGAATCCTGCTGTCGACG
Ser_tRNA41  GTCGATATGTCCGAGT--GGTTAAGGAGACAGACTCGAAATCTGTTGGGCTTTGCC------TGCGCAGGTTCGAATCCTGCTGTCGACG
Ser_tRNA26  GTCGATATGTCCGAGT--GGTTAAGGAGACAGACTCGAAATCTGTTGGGCTTTGCC------TGCGCAGGTTCGAATCCTGCTGTCGACG
Ser_tRNA43  GTCGATATGTCCGAGT--GGTTAAGGAGACAGACTCGAAATCTGTTGGGCTTTGCC------TGCGCAGGTTCGAATCCTGCTGTCGACG
Ser_tRNA11  GTCGATATGTCCGAGT--AGTTAAGGAGACAGACTTGAAATCTGTTGGGCTACGCC------CGCGCAGGTTCGAACCCTGCTGTCGACG
Ser_tRNA26  GTCGATATGTCCGAGT--GGTTAAGGAGACAGACTTGAAATCTGTTGGGCTACGCC------CGCGCAGGTTCGAACCCTGCTGTCGACG
Ser_tRNA6_  GTCGATATGTCCGAGT--GGTTAAGGAGACAGACTTGAAATCTGTTGGGCTTCGCC------CGCGCAGGTTCGAACCCTGCTGTCGACG
Ser_tRNA3_  GTCGATATGTCCGAGT--GGTTAAGGAGACAGACTTGAAATCTGTTGGGCTTCGCC------CGCGCAGGTTCGAACCCTGCTGTCGACG
Ser_tRNA5_  GTCGATATGTCCGAGT--GGTTAAGGAGACAGACTTGAAATCTGTTGGGCTTCGCC------CGCGCAGGTTCGAACCCTGCTGTCGACG
Ser_tRNA2_  GTCGATATGTCCGAGT--GGTTAAGGAGACAGACTTGAAATCTGTTGGGCTTCGCC------CGCGCAGGTTCGAACCCTGCTGTCGACG
Ser_tRNA33  GACGCTTTGGCCGAGT--GGTTAAGGCGTGTGCCTGCTAAGTACATGGGGTCTCCC------CGCGAGAGTTCGAATCTCTCAGGCGTCG
Ser_tRNA18  GACGCTTTGGCCGAGT--GGTTAAGGCGTGTGCCTGCTAAGTACATGGGGTTTCCC------CGCGAGAGTTCGAATCTCTCAGGCGTCG
Ser_tRNA40  GACGCTTTGGCCGAGT--GGTTAAGGCGTGTGCCTGCTAAGTACATGGGGTTTCCC------CGCGAGAGTTCGAATCTCTCAGGCGTCG
Ser_tRNA20  GACGCTTTGGCCGAGT--GGTTAAGGCGTGTGCCTGCTAAGTACATGGGGTTTCCC------CGCGAGAGTTCGAATCTCTCAGGCGTCG
Ser_tRNA19  GACGCTTTGGCCGAGT--GGTTAAGGCGTGTGCCTGCTAAGTACATGGGGTTTCCC------CGCGAGAGTTCGAATCTCTCAGGCGTCG
Ser_tRNA12  GACGCTTTGGCCGAGT--GGTTAAGGCGTGTGCCTGCTAAGTACATGGGGTTTCCC------CGCGAGAGTTCGAATCTCTCAGGCGTCG
Ser_tRNA39  GACGCTTTGGCCGAGT--GGTTAAGGCGTGTGCCTGCTAAGTACATGGGGTTTCCC------CGCGAGAGTTCGAATCTCTCAGGCGTCG
Ser_tRNA33  GACGCTTTGGCCGAGT--GGTTAAGGCGTGTGCCTGCTAAGTACATGGGGTTTCCC------CGCGAGAGTTCGAATCTCTCAGGCGTCG
Ser_tRNA75  GACGCTTTGGCCGAGT--GGTTAAGGCGTGTGCCTGCTAAGTACATGGGGTTTCCC------CGCGAGAGTTCGAATCTCTCAGGCGTCG
Ser_tRNA5_  GACGCTTTGGCCGAGT--GGTTAAGGCGTGTGCCTGCTAAGTACATGGGGTTTCCC------CGCGAGAGTTCGAATCTCTCAGGCGTCG
Ser_tRNA25  GGAGAGATAGCTGAGT-GGACTAAAGCGGCGGATTGCTAATGCGTTGTACAATTTTTTTTTACCGAGGGTTCGAATCCCTCTCTTTCCG
Ser_tRNA28  GGAGAGATGGCTGAGT-GGACTAAAGCGGCGGATTGCTAATCCGTTGTACAATTTTTTTGT-ACCGAGGGTTCGAATCCCTCTCTTTCCG
Ser_tRNA62  GGAGAGATGGCTGAGT-GGACTAAAGCGGCGGATTGCTAATCCGTTGTACAATTTTTTTGT-ACCGAGGGTTCGAATCCCTCTCTTTCCG
Ser_tRNA15  GGAGAGATGGCTGAGT-GGACTAAAGCGGCGGATTGCTAATCCGTTGTACAATTTTTTTGT-ACCGAGGGTTCGAATCCCTCTCTTTCCG
Ser_tRNA26  GGAGAGATGGCTGAGT-GGACTAAAGCGGCGGATTGCTAATCCGTTGTACAATTTTTTTGT-ACCGAGGGTTCGAATCCCTCTCTTTCCG
Ser_tRNA31  GGAGAGATGGCTGAGT-GGACTAAAGCGGCGGATTGCTAATCCGTTGTACAATTTTTTTGT-ACCGAGGGTTCGAATCCCTCTCTTTCCG
Ser_tRNA83  GGAGAGATGGCTGAGT-GGACTAAAGCGGCGGATTGCTAATCCGTTGTACAATTTTTTTGT-ACCGAGGGTTCGAATCCCTCTCTTTCCG
Ser_tRNA89  GGAGAGATGGCTGAGT-GGACTAAAGCGGCGGATTGCTAATCCGTTGTACAATTTTTTTGT-ACCGAGGGTTCGAATCCCTCTCTTTCCG
Ser_tRNA10  GGAGAGATGGCCGAGC-GGTTCAAGGCGTAGCATTGGAACTGCTATGTAGACTTTTGTTT--ACCGAGGGTTCGAATCCCTCTCTTTCCG
Ser_tRNA36  GGAGAGATGGCCGAGC-GGTTCAAGGCGTAGCATTGGAACTGCTATGTAGACTTTTGTTT--ACCGAGGGTTCGAATCCCTCTCTTTCCG
Ser_tRNA5_  GGAGAGATGGCCGAGC-GGTTCAAGGCGTAGCATTGGAACTGCTATGTAGACTTTTGTTT--ACCGAGGGTTCGAATCCCTCTCTTTCCG
Ser_tRNA6_  GGAGAGATGGCCGAGC-GGTTCAAGGCGTAGCATTGGAACTGCTATGTAGACTTTTGTTT--ACCGAGGGTTCGAATCCCTCTCTTTCCG
Ser_tRNA52  GGATGGATGTCTGAGC-GGTTGAAAGAGTCGGTCTTGAAAACCGAAGTATTGATAGGAA-T-ACCGGGGGTTCGAATCCCTCTCCATCCG
Ser_tRNA55  GGATGGATGTCTGAGC-GGTTGAAAGAGTCGGTCTTGAAAACCGAAGTATTGATAGGAA-T-ACCGGGGGTTCGAATCCCTCTCCATCCG
Ser_tRNA17  GGAGAGATGGCTGAGT-GGTTGATAGCTCCGGTCTTGAAAACCGGTATAGTTCTACGAACT-ATCGAGGGTTCGAATCCCTCTCTCTCCT
Ser_tRNA75  GGAGAGATGGCTGAGT-GGTTGATAGCTCCGGTCTTGAAAACCGGTATAGTTCTAGGAACT-ATCGAGGGTTCGAATCCCTCTCTCTCCT
Ser_tRNA61  GGAGAGATGGCTGAGT-GGTTGATAGCTCCGGTCTTGAAAACCGGTATAGTTCTAGGAACT-ATCGAGGGTTCGAATCCCTCTCTCTCCT
Ser_tRNA14  GGAGAGATGGCTGAGT-GGTTGATAGCTCCGGTCTTGAAAACCGGTATAGTTCTAGGAACT-ATCGAGGGTTCGAATCCCTCTCTCTCCT
Ser_tRNA45  GGAGAGATGGCTGAGT-GGTTGATAGCTCCGGTCTTGAAAACCGGTATAGTTCTAGGAACT-ATCGAGGGTTCGAATCCCTCTCTCTCCT
Ser_tRNA80  GGAGAGATGGCTGAGT-GGTTGATAGCTCCGGTCTTGAAAACCGGTATAGTTCTAGGAACT-ATCGAGGGTTCGAATCCCTCTCTCTCCT
Ser_tRNA88  GGAGAGATGGCTGAGT-GGTTGATAGCTCCGGTCTTGAAAACCGGTATAGTTCTAGGAACT-ATCGAGGGTTCGAATCCCTCTCTCTCCT
Ser_tRNA53  GGAGAGATGGCTGAGT-GGTTGATAGCTCCGGTCTTGAAAACCGGTATAGTTCTAGGAACT-ATCGAGGGTTCGAATCCCTCTCTCTCCT
Ser_tRNA1_  GGAGAGATGGCTAAGT-GGTTGATAGCTCCGATCTTGAAT--CTAGGAACTAT-----------CGAGGGTTTGAATCCCTCTCTCTCCT
Ser_tRNA22  GGAGGTATGGCTGAGT-GGCTTAAGGCATTGGTTTGCTATACAAGAAGATTGT---------ATCATGGGTTCGAATCCCATTTCCTCCG
Ser tRNA4   GGAGGTATGGCTGAGT-GGCATAAGGCATTGGTTTGCTATACAAGAAGATTGT---------ATCATGGGTTCGAATCCCATTTCCTCCG
```

```
           AA   DA   DL   DA   ACA   ACL   ACA   VR   TA   TL   TA   AA
Thr_tRNA51  GCCCAGTTGGTTAGGTGGGAAAGTACA--AGGCTTGTAAGCATGTGGTC-TGGGGTTCGACTCCCCTTCTGCTCA
Thr_tRNA40  GCTTTCGTAGCTCAGTTGGTTAGAGCACCCGTTTAGTAAGCGGGAGGTCCTGA-GTTCGACTCTCAACGAAAGCA
Thr_tRNA28  GCTTTCGTAGCTCAGTTGGTTAGAGCACCCGTTTAGTAAGCGGGAGGTCTTGA-GTTCGACTCTCAACGAAAGCA
Thr_tRNA4_  GCTTTCGTAGCTCAGTTGGTTAGAGCACCCGTTTAGTAAGCGGGAGGTCTTGA-GTTCGACTCTCAACGAAAGCA
Thr_tRNA1_  GCTTTCGTAGCTCAGTTGGTTAGAGCACCCGTTTAGTAAGCGGGAGGTCTTGA-GTTCGACTCTCAACGAAAGCA
Thr_tRNA40  GCTTTCGTAGCTCAGTTGGTTAGAGCACCCGTTTAGTAAGCGGGAGGTCTTGA-GTTCGACTCTCAACGAAAGCA
Thr_tRNA66  GCTTTCGTAGCTCAGTTGGTTAGAGCACCCGTTTAGTAAGCGGGAGGTCTTGA-GTTCGACTCTCAACGAAAGCA
Thr_tRNA51  GCTTTCGTAGCTCAGTTGGTTAGAGCACCCGTTTAGTAAGCGGGAGGTCTTGA-GTTCGACTCTCAACGAAAGCA
Thr_tRNA71  GCTTTCGTAGCTCAGTTGGTTAGAGCACCCGTTTAGTAAGCGGGAGGTCTTGA-GTTCGACTCTCAACGAAAGCA
Thr_tRNA3_  GCTTTCGTAGCTCAGTTGGTTAGAGCACCCGTTTAGTAAGCGGGAGGTCTTGA-GTTCGACTCTCAACGAAAGCA
Thr_tRNA46  GCTCTCGTAGCTCAGTTGGTTAGAGCACCCGTTTAGTAAGCGGGAGGTCTTGA-GTTCGACTCTCAACGAGAGCA
Thr_tRNA57  GCTCTCGTAGCTCAGTTGGTTAGAGCACCCGTTTAGTAAGCGGGAGGTCTTGA-GTTCGACTCTCAACGAGAGCA
Thr_tRNA56  GCTTTCGTATCTCAGTTGGTTAGAGCACCCGTTTGGTAAGCGGGAGGTCTTGAAGTTCGACTCTCAACGAAAGCA
Thr_tRNA25  GCTTCCGTAGCATAGTGG--TAGTGCGTTCGCTTCGTAAGCGAAAGGTCGCGA-GTTCGATCCTCGCCGGGAGCT
Thr_tRNA48  GCTTCCGTAGCATAGTGG--TAGTGCGTTCGCTTCGTAAGCGAAAGGTCGCGA-GTTCGATCCTCGCCGGGAGCT
Thr_tRNA40  GCTTCCGTAGCATAGTGG--TAGTGCGTTCGCTTCGTAAGCGAAAGGTCGCGA-GTTCGATCCTCGCCGGGAGCT
Thr_tRNA18  GCTTCCGTAGCATAGTGG--TAGTGCGTTCGCTTCGTAAGCGAAAGGTCGCGA-GTTCGATCCTCGCCGGGAGCT
Thr_tRNA17  GCCTCCGTAGCATAGTGG--TAGTGCGTTCGCTTCGTAAGCGAAAGGTCGCGA-GTTCGATCCTCGCCGGGGGCT
Thr_tRNA40  GCCCCTATAGCTCAGTGG--TAGAGCGTCAGTCTTGTAAACTGAAGGTC-TGTAGTTCGATCCTGCATGGGGGCA
Thr_tRNA41  GCCCCTATAGCTCAGTGG--TAGAGCGTCAGTCTTGTAAACTGAAGGTC-TGTAGTTCGATCCTGCATGGGGGCA
Thr_tRNA74  GCCCCTATAGCTCAGTGG--TAGAGCGTCAGTCTTGTAAACTGAAGGTC-TGTAGTTCGATCCTGCATGGGGGCA
Thr_tRNA34  GCCCTTATAGCTCAGTGG--TAGAGCGTCAGTCTTGTAAACTGAAGGTC-CGTAGTTCGATCCTGCGTGGGGGCA
Thr_tRNA80  GCCCCTATAGCTCAGTGG--TAGAGCGTCAGTCTTGTAAACTGAAGGTC-CGTAGTTCGATCCTGCGTGGGGGCA
Thr_tRNA25  GCCCCTATAGCTCAGTGG--TAGAGCGTCAGTCTTGTAAACTGAAGGTC-CGTAGTTCGATCCTGCGTGGGGGCA
Thr_tRNA77  GCCCTTTTAACTCAGTGG-T-GGAGTAATGCCATGGTAAGGCATAAGTCAT-CGGTTCAAATCCGATAAAGGGCT
Thr_tRNA12  GCCCTTTTAACTCAGTGG-T-AGAGTAATGCCATGGTAAGGCATAAGTCAT-CGGTTCAAATCCGATAAAGGGCT
Thr_tRNA6_  GCCCTTTTAACTCAGTGG-T-AGAGTAATGCCATGGTAAGGCATAAGTCAT-CGGTTCAAATCCGATAAAGGGCT
Thr_tRNA55  GCCCTTTTAACTCAGTGG-T-AGAGTAATGCCATGGTAAGGCATAAGTCAT-CGGTTCAAATCCGATAAAGGGCT
Thr_tRNA23  GCCCTTTTAACTCAGTGG-T-AGAGTAATGCCATGGTAAGGCATAAGTCAT-CGGTTCAAATCCGATAAAGGGCT
Thr_tRNA76  GCCCTTTTAACTCAGTGG-T-AGAGTAATGCCATGGTAAGGCATAAGTCAT-CGGTTCAAATCCGATAAAGGGCT
Thr_tRNA12  GCCCTTTTAACTCAGTGG-T-AGAGTAATGCCATGGTAAGGCATAAGTCAT-CGGTTCAAATCCGATAAAGGGCT
Thr_tRNA48  GCCCACTTAGCTCAGAGG-TTAGAGCATCACATTTGTAATGCGAGGGTCAT-CGGTTCAAATCCGATAGTCGGCT
Thr_tRNA60  GCCCACTTAGCTCAGAGG-TTAGAGCATCGCATTTGTAATGCGAGGGTCAT-CGGTTCAAATCCAATAGTCGGCT
Thr_tRNA36  GCCCACTTAGCTCAGAGG-TTAGAGCATCGCATTTGTAATGCGAGGGTCAT-CGGTTCAAATCCGATAGTCGGCT
Thr_tRNA21  GCCCACTTAGCTCAGAGG-TTAGAGCATCGCATTTGTAATGCGAGGGTCAT-CGGTTCAAATCCGATAGTCGGCT
Thr_tRNA98  GCCCACTTAGCTCAGAGG-TTAGAGCATCGCATTTGTAATGCGAGGGTCAT-CGGTTCAAATCCGATAGTCGGCT
Thr_tRNA95  GCCCACTTAGCTCAGAGG-TTAGAGCATCGCATTTGTAATGCGAGGGTCAT-CGGTTCAAATCCGATAGTCGGCT
Thr_tRNA49  GCCCACTTAGCTCAGAGG-TTAGAGCATCGCATTTGTAATGCGAGGGTCAT-CGGTTCAAATCCGATAGTCGGCT
Thr_tRNA47  GCCCACTTAGCTCAGAGG-TTAGAGCATCGCATTTGTAATGCGAGGGTCAT-CGGTTCAAATCCGATAGTCGGCT
Thr_tRNA56  GCCCACTTAGCTCAGAGG-TTAGAGCATCGCATTTGTAATGCGAGGGTCAT-CGGTTCAAATCCGATAGTCGGCT
```

```
            AA        DA    DL        DA   ACA   ACL   ACA   VR   TA    TL     TA    AA
Trp_tRNA61  GGATCCGTGGCGCAAT--GGTAGCGCGTCTGACTCCAGATCAGAAGGTTGCGTCTTTCGATTCACGTCGGGTTTA
Trp_tRNA32  GGATCCGTGGCGCAAT--GGTAGCGCGTCTGACTCCAGATCAGAAGGTTGCGT-GTTCGATTCATGTCGGGTTCA
Trp_tRNA70  GGATCCGTGGCGCAAT--GGTAGCGCGTCTGACTCCAGATCAGAAGGTTGCGT-GTTCGATTCACGTCGGGTTCA
Trp_tRNA47  GGATCCGTGGCGCAAT--GGTAGCGCGTCTGACTCCAGATCAGAAGGTTGCGT-GTTCGATTCACGTCGGGTTCA
Trp_tRNA29  GGATCCGTGGCGCAAT--GGTAGCGCGTCTGACTCCAGATCAGAAGGTTGCGT-GTTCGATTCACGTCGGGTTCA
Trp_tRNA44  GGATCCGTGGCGCAAT--GGTAGCGCGTCTGACTCCAGATCAGAAGGTTGCGT-GTTCGATTCACGTCGGGTTCA
Trp_tRNA46  GGATCCGTGGCGCAAT--GGTAGCGCGTCTGACTCCAGATCAGAAGGTTGCGT-GTTCGATTCACGTCGGGTTCA
Trp_tRNA17  GGATCCGTGGCGCAAT--GGTAGCGCGTCTGACTCCAGATCAGAAGGTTGCGT-GTTCGATTCACGTCGGGTTCA
Trp_tRNA57  GGATCCGTGGCGCAAT--GGTAGCGCGTCTGACTCCAGATCAGAAGGTTGCGT-GTTCGATTCACGTCGGGTTCA
Trp_tRNA69  GGATCCGTGGCGCAAT--GGTAGCGCGTCTGACTCCAGATCAGAAGGTTGCGT-GTTCGATTCACGTCGGGTTCA
Trp_tRNA32  GGATCCGTGGCGCAAT--GGTAGCGCGTCTGACTCCAGATCAGAAGGTTGCGT-GTTCGATTCACGTCGGGTTCA
Trp_tRNA7_  GGATCCGTGGCGCAAT--GGTAGCGCGTCTGACTCCAGATCAGAAGGTTGCGT-GTTCGATTCACGTCGGGTTCA
Trp_tRNA6_  GGATCCGTGGCGCAAT--GGTAGCGCGTCTGACTCCAGATCAGAAGGTTGCGT-GTTCGATTCACGTCGGGTTCA
Trp_tRNA63  GCGCTCTTAGTTCAGTTCGGTAGAACGCGGGTCTCCAAAACCCGATGTCGTAG-GTTCAAATCCTACGGAGCGTG
Trp_tRNA55  GCGCTCTTAGTTCAGTTCGGTAGAACGCGGGTCTCCAAAACCCGATGTCGTAG-GTTCAAATCCTACAGAGCGTG
Trp_tRNA9_  GCGCTCTTAGTTCAGTTCGGTAGAACGCGGGTCTCCAAAACCCGATGTCGTAG-GTTCAAATCCTACAGAGCGTG
Trp_tRNA16  GCGCTCTTAGTTCAGTTCGGTAGAACGCGGGTCTCCAAAACCCGATGTCGTAG-GTTCAAATCCTACAGAGCGTG
Trp_tRNA94  GCGCTCTTAGTTCAGTTCGGTAGAACACGGGTCTCCAAAACCCGATGTCGTAG-GTTCAAATCCTACAGAGCGTG
```

AA DA DL DA ACA ACL ACA VR TA TL TA AA

```
Tyr_tRNA49  GGGAGTGTGGCCGAGCGGTCAAAAGCGACATGCCGTAAATCTGGTGAAGATTT-TATACGCAGGTTCAAATCATGCCTATCCCA
Tyr_tRNA34  GGGAGAGTGGCCGAGCGGTCAAAAGCGACAGACTGTAAATCTGTTGAAGGTTT-TCTACGTAGGTTCGAATCCTGCCTCTCCCA
Tyr_tRNA19  TCGTCGATGCCCGAGCGGTTAATGGGGACGGACTATAAATTCGTTGACGATATGTCTACGCTGGTTCAAATCTAGCTCGGCCCA
Tyr_tRNA14  GGGTCGATGCCCGAGCGGTTAATGGGGACGGACTGTAAATTCGTTGACAATATGTCTACGCTGGTTCAAATCCAGCTCGGCCCA
Tyr_tRNA25  GGGTCGATGCCCGAGCGGTTAATGGGGACGGACTGTAAATTCGTTGACAATATGTCTACGCTGGTTCAAATCCAGCTCGGCCCA
Tyr_tRNA57  GGGTCGATGCCCGAGCGGTTAATGGGGACGGACTGTAAATTCGTTGACAATATGTCTACGCTGGTTCAAATCCAGCTCGGCCCA
Tyr_tRNA79  GGGTCGATGCCCGAGCGGTTAATGGGGACGGACTGTAAATTCGTTGACAATATGTCTACGCTGGTTCAAATCCAGCTCGGCCCA
Tyr_tRNA8_  GGGTCGATGCCCGAGCGGTTAATGGGGACGGACTGTAAATTCGTTGACAATATGTCTACGCTGGTTCAAATCCAGCTCGGCCCA
Tyr_tRNA4_  GCGTCGATGCACGAGCGGTTAATGGGGACAGACTATAAAT-----------ATGTCTACGCTGGTTCAAATCCAGCTCGGCCA
Tyr_tRNA48  CCGACCTTAGCTCAGTTGGTAGA-GCGGAGGACTGTAGATCCTTAGG----------TCGCTGGTTCGAATCCGGCAGGTCGGA
Tyr_tRNA31  CCGACCTTAGCTCAGTTGGTAGA-GCGGAGGACTGTAGATCCTTAGG----------TCGCTGGTTCGAATCCGGCAGGTCGGA
Tyr_tRNA51  CCGACCTTAGCTCAGTTGGTAGA-GCGGAGGACTGTAGATCCTTAGG----------TCGCTGGTTCGAATCCGGCAGGTCGGA
Tyr_tRNA45  CCGACCTTAGCTCAGTTGGTAGA-GCGGAGGACTGTAGATCCTTAGG----------TCGCTGGTTCGAATCCGGCAGGTCGGA
Tyr_tRNA21  CCGACCTTAGCTCAGTTGGTAGA-GCGGAGGACTGTAGATCCTTAGG----------TCGCTGGTTCGAATCCGGCAGGTCGGA
Tyr_tRNA22  CCGACCTTAGCTCAGTTGGTAGA-GCGGAGGACTGTAGATCCTTAGG----------TCGCTGGTTCGAATCCGGCAGGTCGGA
Tyr_tRNA30  CCGACCTTAGCTCAGTTGGTAGA-GCGGAGGACTGTAGATCCTTAGG----------TCGCTGGTTCGAATCCGGCAGGTCGGA
Tyr_tRNA6_  CCGACCTTAGCTCAGTTGGTAGA-GCGGAGGACTGTAGATCCTTAGG----------TCGCTGGTTCGAATCCGGCAGGTCGGA
Tyr_tRNA82  CCGACCTTAGCTCAGTTGGTAGA-GCGGAGGACTGTAGATCCTTAGG----------TCGCTGGTTCGAATCCGGCAGGTCGGA
Tyr_tRNA3_  CCGACCTTAGCTCAGTTGGTAGA-GCGGAGGACTGTAGATCCTTAGG----------TCGCTGGTTCGAATCCGGCAGGTCGGA
Tyr_tRNA52  CCGACCTTAGCTCAGTTGGTAGA-GCGGAGGACTGTAGATCCTTAGG----------TCGCTGGTTCGAATCCGGCAGGTCGGA
Tyr_tRNA58  CCGACCTTAGCTCAGTTGGTAGA-GCGGAGGACTGTAGATCCTTAGG----------TCGCTGGTTCGAATCCGGCAGGTCGGA
Tyr_tRNA22  CCGACCTTAGCTCAGTTGGTAGA-GCGGAGGACTGTAGATCCTTAGG----------TCGCTGGTTCAAATCCGGCAGGTCGGA
```

```
                     AA      DA    DL          DA  ACA   ACL     ACA    VR  TA               TL     TA    AA
Val_tRNA30     GGTTTCGTGGTGTAGTTGGTTATCACGTCAGTCTAACACACTGAAGGTCTCCCG---------TTCGAAGCCAAGTGATTTTT
Val_tRNA48     GGTTTCGTGGTGTAGTTGGTTATCACGTCAATCTAACACACTGAAGGTCTCCGG---------TTCGAGTCCGGGCAAAGCCA
Val_tRNA49     GGTTTTGTGGTGTAGTTGGTTATCACGTCAGTCTAACACACTGAAGGTCTCCGG---------TTCGAGTCCGGGCGAAGCCA
Val_tRNA51     GGTTTCGTGGTGTAGTTGGTTATCACGTCAGTCTAACACACTGAAGGTCTCCGG---------TTCGAGTCCGGGCGAAGCCA
Val_tRNA65     GGTTTCGTGGTGTAGTTGGTTATCACGTCAGTCTAACACACTGAAGGTCTCCGG---------TTCGAGTCCGGGCGAAGCCA
Val_tRNA47     GGTTTCGTGGTGTAGTTGGTTATCACGTCAGTCTAACACACTGAAGGTCTCCGG---------TTCGAGTCCGGGCGAAGCCA
Val_tRNA16     GGTTTCGTGGTGTAGTTGGTTATCACGTCAGTCTAACACACTGAAGGTCTCCGG---------TTCGAGTCCGGGCGAAGCCA
Val_tRNA31     GGTTTCGTGGTGTAGTTGGTTATCACGTCAGTCTAACACACTGAAGGTCTCCGG---------TTCGAACCCGGGCGAAGCCA
Val_tRNA32     GGTTTCGTGGTGTAGTTGGTTATCACGTCAGTCTAACACACTGAAGGTCTCCGG---------TTCGAACCCGGGCGAAGCCA
Val_tRNA12     GGTTTCGTGGTGTAGTTGGTTATCACGTCAGTCTAACACACTGAAGGTCTCCGG---------TTCGAACCCGGGCGAAGCCA
Val_tRNA42     GGTGTCGTGGTGTAGTTGGTTATCACGTCAGTCTAACACACTGAAGGTCTCCGG---------TTCGAGTCCGGGCGACGCCA
Val_tRNA50     GGTGTCGTGGTGTAGTTGGTTATCACGTCAGTCTAACACACTGAAGGTCTCCGG---------TTCGAGTCCGGGCGACGCCA
Val_tRNA13     GGTGTCGTGGTGTAGTTGGTTATCACGTCAGTCTAACACACTGAAGGTCTCCGG---------TTCGAGTCCGGGCGACGCCA
Val_tRNA6_     GGTGTCGTGGTGTAGTTGGTTATCACGTCAGTCTAACACACTGAAGGTCTCCGG---------TTCGAGTCCGGGCGACGCCA
Val_tRNA9_     GGTGTCGTGGTGTAGTTGGTTATCACGTCAGTCTAACACACTGAAGGTCTCCGG---------TTCGAGTCCGGGCGACGCCA
Val_tRNA42     GGTGTCGTGGTGTAGTTGGTTATCACGTCAGTCTAACACACTGAAGGTCTCCGG---------TTCGAACCCGGGCGACGCCA
Val_tRNA30     GGTGTCGTGGTGTAGTTGGTTATCACGTCAGTCTAACACACTGAAGGTCTCCGG---------TTCGAACCCGGGCGACGCCA
Val_tRNA28     GGTGTCGTGGTGTAGTTGGTTATCACGTCAGTCTAACACACTGAAGGTCTCCGG---------TTCGAACCCGGGCGACGCCA
Val_tRNA23     GTTGCTGTGGTGTAGT-GGTTATCACGTCAGTCTTACACACTGAAGGTCTCCAG---------TTCGATCCTGGGCAGCAACA
Val_tRNA61     GTTGCTGTGGTGTAGT-GGTTATCACGTCAGTCTTACACACTGAAGGTCTCCAG---------TTCGATCCTGGGCAGCAACA
Val_tRNA33     GTTGCTGTGGTGTAGT-GGTTATCACGTCAGTCTTACACACTGAAGGTCTCCAG---------TTCGATCCTGGGCAGCAACA
Val_tRNA30     GTTGCTGTGGTGTAGT-GGTTATCACGTCAGTCTTACACACTGAAGGTCTCCAG---------TTCGATCCTGGGCAGCAACA
Val_tRNA69     GTCTGGGTGGTGTAGTTGGTGATCACGTTAGTCTCACACACTAAAGGTCTCCAG---------TTCGAACCTGGACTTAGACA
Val_tRNA36     GTCTGGGTGGTGTAGTTGGTCATCACGTTAGTCTCACACACTAAAGGTCCCCAG---------TTCGAACCTGGGCTCAGACA
Val_tRNA44     GTCTGGGTGGTGTAGTTGGTTATCACGTTAGTCTCACACACTAAAGGTCCCCAG---------TTCGAACCTGGGCTCAGACA
Val_tRNA13     GTCTGGGTGGTGTAGTTGGTTATCACGTTAGTCTCACACACTAAAGGTCCCCAG---------TTCGAACCTGGGCTCAGACA
Val_tRNA5_     GTCTGGGTGGTGTAGTTGGTTATCACGTTAGTCTCACACACTAAAGGTCCCCAG---------TTCGAACCTGGGCTCAGACA
Val_tRNA4_     GTCTGGGTGGTGTAGTTGGTTATCACGTTAGTCTCACACACTAAAGGTCCCCAG---------TTCGAACCTGGGCTCAGACA
Val_tRNA28     GTCTGGGTGGTGTAGTTGGTTATCACGTTAGTCTCACACACTAAAGGTCCCCAG---------TTCGAACCTGGGCTCAGACA
Val_tRNA66     GTCTGGGTGGTGTAGTTGGTTATCACGTTAGTCTCACACACTAAAGGTCCCCAG---------TTCGAACCTGGGCTCAGACA
Val_tRNA35     GTCTGGGTGGTGTAGTTGGTTATCACGTTAGTCTCACACACTAAAGGTCCCCAG---------TTCGAACCTGGGCTCAGACA
Val_tRNA36     GTCTGGGTGGTGTAGTTGGTTATCACGTTAGTCTCACACACTAAAGGTCCCCAG---------TTCGAACCTGGGCTCAGACA
Val_tRNA45     GTCTGGGTGGTGTAGTCGGTTATCACGCTAGTCTCACACACTAGAGGTCTCAGACATTATAATTTCGATTCTGTGTTATGCTGAATA
Val_tRNA59  AGGGATATAACTCAGCGGTAGAGTGTCACCT-----TGACGTGGTGGAAGTCATCAA---------TTCGAGCCTGATTATCCCTA
Val_tRNA14  AGGGATATAACTCAGCGGTAGAGTGTCACCT-----TGACGTGGTGGAAGTCATCAG---------TTCGAGCCTGATTATCCCTA
Val_tRNA65  AGGGATATAACTCAGCGGTAGAGTGTCACCT-----TGACGTGGTGGAAGTCATCAG---------TTCGAGCCTGATTATCCCTA
Val_tRNA77  AGGGATATAACTCAGCGGTAGAGTGTCACCT-----TGACGTGGTGGAAGTCATCAG---------TTCGAGCCTGATTATCCCTA
Val_tRNA47  AGGGATATAACTCAGCGGTAGAGTGTCACCT-----TGACGTGGTGGAAGTCATCAG---------TTCGAGCCTGATTATCCCTA
Val_tRNA41  AGGGATATAACTCAGCGGTAGAGTGTCACCT-----TGACGTGGTGGAAGTCATCAG---------TTCGAGCCTGATTATCCCTA
Val_tRNA3_  AGGGATATAACTCAGCGGTAGAGTGTCACCT-----TGACGTGGTGGAAGTCATCAG---------TTCGAGCCTGATTATCCCTA
Val_tRNA54  AGGGATATAACTCAGCGGTAGAGTGTCACCT-----TGACGTGGTGGAAGTCATCAG---------TTCGAGCCTGATTATCCCTA
Val_tRNA11  AGGGATATAACTCAGCGGTAGAGTGTCACCT-----TGACGTGGTGGAAGTCATCAG---------TTCGAGCCTGATTATCCCTA
Val_tRNA16  AGGGATATAACTCAGCGGTAGAGTGTCACCT-----TGACGTGGTGGAAGTCATCAG---------TTCGAGCCTGATTATCCCTA
Val_tRNA18  AGGGATATAACTCAGCGGTAGAGTGTCACCT-----TGACGTGGTGGAAGTCATCAG---------TTCGAGCCTGATTATCCCTA
Val_tRNA27  AGGGATATAACTCAGCGGTAGAGTGTCACCT-----TGACGTGGTGGAAGTCATCAG---------TTCGAGCCTGATTATCCCTA
Val_tRNA19  AGGGATATAACTCAGCGGTAGAGTGTCACCT-----TGACGTGGTGGAAGTCATCAG---------TTCGAGCCTGATTATCCCTA
Val_tRNA10  AGGGATATAACTCAGCGGTAGAGTGTCACCT-----TGACGTGGTAGAAGTCATCAG---------TTCGAGCCTGATTATCCCTA
Val_tRNA92  AGGGATATAACTCAGCGGTAGAGTGTCACCT-----TGACGTGGTAGAAGTCATCAG---------TTCGAGCCTGATTATCCCTA
Val_tRNA20  AGGGATATAACTCAGCGGTAGAGTGTCACCT-----TGACGTGGTAGAAGTCATCAG---------TTCGAGCCTGATTATCCCTA
Val_tRNA39  AGGGATATAACTCAGCGGTAGAGTGTCACCT-----TGACGTGGTGGAAGTCATCAG---------TTCGAGCCTAATTATCCCTA
```
